# Molecular Mechanisms Underlying Salt Tolerance in Maize: A Combined Transcriptome and Metabolome Analysis

**DOI:** 10.3390/plants14132031

**Published:** 2025-07-02

**Authors:** Shaoqi Ren, Tianhang Bai, Yaqi Ma, Yingjie Zhao, Jiabin Ci, Xuejiao Ren, Zhenyuan Zang, Chengqian Ma, Ruyi Xiong, Xinyao Song, Wei Yang, Weiguang Yang

**Affiliations:** College of Agronomy, Jilin Agricultural University, Changchun 130118, China; renshaoqi2021@163.com (S.R.); 18747198418@139.com (T.B.); 18364926513@163.com (Y.M.); 13944261506@163.com (Y.Z.); cjb6666@163.com (J.C.); rxj0342@163.com (X.R.); zhenyuanzang1989@163.com (Z.Z.); ghvj53@icloud.com (C.M.); xry173226@163.com (R.X.); song220621@163.com (X.S.)

**Keywords:** maize, transcriptome, metabolome, salt tolerance, molecular mechanisms

## Abstract

Maize (*Zea mays* L.) is one of the most important food crops. Salt stress can hinder crop growth and development, but the molecular mechanisms underlying maize’s response to salt tolerance remain unclear. In this study, we conducted comparative transcriptome, metabolome, and physiological analyses of a salt-tolerant maize inbred line (J1285) subjected to different NaCl concentrations during the seedling stage. The results demonstrated that, with increasing salt concentration, seedling growth parameters and antioxidant enzyme activities (SOD, POD, CAT) exhibited initially increases before subsequently decreasing, peaking at 50–150 mmol/L. Transcriptome data analysis revealed that the experimental groups subjected to 50, 100, 150, and 200 mmol/L treatments had 375, 1043, 2504, and 2328 differentially expressed genes (DEGs) compared to the control group, respectively. Additionally, through GO and KEGG analysis, we found that the DEGs were primarily enriched in the MAPK signaling pathway and plant hormone signal transduction, especially the abscisic acid (ABA) signaling pathway, both of which play instrumental roles in orchestrating the maize response to salt-induced stress. Transcription factors involved in the salt stress response, including WRKY, TIFY, bZIP, and bHLH, were identified. Metabolomic data analysis revealed that the experimental groups subjected to 50, 100, 150 and 200 mmol/L treatments had 44, 335, 278, and 550 differentially expressed metabolites (DEMs) compared to the control group, respectively. The DEMs were mainly enriched in metabolic pathways and the biosynthesis of secondary metabolites. Transcriptomics and metabolomics combined analysis were performed on J1285 seedling leaves, and it was found that the co-enrichment pathways included starch and sucrose metabolism, linoleic acid metabolism, α-linolenic acid metabolism, phenylpropanoid biosynthesis pathway, etc. Collectively, these results will aid in identifying resistance genes and elucidating the molecular mechanisms underlying salt tolerance for maize.

## 1. Introduction

Salt stress is a prevalent environmental factor that significantly impacts global crop production and impedes agricultural advancement [1,2]. Excessive soil salinity inhibits seed germination, root development, and seedling establishment, ultimately leading to diminished crop yield and quality [3]. Research indicates that with the increase in salt concentration, the growth indicators of maize, such as stem thickness, primary root length, and leaf area, significantly decline [4]. Furthermore, salt stress also results in a reduction in the biomass of maize plants, particularly leading to a notable decrease in the dry weight of roots and stems [5,6]. Salt stress can also disrupt the expression of genes related to photosynthesis, further inhibiting the process of photosynthesis [7]. The adverse effects of salt stress on plants primarily occur through three mechanisms: ionic stress, osmotic stress, and secondary stress [8]. While many terrestrial plants can tolerate low to moderate salinity, halophytes—plants that are naturally salt-tolerant—thrive in high-salt environments [9]. The detrimental effects of salinity on plants first present as short-term osmotic stress, which subsequently evolves into ion accumulation that induces phytotoxicity over time [10]. Salt stress can lead to the excessive accumulation of reactive oxygen species (ROS) within plant cells, which in turn triggers oxidative stress, damaging cellular structures and functions. Consequently, plants have evolved a series of complex antioxidant mechanisms, including enzymatic and non-enzymatic antioxidant systems, to maintain redox balance within cells [11,12]. Within maize plants, ROS, such as hydrogen peroxide (H_2_O_2_) and superoxide anions (O_2_^−^), can damage the structural integrity of cell membranes, resulting in lipid peroxidation and membrane injury, which is manifested by an increase in malondialdehyde (MDA) content [13,14]. At the same time, the activity of antioxidant enzymes, such as superoxide dismutase (SOD), peroxidase (POD), and catalase (CAT), may be inhibited under salt stress, further exacerbating oxidative damage [5,14]. Various endogenous plant hormones, including abscisic acid (ABA) [15], auxin, salicylic acid (SA), jasmonic acid (JA) [16], cytokinins [17], gibberellins, ethylene, and brassinosteroids (BR), play a crucial role in regulating the plant’s response to salt stress and enhancing salt tolerance [2].

Maize (*Zea mays* L.) is a widely cultivated, high-yielding crop recognized for its ease of management and extensive applications in agriculture and industry [18]. As a salt-sensitive species, maize is particularly vulnerable to salt stress. Recent studies have identified specific genes and regulatory factors enabling maize to adapt to saline environments. For instance, the type-A response regulator *ZmRR1* plays a critical role in regulating Cl^−^ exclusion in the shoots and underpins natural variation in salt tolerance among maize varieties [19]. Additionally, the bHLH transcription factor *ZmbHLH32* has been shown to enhance salt tolerance by directly up-regulating the expression of *ZmIAA9* [20]. Furthermore, *ZmEREB20*, a member of the maize AP2-ERF family, has been implicated in the regulation of salt tolerance [21]. WRKY transcription factors represent an important family involved in plant development, defense regulation, and stress responses. The wheat *TaWRKY24* enhances the salt tolerance of transgenic plants by increasing the K^+^/Na^+^ ratio [22]. The sweet potato *IbWRKY2* and grape *VvWRKY30* enhance the salt tolerance of transgenic plants by increasing proline and soluble sugar content [23,24]. The overexpression of tobacco *NtWRKY65* and wheat *TaWRKY17* significantly improves the salt tolerance of transgenic plants [25,26]. Notably, *ZmWRKY114* has been found to diminish salt stress tolerance and sensitivity to abscisic acid (ABA) in rice by modulating the ABA signaling pathway and the expression of stress response genes [27]. Additionally, studies suggest that some genes may regulate plant responses to salt stress by influencing the ABA and ROS pathways during plant growth and development [28,29,30].

Multi-omics technologies have emerged as powerful tools for studying plant systems, integrating data from genomics, transcriptomics, proteomics, and metabolomics. Given that gene expression changes over time in response to different stimuli, transcriptome analysis has shown significant potential for analyzing gene expression. Conversely, metabolomics provides valuable insights into plant physiology by examining various metabolites involved in different cellular processes [31]. The integration of transcriptome and metabolome analyses offers an effective approach for investigating the mechanisms underlying plant stress resistance. However, there is limited research on the role of salt stress resistance in maize seedlings. Numerous genes have been identified to play a role in regulating plant resistance to salt stress across various species. But the specific mechanisms underlying salt defense in maize have yet to be fully elucidated.

The purpose of this study is to identify key genes and metabolites involved in the salt stress response of the salt-tolerant maize inbred line J1285 under varying NaCl concentrations. This will be achieved through transcriptomic, metabolomic, and physiological analyses, aiming to elucidate the regulatory mechanisms underlying the salt response processes. These findings provide a theoretical basis for exploring salt-tolerant gene resources in maize and for breeding salt-tolerant maize varieties. This will further enhance the adaptability of maize to saline-alkali land, which is of great significance for ensuring stable growth in maize yield, expanding cultivation area, and alleviating the food crisis.

## 2. Results

### 2.1. Phenotypic and Physiological Responses of Maize to Salt Stress

**Figure 1:**
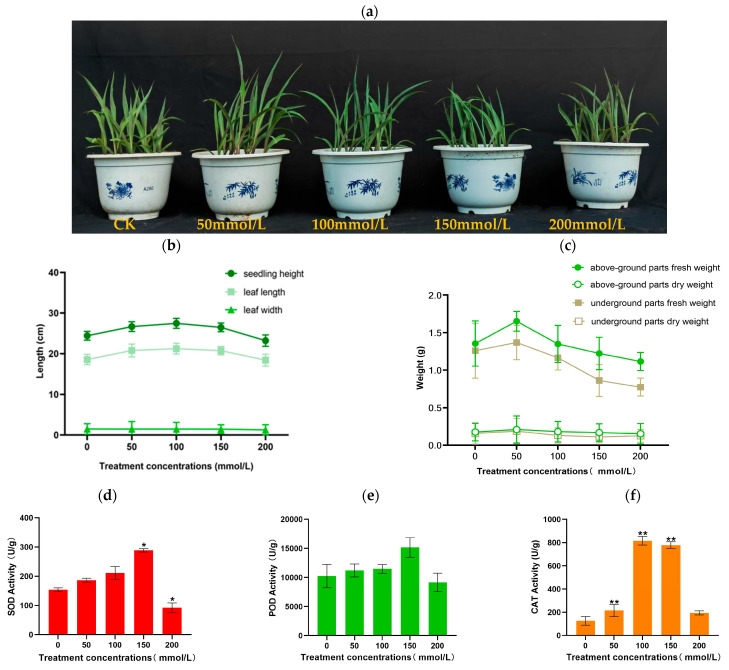
Effects of saline stress on the seedlings after 8 days. (**a**) Growth phenotype of maize cultivars with and without saline treatment. Scale bar: 10 cm. (**b**) Seedling height, leaf length, and leaf width. (**c**) Above-ground parts fresh weight, above-ground parts dry weight, underground parts fresh weight, underground parts dry weight. (**d**–**f**) SOD, POD, and CAT activity. Each bar chart represents the average ± SD of three biological replicates, * *p* < 0.05, **p < 0.01.

To assess the salt tolerance of inbred maize lines and their response to salt stress, seedlings at three leaves and a cusp period were cultivated. The control group was irrigated with sterilized deionized water (Group A), while the treated groups were exposed to NaCl concentrations of 50, 100, 150, and 200 mmol/L (Group B, C, D, and E) for 8 days. Observations of seedling morphology indicated that salt stress affected seedling growth (Figure 1a). Salt stress initially led to an increase in seedling height and leaf length, followed by a decrease (Figure 1b). However, leaf width remained unaffected. Both fresh and dry weights exhibited a pattern of increase-decrease under salt treatment compared to the control (Figure 1c). Under salt stress conditions, there was no significant difference in POD activity levels among the seedling lines. There were significant differences in the SOD content of groups D and E and the CAT activities of groups B, C, and D. Under salt stress, SOD, POD, and CAT activities all maintained a trend of first increasing and then decreasing (Figure 1d–f).

These results indicate that J1285 can produce a series of phenotypic and physiological responses to salt stress. Salt stress will inhibit the growth of corn seedlings. As the concentration increases, leaf length, leaf width, seedling height, fresh height, dry height, SOD, POD, and CAT all first increase and then decrease, and the above indicators will show different maximum values under different concentration conditions.

### 2.2. Transcriptomic Analysis of Maize in Response to Salt Stress

**Figure 2:**
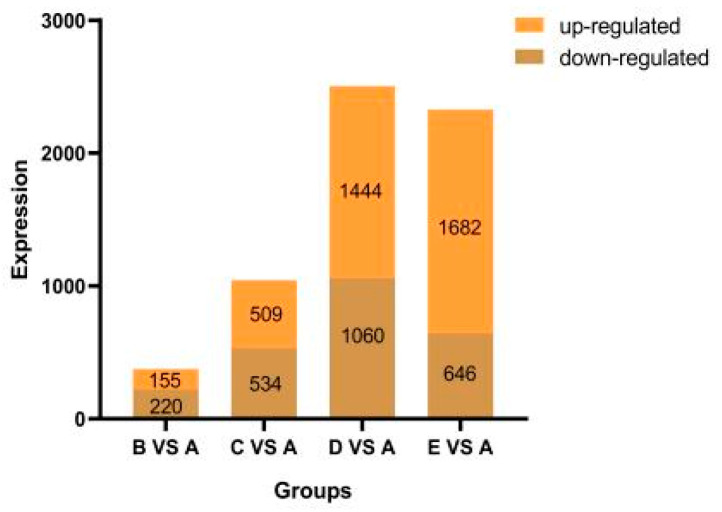
Number of DEGs after exposure to saline condition. A: CK, B:50 mmol/L, C:100 mmol/L, D: 150 mmol/L, E: 200 mmol/L.

To identify the genes responsible for salt stress tolerance, RNA-sequencing was conducted on three independent samples from both saline-treated and untreated control samples. A total of approximately 101.94 million raw sequencing reads were generated from Illumina HiSeq™ (Illumina, Shanghai, China)sequencing of the 15 samples (Table S2). Each sample had clean reads ranging from 48.41 to 79.04 million, with an average of 63.51 million. The clean reads had high quality, with at least 95.58% of bases having a quality score ≥ Q30, indicating suitability for subsequent differential gene expression analysis. PCA showed distinct regions based on treatment, highlighting the impact of saline stress on gene expression changes (Figure S1). DEGs were defined as those with at least a two-fold change and significant difference under saline stress compared to the control. A Venn diagram was then created (Figure 2) (Table S3). When comparing B vs. A, 375 DEGs were identified (155 genes up-regulated and 220 genes down-regulated). In the comparison of C vs. A, a total of 1043 DEGs were found (509 genes up-regulated and 534 genes down-regulated). Moving on to D vs. A, 2504 DEGs were identified (1444 genes up-regulated and 1060 genes down-regulated). Finally, in the comparison of E vs. A, 2328 DEGs were observed (1682 genes up-regulated and 646 genes down-regulated) (Figure 2).

The results showed that as the concentration increased, the number of DEGs also increased, with more genes being up-regulated than down-regulated. This highlights a distinct response to saline stress at the transcriptional level. These DEGs were considered to be salt stress response genes.

#### 2.2.1. GO and KEGG Enrichment Analysis of DEGs Involved in Salt Tolerance Response

**Figure 3:**
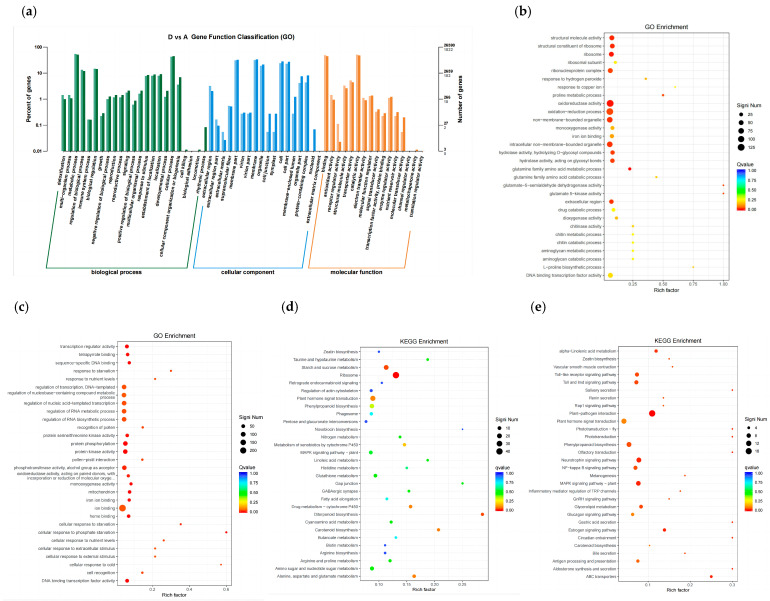
(**a**) Gene function classification. The light color represents DEGs and the dark color represents all genes. The right longitudinal axis is the number of genes in the classification and the left longitudinal axis is the proportion of the number of genes (differential genes/all genes) annotated to this function. (**b**) Bubble plots of the GO items in the GO enrichment analysis of up-regulated DEGs. (**c**) Bubble plots of the GO items in the GO enrichment analysis of down-regulated DEGs. (**d**) Bubble plots of the KEGG items in the GO enrichment analysis. (**e**) Bubble plots of the KEGG items in the GO enrichment analysis. Significant number represents the number of enriched genes. Qvalue represents the corrected *p* value.

The results revealed a significant difference in gene expression levels between Group A and D, as depicted in Figure 2. To gain a deeper insight into the saline-responsive pathways in maize, a combination of GO and KEGG analyses was employed to explore the functional enrichment and annotation of the DEGs [32]. Both approaches indicated that maize underwent modifications in its metabolic pathways following exposure to salt treatment. The DEGs were categorized into three functional groups: BP, CC, and MF, with a predominant association with BP, as illustrated in Figure 3a and Figure S2. It can be seen that the entries with more DEGs and all genes are concentrated in ‘metabolic process’, ‘establishment of localization’, ‘localization’, ‘regulation of biological process’, ‘cellular process’, ‘biological regulation’, and ‘response to stimulus’. The results show that maize responds to salt stress from three functional groups at the same time, among which the BP component plays more functions. The seven items concentrated in BP may be related to the response of maize to salt stress. They can be the focus of salt stress response.

The enrichment analysis of GO highlighted the significant biological processes in which DEGs were enriched under salt stress conditions (Figure 3b,c and Figure S3). DEGs were found to be enriched in various pathways, such as the GO category BP ‘oxidation-reduction process’ (GO:0055114) and MF ‘oxidoreductase activity’ (GO:0016491), which were enriched in the up-regulated DEGs of both groups C vs. A and D vs. A. In particular, there were down-regulated DEGs enriched principally in the BP ‘protein phosphorylation’ (GO:0006468), ‘regulation of nucleic acid-templated transcription’ (GO:1903506), ‘regulation of RNA biosynthetic process’ (GO:2001141), MF ‘protein kinase activity’ (GO:0004672), and ‘phosphotransferase activity, alcohol group as acceptor’ (GO:0016773).

These results indicate that processes such as ‘oxidation-reduction process’, ‘oxidoreductase activity’, ‘intracellular part’, and ‘cofactor binding’ play a crucial role in promoting salt stress tolerance in maize.

The KEGG analysis revealed that several pathways were enriched in the up-regulated DEGs (Figure 3d and Figure S4a–c), including ‘plant hormone signal transduction’ (ko04075), ‘ribosome’ (ko03010), ‘phenylpropanoid biosynthesis’ (ko00940), ‘MAPK signaling pathway-plant’ (ko04016), and ‘arginine and proline metabolism’ (ko00330). On the other hand, numerous pathways were enriched in the down-regulated DEGs (Figure 3e and Figure S4d–f), such as ‘alpha-Linolenic acid metabolism’ (ko00592), ‘Amino sugar and nucleotide sugar metabolism’ (ko00520), ‘phenylpropanoid biosynthesis (ko00940), ‘plant-pathogen interaction’ (ko04626), ‘Glycerolipid metabolism’ (ko00561), and ‘Neurotrophin signaling pathway’ (ko04722).

Overall, the functional enrichment and annotation of the DEGs indicated that several genes associated with various pathways, including salt stress response, may be involved in maize saline stress resistance. Genes related to ‘lipid metabolism’, ‘phenylalanine metabolism’, ‘alpha-Linolenic acid metabolism’, ‘sugar metabolism’, and ‘signal transduction’ that were particularly affected by saline stress in maize could have distinct roles in enhancing its resistance to saline stress.

In summary, the results of the functional enrichment and annotation of the DEGs showed the expression of several genes involved in various pathways, as well as salt stress responses, which may suggest their involvement in maize saline stress resistance.

#### 2.2.2. DEGs Involved in Plant Hormone Signal Transduction in Maize

**Figure 4:**
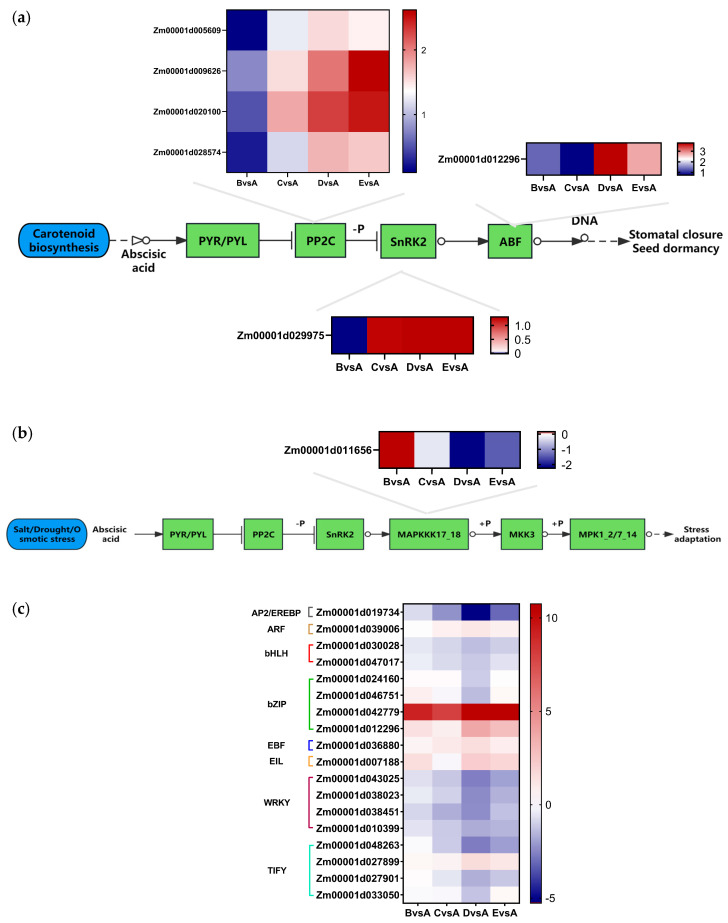
(**a**) The abscisic acid signal pathway in maize. (**b**) The MAPK signaling pathway in maize. (**c**) The TFs expression level in maize of salt treatment. A: CK, B:50 mmol/L, C:100 mmol/L, D: 150 mmol/L, E: 200 mmol/L.

The KEGG analysis results revealed 34 DEGs that were individually linked to plant hormone signal transduction (Table S4). This pathway encompasses various plant hormones such as auxin, cytokinin, gibberellin, abscisic acid, ethylene, brassinosteroid, jasmonic acid, and salicylic acid. Interestingly, common genes and hormone components were identified across all the hormone signaling pathways (Figure 4a). The results showed that some genes were also slightly induced in salt-treated maize seedlings. Notably, the genes *Zm00001d009626* (*ZmPP2C51*) and *Zm00001d020100* (*ZmPP2C11*) showed even stronger induction in maize seedlings.

In the abscisic acid (ABA) signaling pathway, seven genes showed differential expression, categorized into protein phosphatase 2C (PP2C), ABA-responsive element binding factor (ABF), and sucrose non-fermenting-1-related protein kinase 2 (SnRK2). Apart from group B vs. A, PP2C expression was up-regulated in other groups. In particular, *Zm00001d020100* (*ZmPP2C11*) and *Zm00001d009626* (*ZmPP2C51*) displayed significant expression variations under different salt treatments. In SnRK2 and ABF, distinct expression patterns of the same genes like *Zm00001d029975* (*ZmSnRK2.3*) and *Zm00001d012296* were more noticeable among the groups (Figure 4a). Moreover, the ABA hormone signaling pathway showed a strong correlation with the MAPK signaling pathway triggered by salt/cold and osmotic stress.

Notably, *Zm00001d005609*, *Zm00001d009626* (*ZmPP2C51*), *Zm00001d020100* (*ZmPP2C11*), and *Zm00001d0028574* were jointly involved in the ABA pathway and MAPK signaling pathway (Figure 4a,b). The close association between the ABA pathway and stress-induced MAPK signaling pathway warrants further investigation and research.

#### 2.2.3. DEGs Involved in MAPK Signaling Pathway in Maize

The KEGG analysis revealed that the MAPK signaling pathway enriched 19 DEGs (Table S5). Protein phosphorylation/dephosphorylation are fundamental mechanisms by which cells respond to changes in their external environment and regulate cellular function. Protein kinases catalyze the phosphorylation of proteins, allowing for the amplification of environmental signals and subsequent regulation of cellular physiological responses. The MAPK cascade pathway plays a crucial role in transmitting environmental stress signals. During periods of environmental stress, plants enhance these signals through the MAPKKK-MAPKK-MAPK triple phosphorylation cascade to trigger tolerance responses.

The KEGG analysis also demonstrated that salt stress impacts pathways involved in inducing salt tolerance through a series of signal transduction events. Of particular interest are the DEGs associated with MAPKKK17_18 (K20716) within the MAPK signaling pathway. Specifically, the expression of *Zm00001d011656* in the salt treatment showed a significant decrease with increasing salt stress concentration (Figure 4b). However, no significant expression changes were observed for MEKK1 (K13414), MKK2 (K20603), or MPK4 (K20600). It can be seen that the gene *Zm00001d011656* may have induced salt tolerance in the MAPK signaling pathway.

#### 2.2.4. Changes in Differentially Expressed TFs Under Salt Stress

Transcription factors (TFs) are critical regulators of gene expression, playing a pivotal role in controlling plant growth, development, and response to stress. By conducting a comparative analysis of gene expression profiles in inbred maize lines J1285 under varying NaCl concentrations, a total of 18 TFs from 8 TF families were identified (Table S6). In particular, the WRKY, TIFY, basic region-leucine zipper (bZIP), and basic helix-loop-helix (bHLH) families exhibited DEGs associated with salt tolerance in maize.

In summary, these TFs consistently showed varying expression levels throughout the duration of the salt treatment (Figure 4c), suggesting their potential regulatory role in response to salt stress.

#### 2.2.5. Transcriptomic WGCNA

**Figure 5:**
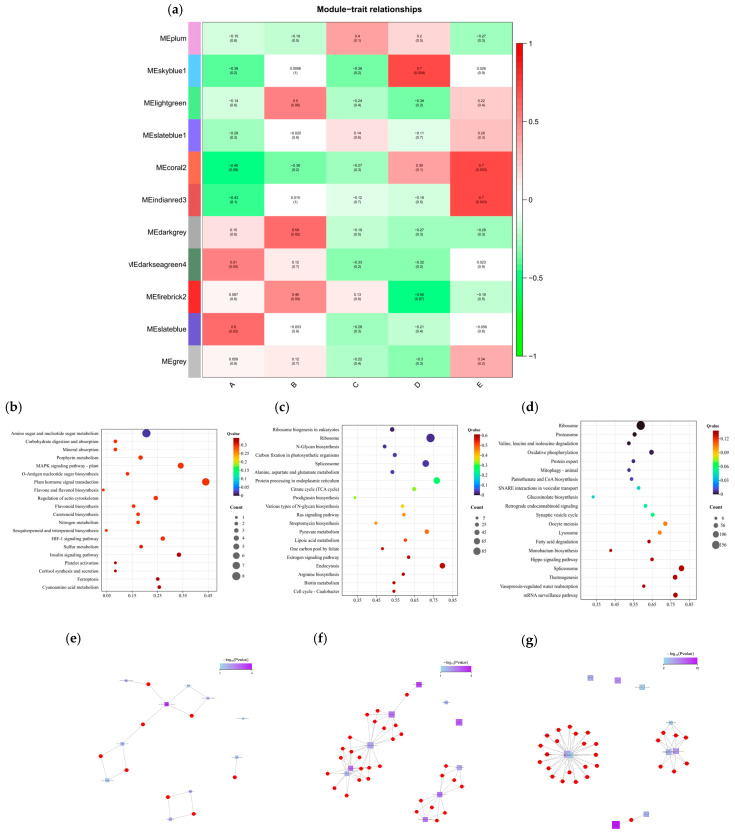
(**a**) Heatmap of gene co-expression network module and feature vector association. Green indicates negative correlation between eigengenes and samples, and red indicates positive correlation. The upper row number is the correlation coefficient, and the lower row number is the *p* value (*p* < 0.05 significant correlation, *p* < 0.01 extremely significant correlation). A: CK, B:50 mmol/L, C:100 mmol/L, D: 150 mmol/L, E: 200 mmol/L. (**b**) Enrichment of KEGG metabolic pathways in MEskyblue1 module. (**c**) Enrichment of KEGG metabolic pathways in MEcoral2 module. (**d**) Enrichment of KEGG metabolic pathways in MEindianred3 module. (**e**) Enrichment network of metabolic pathways in MEskyblue1 module. (**f**) Enrichment network of metabolic pathways in MEcoral2 module. (**g**) Enrichment network of metabolic pathways in MEindianred3 module.

To investigate the DEGs associated with maize’s response to salt stress, a total of 22,298 genes were screened from the transcriptome data for WGCNA. Initially, sample clustering and data correction were performed, followed by the division of genes into 11 modules based on the similarity of their expression patterns (Figure S5). The correlation between modules and samples was calculated, as illustrated in Figure 5a. The results indicated that during salt stress, sample A exhibited a significant positive correlation with the MEdarkseagreen4 and MEslateblue modules, with correlation coefficients of 0.51 and 0.6, respectively. Sample B showed a significant positive correlation with the MElightgreen and MEdarkgrey modules, with correlation coefficients of 0.5 and 0.59, respectively. Additionally, there was an extremely significant positive correlation between sample D and the MEskyblue1 module, with a correlation coefficient of 0.7. Sample E demonstrated a very significant positive correlation with the MEcoral2 and MEindianred3 modules, both having correlation coefficients of 0.7.

In order to further explore the function of the target gene module, KEGG metabolic pathway analysis was performed on the MEskyblue1, MEcoral2, and MEindianred3 modules, and QValue was used for comparative ranking, as shown in Figure 5b–d. Among them, in the MEskyblue1 module, the top metabolic pathways are ‘Amino sugar and nucleotide sugar metabolism’ (Ko00520), ‘Carbohydrate digestion and absorption’ (map04973), ‘Mineral absorption’ (map04978), etc. In the MEcoral2 module, the top metabolic pathways are ‘Ribosome biogenesis in eukaryotes’ (map03008), ‘ribosome’ (Ko03010), ‘N-Glycan biosynthesis’ (map00510), etc. In the MEindianred3 module, the top metabolic pathways are ‘ribosome’ (ko03010), ‘proteasome’ (map03050), ‘valine, leucine and isoleucine degradation’ (map00280), etc.

Enrichment network analysis was then performed, as shown in Figure 5e-g. The results show that the MEskyblue1 module is divided into three concentrated areas. It is mainly focused on ‘Amino sugar and nucleotide sugar metabolism’ (Ko00520), ‘Carbohydrate digestion and absorption’ (map04973), ‘MAPK signaling pathway-plant’ (Ko04016), ‘Flavonoid biosynthesis’ (Ko00941), ‘O-Antigen nucleotide sugar biosynthesis’ (Ko00541), and ‘plant hormone signal transduction’ (Ko04075). The genes involved include *Zm00001d042146*, *Zm00001d021026*, *Zm00001d014842*, *Zm00001d022179*, and *Zm00001d007188*. The MEcoral2 module is divided into two concentrated areas. It is associated with ‘Ribosome biogenesis in eukaryotes’ (map03008), ‘Spliceosome’ (map03040), ‘Protein processing in endoplasmic reticulum’ (map04141), ‘N-Glycan biosynthesis’ (map00510), and ‘Various types of N-glycan biosynthesis’ (map00513), with the genes involved being *Zm00001d047958*, *Zm00001d041119*, *Zm00001d041550*, *Zm00001d028227*, *Zm00001d052020*, etc. The MEindianred3 module has three concentrated regions. It includes ‘oxidative phosphorylation’ (ko00190) and ‘retrograde endocannabinoid signaling’ (map04723). The genes involved are *Zm00001d038508*, *Zm00001d048204*, *Zm00001d038057*, *Zm00001d027493*, *Zm00001d034244*, etc.

#### 2.2.6. Validation of Candidate Gene Expression

**Figure 6:**
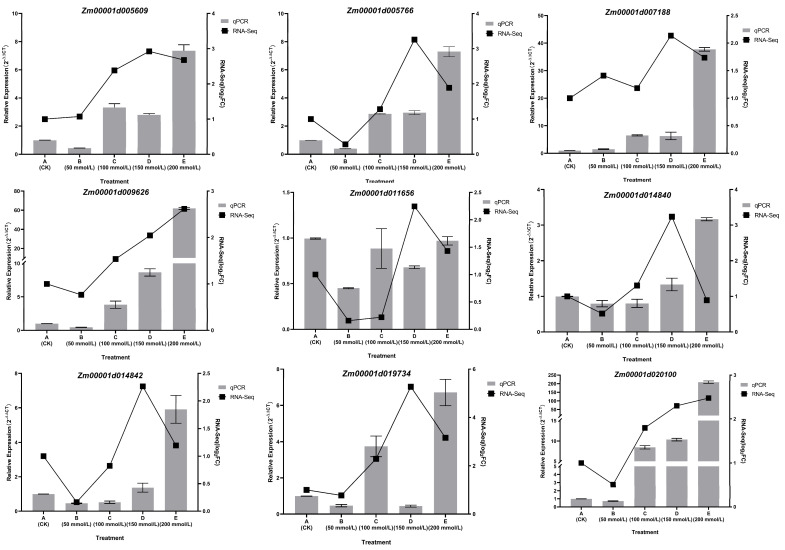
qRT-PCR validation of nine gene expression levels, with a bar chart representing RNA-Seq expression levels and a line chart representing relative expression levels.

To validate the accuracy of the RNA-Seq data, nine genes were randomly selected for qRT-PCR. As depicted in Figure 6, the qRT-PCR results for these genes were in line with the expression patterns observed in the RNA-Seq data. Genes that were significantly up-regulated in the RNA-Seq data also exhibited significant up-regulation in qRT-PCR, and vice versa, thus providing further confirmation of the reliability of the RNA-Seq data.

### 2.3. Metabolomic Analysis of Maize in Response to Salt Stress

**Figure 7:**
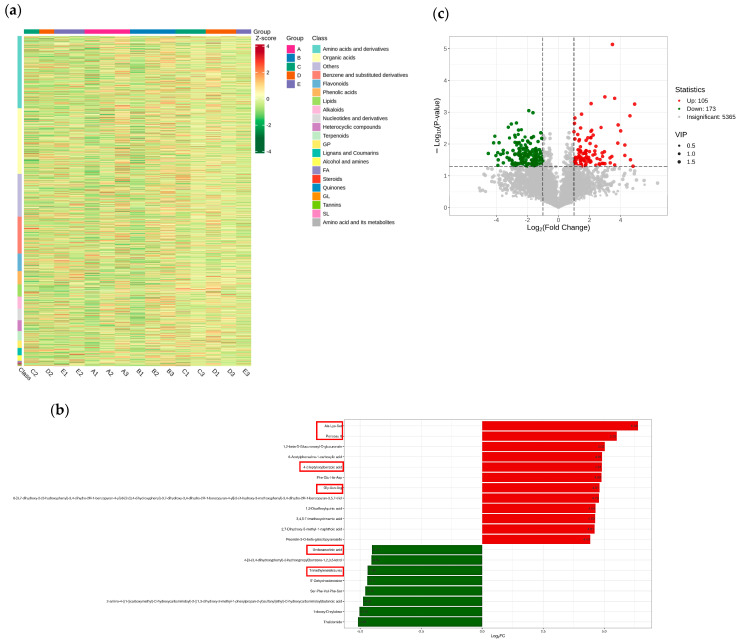
(**a**) Cluster analysis of metabolites. The horizontal direction—the sample name, the vertical direction—the metabolite information. Different colors are the colors filled with different values obtained after standardization of different relative contents: red—high content, green—low content. Class is the first-level classification of substances. The left cluster line is the metabolite cluster line, and the upper cluster line is the sample cluster line. (**b**) Bar chart of DEMs. The abscissa is the log2FC value of DEMs, and the ordinate is the DEMs. Red represents up-regulated expression, and green represents down-regulated expression. The red box is a metabolite repeatedly expressed between groups. (**c**) Volcano map of DEMs. Each point represents a DEM, with red indicating up-regulated, green indicating down-regulated, and gray indicating detected but not significant.

To identify which metabolites undergo changes under varying salinity stress conditions, a metabolomic analysis was conducted on the salt-tolerant material J1285 subjected to salt stress. We employed PCA to evaluate samples. The results showed that minor differences in metabolites were observed among the different treatments, with each treatment demonstrating good reproducibility. A total of 5643 metabolites were detected (Figure S6a). The identified metabolites were subsequently standardized, and cluster analysis was performed based on these data (Figure 7a). The results indicate that varying concentrations of salt stress directly influence the metabolite content in maize.

The top 20 DEMs were obtained based on FC values (Figure 7b and Figure S6b–d). Among these, Ala-lys-Ser, Albanin A, Gly-Asn-Arg, etc., repeatedly appear in each group, suggesting they are the corresponding metabolites of corn in response to salt stress. To further analyze the DEMs of maize in response to salt stress, OPLS-DA was employed for the qualitative and quantitative analysis of all detected metabolites, as shown in Figure 7c and Figure S6e–g. With the increase in salt concentration, the number of significant DEMs also increased, indicating that these metabolites are closely related to salt stress.

**Figure 8:**
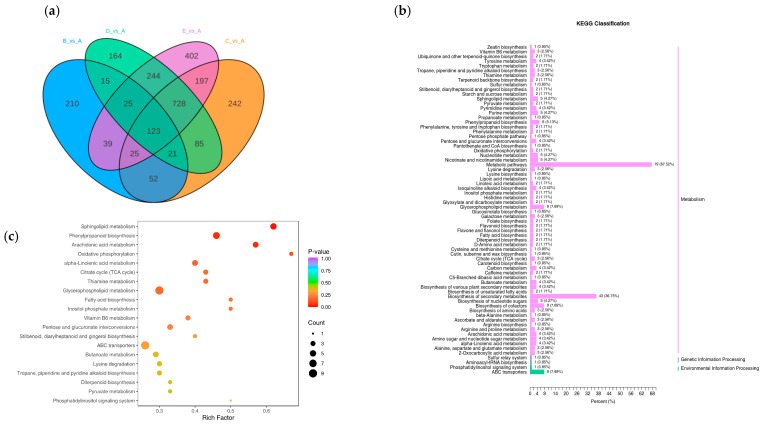
(**a**) Venn diagram of DEMs. A: CK, B:50 mmol/L, C:100 mmol/L, D: 150 mmol/L, E: 200 mmol/L. (**b**) KEGG classification of DEMs. (**c**) Enrichment analysis of DEMs.

Therefore, the commonly enriched DEMs among the groups were identified using a Venn diagram (Figure 8). A total of 123 common DEMs were obtained from pairwise comparisons between the groups, and statistical analysis was conducted (Table S6 and Figure S6h).

#### 2.3.1. GO and KEGG Enrichment Analysis of DEMs Involved in Salt Tolerance Response

Firstly, DEMs were annotated and classified into pathways, revealing that the primary metabolic pathways they participate in are ‘Metabolic pathways’ and ‘Biosynthesis of secondary metabolites.’ With the increase in salt stress concentration, secondary metabolism gradually increases, and the cellular processes section is partially replaced (Figure 8b and Figure S7a–c). Subsequent enrichment analysis of DEMs revealed that DEMs were primarily enriched in ‘2-Oxocarboxylic acid metabolism’ (Ko01210), ‘Glycerophospholipid metabolism’ (Ko00564), ‘Tyrosine metabolism’ (Ko00350), ‘Sphingolipid metabolism’ (Ko00600), ‘Phenylpropanoid biosynthesis’ (Ko00940), ‘Biosynthesis of nucleotide sugars’ (Ko01250), and others (Figure 8c and Figure S7d–f).

#### 2.3.2. Metabolomic WGCNA

To understand the differential expression metabolites related to J1285 response to salt stress, a total of 5643 metabolites were selected from the metabolome data for WGCNA (Figure S8a,b). Initially, sample clustering and data correction were performed, after which the metabolites were categorized into 24 modules based on the similarity of their expression patterns.

**Figure 9:**
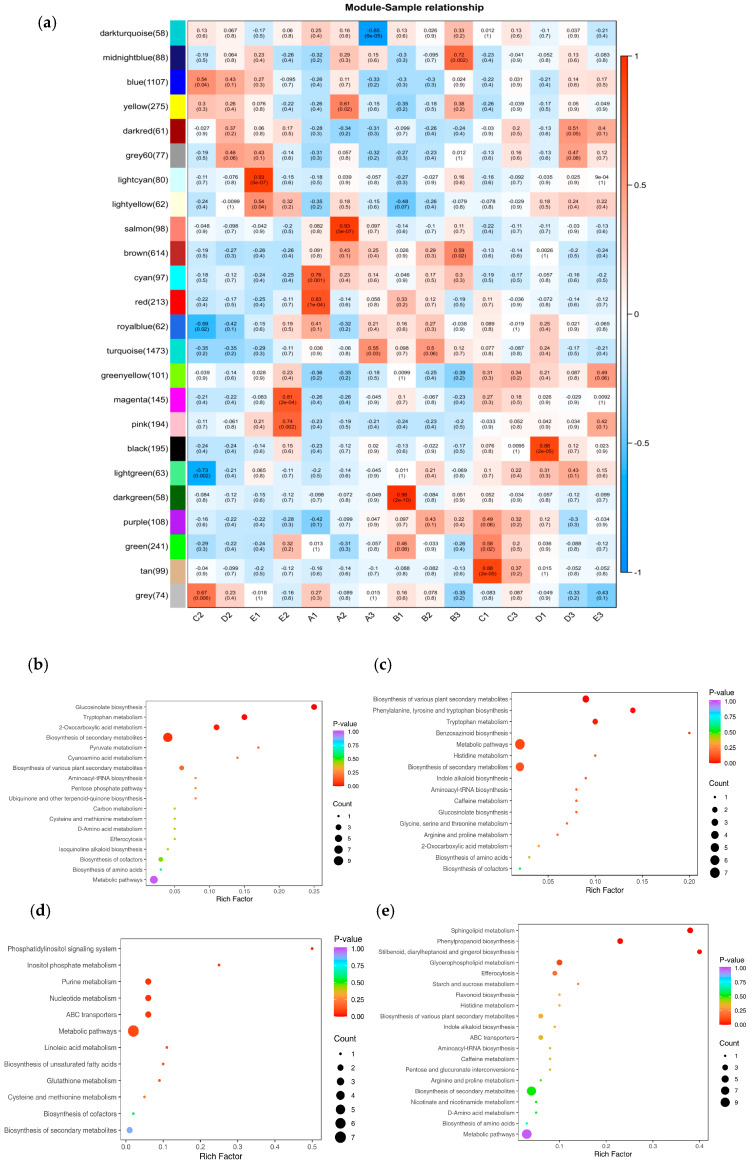
(**a**) Heatmap of gene co-expression network module and feature vector association. Blue indicates negative correlation between eigengenes and samples, and red indicates positive correlation. The upper row number is the correlation coefficient, and the lower row number is the *p* value, *p* < 0.05 significant correlation, *p* < 0.01 extremely significant correlation. A: CK, B:50 mmol/L, C:100 mmol/L, D: 150 mmol/L, E: 200 mmol/L. (**b**) Enrichment of KEGG metabolic pathways in light cyan module. (**c**) Enrichment of KEGG metabolic pathways in light yellow module. (**d**) Enrichment of KEGG metabolic pathways in magenta module. (**e**) Enrichment of KEGG metabolic pathways in pink module.

We calculated the correlation between the modules and the samples (Figure 9a). The results indicate that during salt stress, the light cyan module showed a highly significant positive correlation with E1, and the light yellow module exhibited a significant positive correlation with E1, with correlation coefficients of 0.93 and 0.54, respectively. The magenta and pink modules are significantly positively correlated with E2, with correlation coefficients of 0.81 and 0.74, respectively.

To further explore the functional roles of the target gene modules, KEGG pathway analysis was conducted on the light cyan, light-yellow, magenta, and pink modules, with screening based on *p*-Value. The results are shown in Figure 9b–e. There are some common pathways in these extracted modules, including, ‘Tryptophan metabolism’ (map00380), ‘2-Oxocarboxylic acid metabolism’ (map01210), ‘Biosynthesis of secondary metabolites’ (map01110), and ‘Metabolic pathways’ (map01100).

### 2.4. Joint Analysis of Omics of Maize in Response to Salt Stress

**Figure 10:**
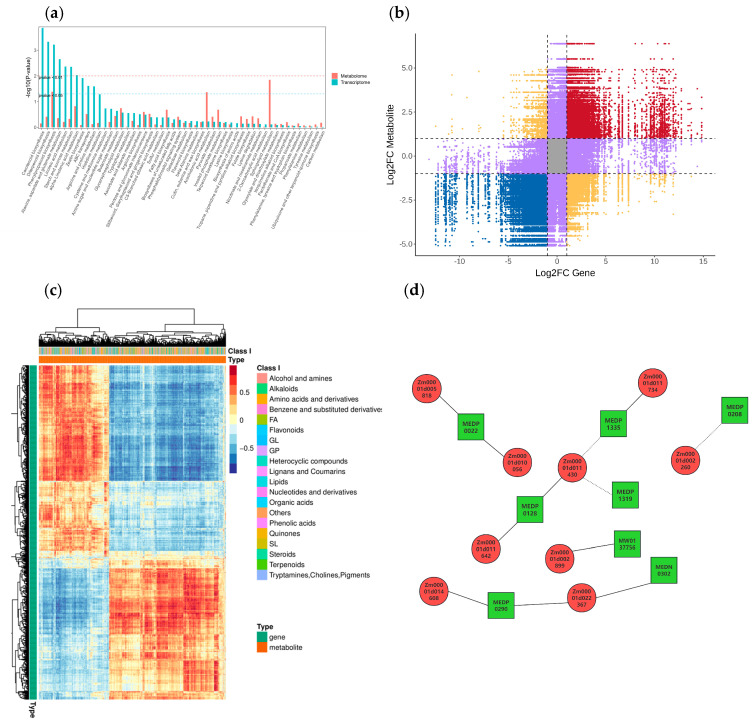
(**a**) KEGG co-enrichment analysis. (**b**) Nine quadrants analysis. (**c**) Cluster heat map analysis of correlation. Each row represents a gene, and each column represents a metabolite; red indicates positive correlation between genes and metabolites, and blue indicates negative correlation between genes and metabolites. (**d**) Network analysis of correlation. Green squares represents metabolites, red circles represents genes, solid lines represents positive correlation, and dashed lines represents negative correlation.

According to the results of KEGG enrichment analysis of DEGs and DEMs, the common KEGG enrichment analysis of the two groups are shown in Figure 10a and Figure S9a–c, where the pathway with *p*-values <0.05 for DEGs and DEMs is ‘Phenylpropanoid biosynthesis’ (map00940).

Based on data analysis and inference, it is known that although the pathways involved by these metabolites differ and their functions vary, they are predominantly related to metabolism, including metabolic pathways, carbohydrate metabolism, lipid metabolism, amino acid metabolism, biosynthesis of other secondary metabolites, etc.

We analyzed the FC of substances within each differential group using a nine-quadrant diagram, with black dashed lines dividing it into 1–9 quadrants from left to right and top to bottom, as shown in Figure 10b and Figure S9d–f. The key areas to focus on are quadrants 3 and 7, which represent consistent differential expression patterns between genes and metabolites, indicating a positive correlation where the expression changes of metabolites may be positively regulated by genes. Relevant genes and metabolites can be mined from these findings.

We selected all the correlation calculation results of DEGs and DEMs to plot the correlation clustering heatmap (Figure 10c and Figure S9g–i). With the increase in salt stress concentration, the similarity in expression patterns between genes and metabolites also increases. Relevant genes and metabolites can be screened and mined from these groups with high expression pattern similarity.

Subsequently, the correlation between metabolites and genes was analyzed through network analysis, as shown in Figure 10d. The analysis revealed positive correlations between *Zm00001d010056*, *Zm00001d005818* and MEDP0022, between *Zm00001d011734* and MEDP1335, between *Zm00001d011430*, *Zm00001d011642* and MEDP0128, between *Zm00001d002899* and MW0137756, and between *Zm00001d014608*, *Zm00001d022367* and MEDP0290, MEDN0302. The analysis also revealed negative correlations between *Zm00001d011430* and MEDP1335, MEDP1319 and between *Zm00001d002260* and MEDP0208. These metabolites and genes are relatively dispersed, indicating that the way maize responds to salt stress is complex and completed by many aspects.

## 3. Discussion

### 3.1. Effects of Salt Stress on Phenotypic and Physiological of Maize

Salt stress can induce chlorosis in plant leaves, reduce biomass, slow plant growth, decrease spike number, and lower thousand-grain weight, ultimately impacting the harvest index and grain yield [33]. Maize exhibits a certain level of sensitivity to salt stress; therefore, we selected various salt treatment concentrations as variables for this study and analysis. The findings indicated that salt stress impeded seedling growth to a significant degree. Salt stress caused the phenotypic traits of J1285 to initially increase and then decrease, as demonstrated by Lu et al. [34]. The growth of maize was promoted with a 50 mmol/L salt concentration treatment, while a 100 mmol/L treatment inhibited growth. Other studies have indicated that low and medium salt concentrations (50 and 100 mmol/L) can enhance the development of Bassia dasyphylla and Halogeton arachnoideus, whereas higher salt concentrations hinder their growth [35]. This phenomenon may be attributed to plants adapting to their environment through self-regulation under salt stress. The fluctuation in fresh and dry weights, which follows an increasing-decreasing trend in salt treatments, may also be due to this same reason, varying from low to high salt concentration (50–200 mmol/L).

The process of stress persistence involves the ability of plants to resist and adapt to stress while adjusting their physiological processes. Superoxide dismutase (SOD), peroxidase (POD), and catalase (CAT) can be considered components of a protective system that helps balance reactive oxygen species (ROS) in plants, thereby enhancing their stress resistance. In this study, Figure 1 illustrates how plants adapt when exposed to salt stress. The activity of antioxidant enzymes, such as SOD and CAT, in leaves subjected to salt treatment initially decreases and then increases with the intensity of the stress. This fluctuation in enzyme activity suggests that plants can self-regulate to cope with stress without reaching levels that trigger excessive production of free radicals. However, when the salt concentration reaches 50 mmol/L, the free radicals produced by the plants begin to serve as substrates that induce or activate antioxidant enzyme activity, leading to an increase in SOD activity. At a salt concentration of 150 mmol/L, the activity of superoxide dismutase (SOD) shows a significant difference compared to the control group. When the salt concentration reaches 150 mmol/L, SOD activity peaks and then begins to decline, suggesting that 150 mmol/L may represent a critical threshold for SOD activity. Additionally, at a salt concentration of 50 mmol/L, catalase (CAT) activity significantly differs from that of the control group. These results indicate that a salt concentration of 50 mmol/L serves as the initial point of impact on the antioxidant system of maize, while a concentration of 150 mmol/L may represent a critical stress threshold. This suggests that the production and scavenging of reactive oxygen species have severely disrupted the dynamic balance in maize. Under salt stress, the production of reactive oxygen species exceeds the scavenging capacity of the system, leading to lipid peroxidation of the cell membrane and the generation of cytotoxic substances.

### 3.2. Effects of Salt Stress on Maize Omics Analysis

Transcriptome analysis provides valuable insights into a plant’s response to salt stress by examining the overall patterns of gene expression [36]. Previous studies on the transcriptomes of salt-tolerant maize have primarily focused on assessing expression levels at both the onset and conclusion of salt stress [37,38]. In this study, we performed transcriptome analysis on seedlings subjected to five different concentrations of salt treatment to explore the similarities and differences in the mechanisms of salt tolerance. Recent studies have demonstrated that the metabolic pathways and their regulatory genes in rice can be identified through transcriptomic and metabolomic analyses [39]. These studies have also revealed the potential reaction mechanisms of quinoa under heat stress [40] and the effects of selenium fertilizer on the fruit of *Lycium barbarum* [41]. Future research may involve additional analyses and studies that integrate transcriptomics and metabolomics to enhance the development of salt-tolerant maize varieties.

Plant hormones are small molecules that regulate growth and development in plants, acting as signaling molecules within the organism. They play a crucial role in transmitting biological signals and controlling various growth and developmental processes. Numerous studies have demonstrated the involvement of these hormones in mediating plant tolerance to stress conditions [42,43,44]. Additionally, the KEGG pathway enriches the synthesis and metabolism pathways of various plant hormones [45,46,47].

Tryptophan serves as the precursor for indole-3-acetic acid (IAA), and alterations in its synthesis directly influence IAA formation [48]. Under salt stress, plant growth-promoting rhizobacteria (PGPR) activate the ethylene signaling pathway by inducing methionine accumulation, thereby enhancing the salt tolerance of plants [49]. The carotenoid pathway not only serves as a precursor source for abscisic acid (ABA) synthesis but also indirectly influences ABA levels by regulating carotenoid accumulation [50]. In sesame, the expression changes of the lipoxygenase gene (Lox) under drought and salt stress are closely associated with the biosynthesis and signal transduction of jasmonic acid [51]. This study identifies significant differential expression of genes involved in stress hormone signaling, particularly within the ABA signaling pathways: plants exhibit the up-regulation of genes related to ABA biosynthesis pathway. In this study, differentially expressed genes (DEGs) were found to be significantly enriched in the ABA signaling pathway, revealing a connection between the ABA signaling pathway and the MAPK signaling pathway.

Salt stress was observed to induce changes in the expression levels of PYR/PYL in the ABA signaling pathway, leading to the alleviation of SnRK2 inhibition by PP2C and subsequent activation of the MAPK signaling pathway. Previous studies have shown that liquiritigenin accumulation responds to salt stress via the ABA signaling pathway, as demonstrated in transcriptome analysis [52]. Additionally, research has demonstrated that *OsbZIP62* plays a role in ABA signaling response, positively regulating drought and salt stress tolerance in rice [53]. *OsPP2C68*, as a negative regulator of the ABA signaling pathway, exhibits increased sensitivity to salt stress in its knockout mutants [54]. Additionally, *FYVE1* negatively regulates the ABA signaling pathway by degrading the ABA receptors PYR1 and PYL4, thereby reducing the salt tolerance of plants [55].

The MAPK pathway, which consists of a series of intracellular signaling factors, transmits external signals to the cell through a three-tiered protein kinase cascade involving MAPKKKs, MAPKKs, and MAPKs [56]. The activation of MAPK and its downstream genes can significantly influence plant stress resistance [57]. Research has demonstrated that plants respond to salt stress by modulating protein kinases, including MAPKs [58]. Studies have underscored the critical role of SlMAPK3 in the response of tomato plants to salt stress [59]. In transgenic poplar, the overexpression of PeMKK2a markedly increased the activities of superoxide dismutase (SOD), catalase (CAT), and peroxidase (POD), thereby enhancing the salt tolerance of the plants [60]. Additionally, exogenous application of NaHS improved the salt tolerance of tomato plants by up-regulating the expression of MAPK3, MAPK4, MAPK6, and MAPK9 [61].

KEGG analysis also revealed enrichment of pathways such as ‘ribosome’ (ko03010), ‘phenylpropanoid biosynthesis’ (ko00940), ‘arginine and proline metabolism’ (ko00330), ‘Amino sugar and nucleotide sugar metabolism’ (ko00520), and ‘Neurotrophin signaling pathway’ (ko04722) in differentially expressed genes (DEGs). Notably, phenylpropanoid biosynthesis showed both up-regulated and down-regulated DEGs. In perennial ryegrass, the salt-tolerant genotype exhibited elevated levels of phenylpropanoids, flavonoids, and anthocyanins, accompanied by a significant up-regulation of related gene expression [62]. Similarly, in other plants such as tomato [63], mulberry [64], and amorpha [65], both the genes and metabolites associated with the phenylpropanoid biosynthetic pathway demonstrated notable alterations in response to salt stress.

According to the results of previous studies and the results of phenotypic, physiological and transcriptome analysis in this study, we speculated that the biosynthesis and signal transduction of plant hormones (especially ABA signaling pathway and MAPK signaling pathway) were stimulated to a certain extent during the development of maize seedlings under salt stress. The expression of hormone signals in response to stressful environments and their role in mediating salt tolerance are not independent processes. Instead, a range of hormone signals and their components interact in the regulation of abiotic stress. Therefore, it is essential to thoroughly investigate the intricate and extensive regulatory network involved in the response to abiotic stress.

Transcription factors play a crucial role in plant responses to salt stress, with *WRKY*, *TIFY*, *bZIP*, and *bHLH* transcription factors being particularly important for maize growth and recovery under such conditions. Previous studies have shown that various transcription factors, including *WRKY*, *bZIP*, and *bHLH*, are activated by salt stress and are essential for plants to cope with drought and chilling stress [66,67,68]. Transcriptome analysis of *Zoysia japonica Steud* indicated that the regulation of salt stress in this plant species is closely associated with the auxin signal transduction family, ABA signal transduction family, WRKY transcription factor family, and bHLH transcription factor family [69]. Notably, bZIP transcription factors are particularly crucial for responding to abiotic stresses such as salt, drought, cold, osmotic stress, mechanical damage, and ABA signaling in plants [70]. KEGG analysis showed that DEGs encoding transcription factors under salt stress were significantly enriched in the categories of “plant hormone signal transduction” and “MAPK signaling pathway”. In this study, several transcription factors, including WRKYs (*Zm00001d043025*, *Zm00001d038023*, *Zm00001d038451* and *Zm00001d010399*), TIFYs (*Zm00001d048263*, *Zm00001d027899*, *Zm00001d027901* and *Zm00001d033050*), bZIPs (*Zm00001d024160*, *Zm00001d046751*, *Zm00001d042779* and *Zm00001d012296*), and bHLHs (*Zm00001d030028* and *Zm00001d047017*), were uniquely and significantly expressed in maize in response to salt stress. This suggests that these transcription factors may play a role in maize’s salt tolerance by modulating relevant pathways.

In summary, the different salt tolerance levels of maize inbred line J1285 under salt stress were screened, and transcriptome analysis was carried out to identify the genes related to salt tolerance of maize seedlings. The expression of differentially expressed genes (DEGs) in salt-tolerant seedlings was primarily associated with the abscisic acid (ABA) and mitogen-activated protein kinase (MAPK) signaling pathways, which were significantly induced under salt treatment. This finding is partially consistent with the physiological results regarding the salt tolerance of the seedlings. The results showed that the increase in ABA and MAPK signaling pathway-related genes in maize could effectively compensate the seedling growth inhibition caused by salt stress. From this, it can be inferred that many hormonal signals and their components are intertwined in the regulation of abiotic stress. Therefore, it is essential to conduct in-depth research into the response mechanisms of this extensive and complex regulatory network to abiotic stress.

Metabolomics can play a significant role in studying plant responses to abiotic stress [71]. Abdel-Farid et al. [72] discovered through metabolomics research that cucumbers and tomatoes exhibit different salt stress tolerance strategies, likely due to differences in their metabolic levels. In this experiment, secondary metabolites, tryptophan metabolism, 2-oxocarboxylic acid metabolism, and metabolic pathways were enriched. Exogenous melatonin treatment significantly enhanced the salt tolerance of bitter melon under salt stress by regulating the expression of antioxidant system- and secondary metabolism-related genes such as *MAP30* and *PAL* [73]. Furthermore, under salt stress, the secondary metabolic pathways in sweet sorghum (such as hormone signaling and stress response) were significantly activated, further supporting the critical role of secondary metabolism in salt tolerance [74]. The 2-oxocarboxylic acid metabolism is closely related to amino acid metabolism and plant hormone biosynthesis. Under salt stress, the metabolism of glycine, serine, and threonine in plants is significantly enhanced [75]. These metabolic pathways are cross-regulated with 2-oxocarboxylic acid metabolism. The integrated analysis of transcriptomics and metabolomics can more comprehensively reveal the molecular mechanisms of salt tolerance in maize. Through the combination of transcriptomic and metabolomic analyses, researchers have identified a series of key genes associated with salt tolerance in maize [76]. Furthermore, the differentially expressed genes under salt stress are closely related to changes in metabolites. Using weighted gene co-expression network analysis (WGCNA), several hub genes related to salt response have been identified (such as the ABC transporter family and calcium transport ATPase), which are significantly correlated with the changes in metabolites (such as proline and glutamic acid) [76]. Ma et al. [77] identified two hub genes, *GRMZM2G075104* and *GRMZM2G333183*, related to salt tolerance through GWAS and WGCNA and confirmed their impact on salt tolerance during the seedling stage of maize. Additionally, it was found that the gene *Zm00001d023379*, identified through WGCNA, can regulate root angle and the number of shoot-borne roots [78]. This clearly demonstrates that WGCNA, as a robust systems biology tool, can offer new insights into the mechanisms of salt tolerance in maize.

This study conducted a combined transcriptomic and metabolomic analysis on seedlings treated with five different concentrations of salt stress to deeply investigate the salt tolerance mechanisms in maize. These omics data provide a wealth of informational resources for deciphering the mechanisms of stress resistance in maize. Future research can build upon these findings to develop more efficient breeding strategies and cultivate new maize varieties with improved salt tolerance, as well as provide strong genetic resource support and make important contributions to the sustainable development of global agriculture and food security.

## 4. Materials and Methods

### 4.1. Plant Materials and Experimental Treatment

The plant material used in this study was J1285 (Lancaster), which was provided by Maize Breeding Innovation Team in Jilin Agricultural University. All plants (either cultivated or wild), including the collection of plant material, complied with relevant institutional, national and international guidelines and legislation. The seeds were germinated on soaked filter paper for 2 days at 25 °C and saturated soil moisture conditions, then transferred to plastic flowerpots (height 10 cm, outer diameter 14.8 cm) containing 1.5 kg of nutrient soil (mixed with vermiculite, peat, vermicompost, etc., pH value of 6–6.5), where they were grown for 2 weeks. The seedlings at the three-leaf and one-heart stage were cultivated for 8 days and treated every 2 days with salt (100 mL) before harvesting, and leaves were quick-frozen in liquid nitrogen for RNA extraction. Salt concentration gradients were set to 0, 50, 100, 150, and 200 mmol/L. Seedlings grown under distilled water were used as an untreated control (CK). The experimental data were determined using three independent biological repeats with ten plants per replicate, and three technical replicates by completely random design. The leaves were sent to Sangon Biotech (Shanghai) Co., Ltd. (Shanghai, China) for sequencing.

### 4.2. Measurements of Phenotypic and Determination of Antioxidant Enzymes

The determination methods of agronomic traits such as leaf length and leaf width referred to this laboratory. Fresh maize seedlings were immediately measured for fresh weight after harvest, and dry weight was measured by oven-drying the harvested fresh samples for 30 min at 105 °C until a constant mass was reached at 80 °C. To analyze the physiological traits of the shoot of maize seedlings with and without saline stress treatment, the following physiological parameters were measured according to the manufacturer’s protocols of their corresponding assay kits (Beijing Solarbio Science & Technology Co., Ltd., Beijing, China): the activity of superoxide dismutase (SOD), peroxidase (POD), and catalase (CAT). Leaf samples (0.1 g frozen weight) were homogenized in 1 mL of buffer to assay the activity of antioxidant enzymes including SOD, POD, and CAT. After centrifugation at 8000× *g* for 10 min at 4 °C, the supernatant was subsequently used for measurement of antioxidant enzyme activity.

### 4.3. Transcriptome Profiling

Constructing mRNA libraries and sequencing were carried out on the Illumina Hiseq platform. The quality of the sequencing data from the samples was visually assessed using FastQC. Raw data were filtered to remove adapter sequences, poly-N reads, and low-quality reads, resulting in clean reads. Quality parameters of the clean data were calculated, including Q20, Q30, and GC content. The quality-controlled sequencing sequences were aligned to the default reference genome using HISAT2, and the alignment results were statistically analyzed using RSeQC. Subsequently, the FPKM values for each gene were calculated based on gene length and the number of mapped reads. To gain deeper insights into the phenotypic changes, enrichment analysis of differentially expressed genes (DEGs) was conducted using the TopGO R package 2.24.0 and the clusterProfiler R package for GO (https://geneontology.org/, accessed on 28 June 2023) enrichment analysis and KEGG (https://www.kegg.jp/, accessed on 28 June 2023) for pathway analysis, with an enrichment *p*-value < 0.05.

Weight Gene Co-expression Network Analysis (WGCNA) of all differentially expressed genes (DEGs) was constructed using the WGCNA R package [79]. The gene co-expression correlation matrix was established based on the scale-free network distribution, with the definition of its adjacency function. The topological overlap measure (TOM) served as the foundation for hierarchical cluster analysis (HCA), which was completed using the Pearson correlation coefficient. Genes with a mean TPM < 0.5 were excluded as part of the filtering criteria. All genes were grouped into clusters. Gene significance (GS) and module membership (MM) were calculated, and the modules were associated with phenotype data. The information of the corresponding module genes was extracted for further analysis.

### 4.4. qRT-PCR Analysis

Total RNA was extracted from maize leaves using TRIzol reagent (Invitrogen, Shanghai, China) according to the manufacturer’s instruction. Total RNA (1 μg) was used to reverse transcribe into complementary DNA (cDNA) with ReverTra Ace, qPCR RT Kit (TOYOBO, Tokyo, Japan) following the manufacture’s instruction. qRT-PCR was performed using a SYBR Mixture system (TOYOBO, Tokyo, Japan) on a QuantStudio 3 instrument (Thermo, Waltham, MA, USA). A maize Actin gene, *ZmTub* (*GRMZM2G066191*), was used as an internal control to normalize the data. The relative expression level of target genes was calculated using the 2^−∆∆CT^ method [80]. The experimental data were determined using three biological and three independent technical replicates, then the significance analysis was performed using Student’s *t*-test (* *p* < 0.05, ** *p* < 0.01). Bars indicate standard error of the mean. The primers used for assays are listed in Table S1.

### 4.5. Metabolome Profiling

The same set of samples used for RNA-seq were also subjected to metabolome analyses. Each sample included 6 biological replicates. The non-target metabolome analysis was performed by Sangon Biotech (Shanghai) Co., Ltd. The extraction, separation, identification, and data processing of metabolites were conducted using the LC-30A ultra-high performance liquid chromatography system (Shimadzu, Kyoto, Japan) and the Triple TOF 6600+ mass spectrometer (SCIEX, Foster City, CA, USA). Mass spectrometry data were acquired in both negative ion mode (ESI^−^) and positive ion mode (ESI^+^) to enhance metabolite coverage. The XCMS program was employed for processing the LC-MS/MS data, which primarily included peak extraction, peak alignment, and compound identification. Metabolites were identified by searching the self-built database of the Shanghai Shenggong Company laboratory, integrating public databases, predictive databases, and the metDNA method. A threshold of VIP > 1, FC ≥ 2, or FC ≤ 0.5 was applied. Analysis was performed using the Metabo Analyst R package’s OPLSR.Anal function in R software. The KEGG database was utilized to explore the functional associations of various metabolites and their potential involvement in metabolic pathways for functional annotation and enrichment analysis.

The WGCNA package in R software was used to perform WGCNA analysis on the differentially expressed metabolites (DEMs), setting a soft threshold of 18, a minimum gene number of 50 within modules, and a similarity module merging threshold of 0.25.

### 4.6. Joint Analysis of Omics

We integrated the DEMs and DEGs, then conducted a comprehensive joint analysis of DEMs and DEGs within the same groups. This analysis encompassed functional analysis, expression trend analysis, expression level correlation analysis, and co-expression clustering analysis, ultimately leading to the identification of the most relevant metabolic pathways, genes, and metabolites. The correlation analysis was performed using the ‘cor’ function in R to compute the Pearson correlation coefficient between genes and metabolites, with the screening criteria established as a Pearson correlation coefficient > 0.8 and *p*-value < 0.05.

### 4.7. Statistical Analyses

Microsoft Excel and SPSS 25.0 were used for the arrangement of data and analysis of calculations. All data were analyzed through a one-way analysis of variance (ANOVA; *p* = 0.05). Significant differences between the multiple treatment groups were evaluated using Student’s *t*-test (*p* < 0.05). All heatmaps of expression levels in the study were carried out using GraphPad Prism 8 and R package. All data are presented as the mean of three replicates.

## 5. Conclusions

This study systematically elucidates the molecular mechanisms underlying maize’s response to salt stress through phenotypic observation, physiological index measurement, and a combined analysis of transcriptomics and metabolomics. Under salt stress, maize seedlings exhibit typical stress response phenotypes; low concentrations (50 mmol/L) of salt can potentially stimulate compensatory growth, while high concentrations (150 mmol/L) lead to growth inhibition. Additionally, redox processes, metabolic regulation, and plant hormone signal transduction—particularly within the MAPK signaling pathway—may play a central role in salt stress signal transduction. These genes mediate maize’s adaptive response to salt stress by regulating cellular localization, biological processes, and responses to stimuli. Maize also responds to salt stress through the regulation of organic acid synthesis, membrane lipid metabolism, and the accumulation of secondary metabolites. DEGs and DEMs are co-enriched in 24 pathways, encompassing various areas such as amino acid, lipid, flavonoid, and nucleotide metabolism, thereby forming a complex regulatory network. Collectively, these pathways mediate maize’s salt tolerance by synergistically regulating antioxidant enzyme activity, osmotic substance synthesis, and cellular signal transduction. In summary, maize’s salt tolerance results from the interplay between phenotypic adaptation, gene expression regulation, and metabolism, involving multidimensional mechanisms such as the activation of the antioxidant system, accumulation of osmotic regulatory substances, remodeling of membrane lipid metabolism, and transduction of signaling pathways. This study provides new insights into the molecular basis of maize salt tolerance, with relevant pathways and candidate genes serving as important targets for the improvement of salt-tolerant varieties.

## Data Availability

The raw data of the RNA-seq from the next generation sequence under study were deposited into the National Center for Biotechnology Information (NCBI) Sequence Reads Archive (SRA) database under accession number PRJNA1072161. The metabolomic raw data reported in this paper have been deposited in the EMBL-EBI MetaboLights database with the identifier MTBLS10517, and are accessible at https://www.ebi.ac.uk/metabolights/editor/study/MTBLS10517, accessed on 24 June 2024.

## Supplementary Materials

The following supporting information can be downloaded at: https://www.mdpi.com/article/10.3390/plants14132031/s1, Figure S1: PCA of transcriptome data from with and without saline stress; Figure S2: Gene Function Classification (GO); Figure S3: Bubble plots of the GO items in the GO enrichment analysis of DEGs; Figure S4: Bubble plots of the KEGG items in the GO enrichment analysis of DEGs; Figure S5: WGCNA Cluster dendrogram and Heatmap of the identified modules (Gene); Figure S6: Metabolomic analysis; Figure S7: KEGG classification and enrichment; Figure S8: WGCNA Cluster dendrogram and Heatmap of the identified modules (Meta); Figure S9: Common analysis; Table S1: Primers used in qRT-PCR under study; Table S2: Transcriptome data; Table S3: Significant DEGs during the four salt treatment stages compared with the control; Table S4: Significantly enriched DEGs with the plant hormone signal transduction in maize J1285; Table S5: Significantly enriched DEGs with the MAPK signaling pathway in maize J1285; Table S6: The TFs responses to salt stress in maize J1285.

## Abbreviations

The following abbreviations are used in this manuscript:

BP	Biological process
CC	Cellular component
DEG	Differential expression gene
DEM	Differential expression metabolite
DREG	Down-regulated Expression Gene
DREM	Down-regulated Expression Metabolite
FC	Fold change
GO	Gene Ontology
KEGG	Kyoto Encyclopedia of Genes and Genomes
MF	Molecular function
PCA	Principal component analysis
qRT-PCR	Quantitative real-time polymerase chain reaction
UREG	Up-regulated Expression Gene
UREM	Up-regulated Expression Metabolite
WGCNA	Weighted gene co-expression network analysis
