# Supplementary figures and images for: Molecular Mechanisms Underlying Salt Tolerance in Maize: A Combined Transcriptome and Metabolome Analysis
Source: Plants (Basel). 2025 Jul 2;14(13):2031. doi: 10.3390/plants14132031 (PMC12251963; doi:10.3390/plants14132031)

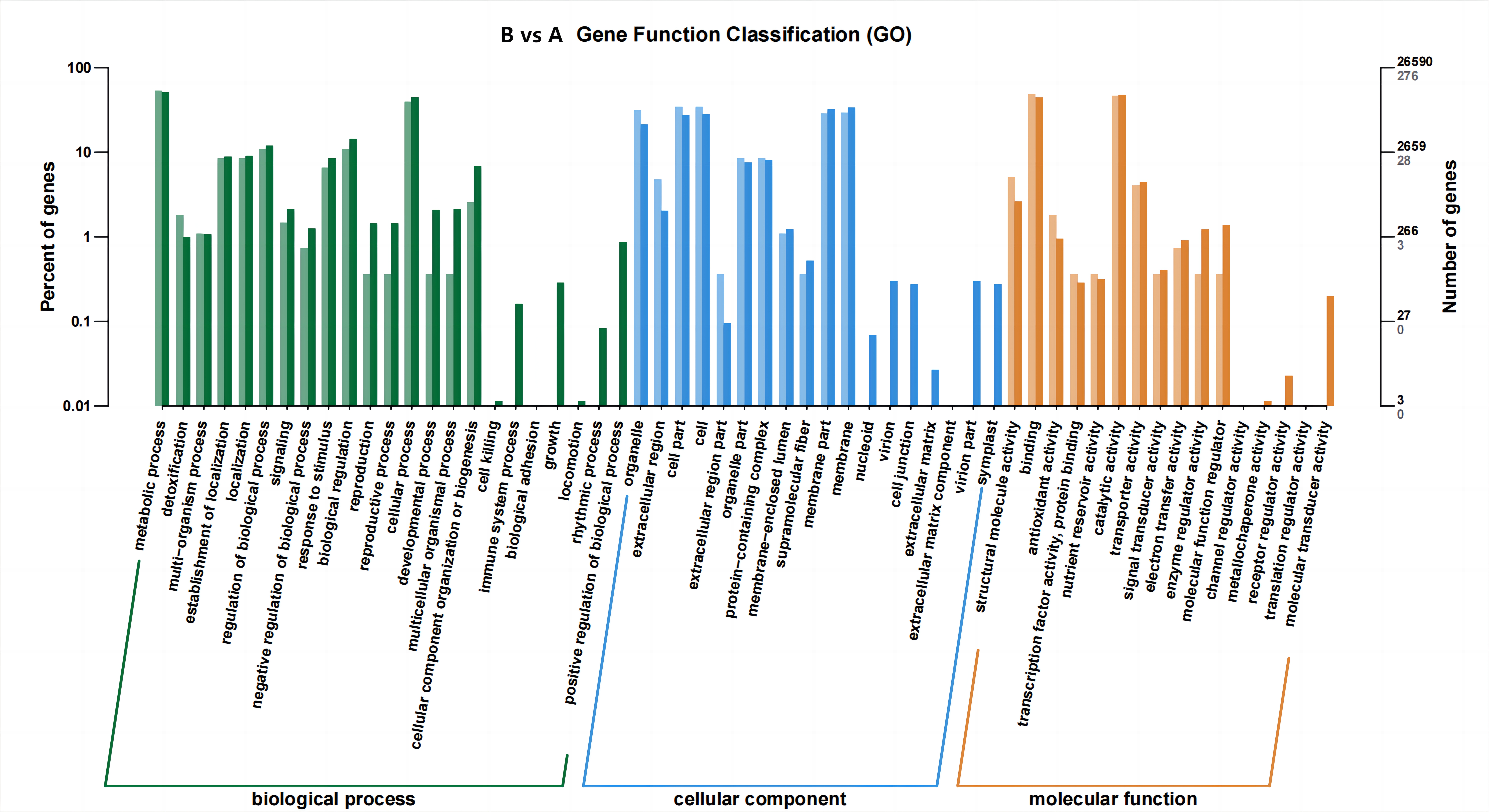

Supplement: Supplementary file 1 [file plants-14-02031-s001.zip › Supplementary Files/Supplementary Pictures/Figure S2-Gene Function Classification (GO)/Figure S2a-B_vs_A.sign_GO_categorie_00.jpg]

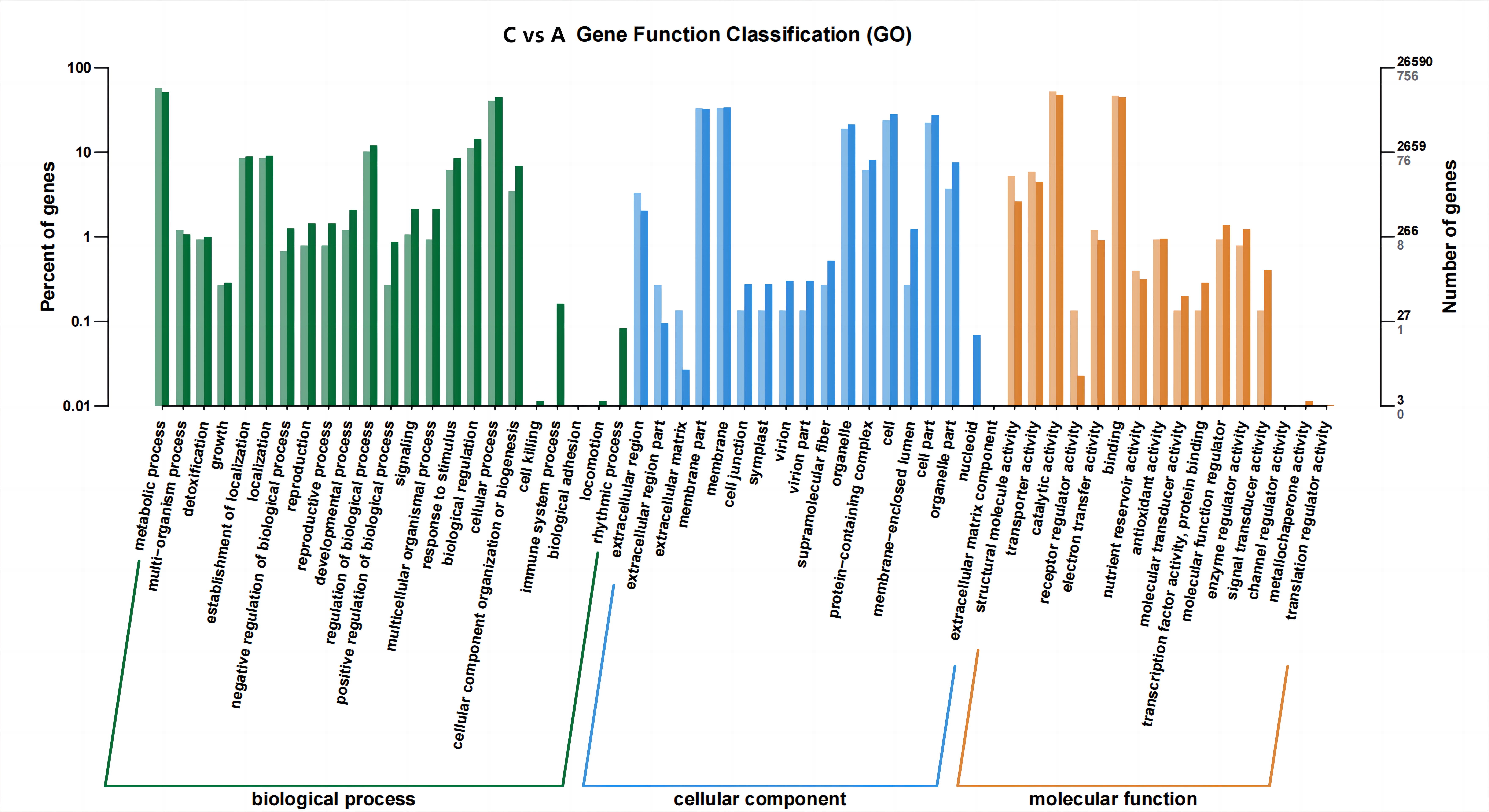

Supplement: Supplementary file 1 [file plants-14-02031-s001.zip › Supplementary Files/Supplementary Pictures/Figure S2-Gene Function Classification (GO)/Figure S2b-C_vs_A.sign_GO_categorie_00.jpg]

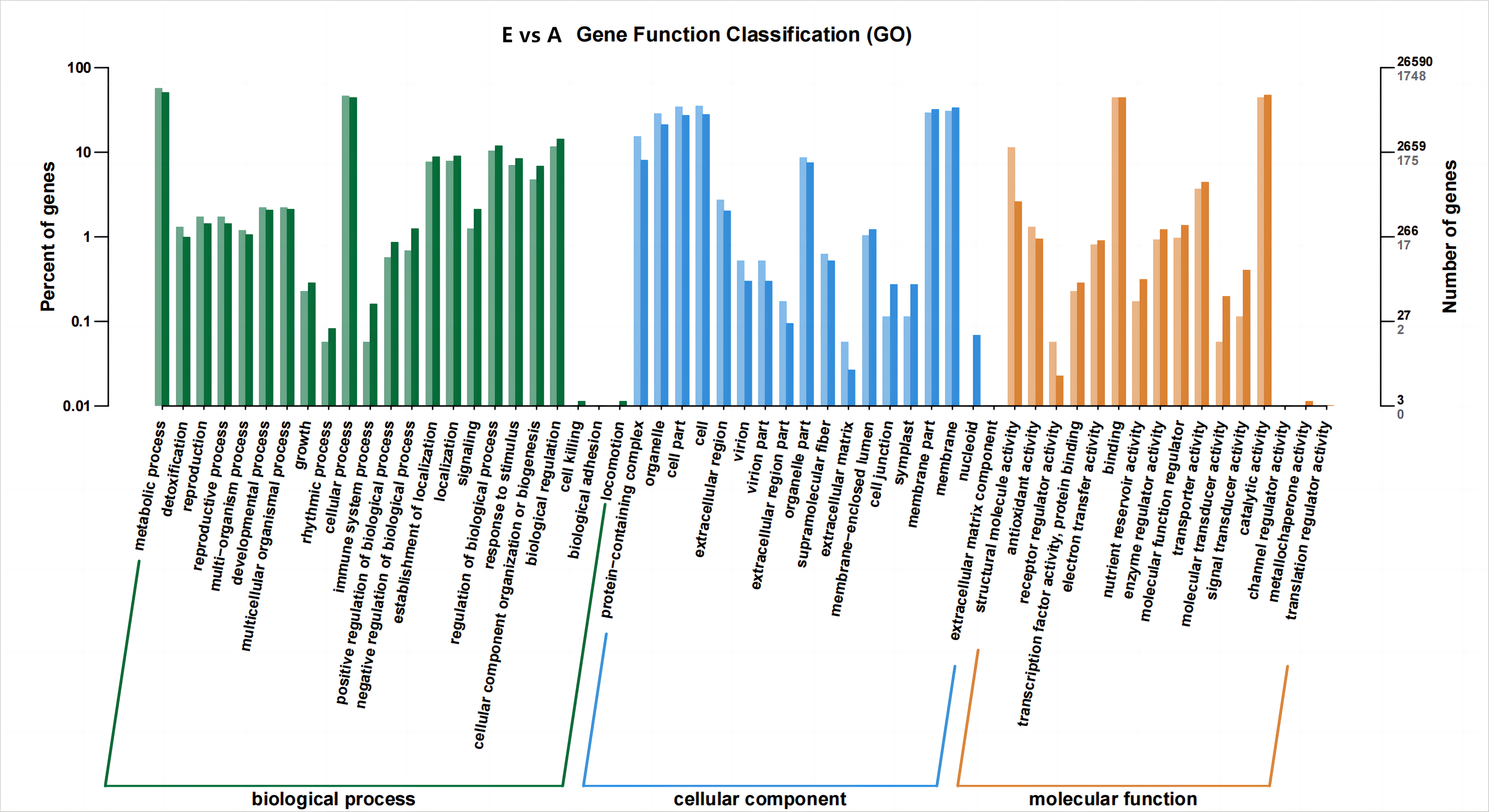

Supplement: Supplementary file 1 [file plants-14-02031-s001.zip › Supplementary Files/Supplementary Pictures/Figure S2-Gene Function Classification (GO)/Figure S2c-E_vs_A.sign_GO_categorie_00.jpg]

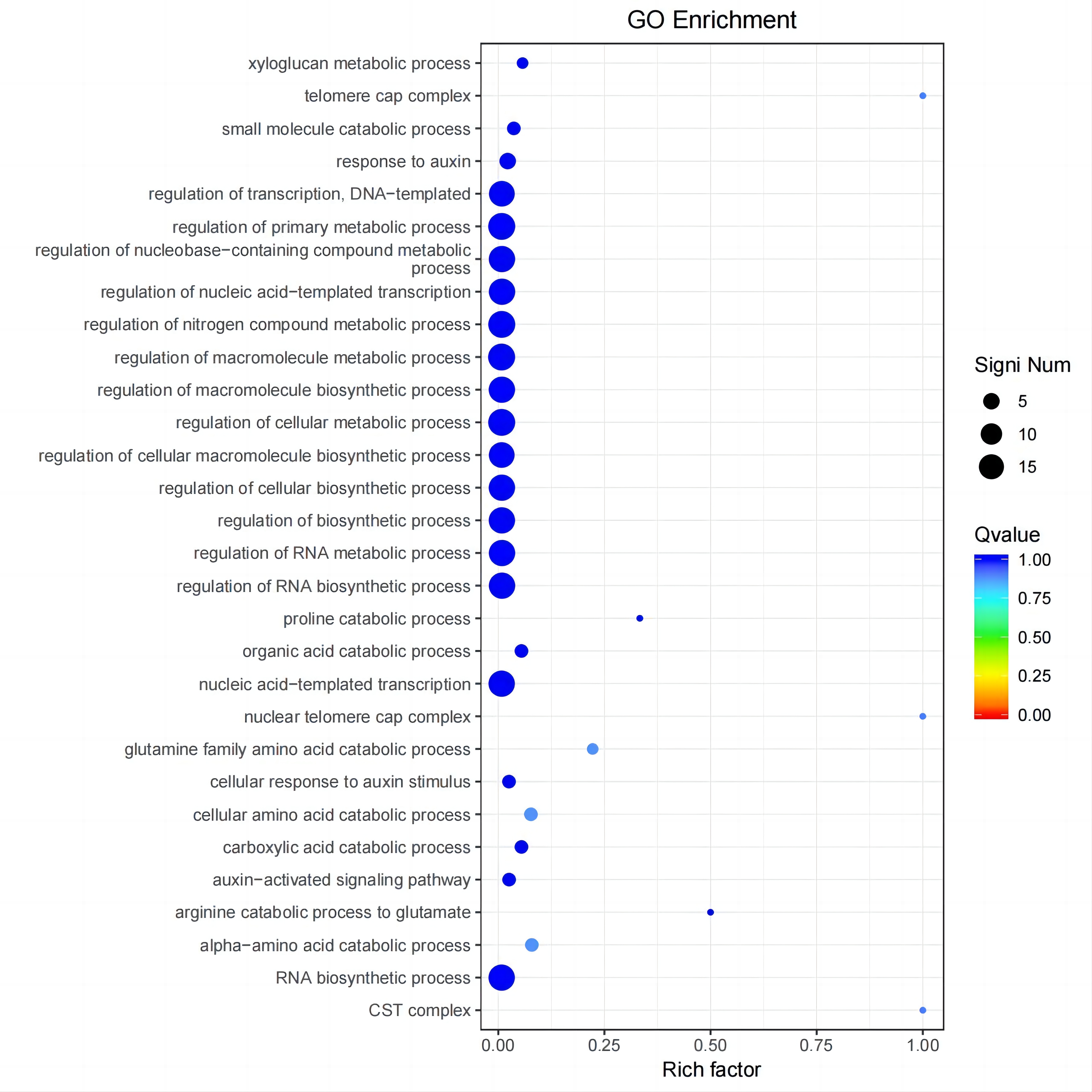

Supplement: Supplementary file 1 [file plants-14-02031-s001.zip › Supplementary Files/Supplementary Pictures/Figure S3-Bubble plots of the GO items in the GO enrichment analysis of DEGs/Figure S3a-B_vs_A.up_GO_enrichment_scatter_00.jpg]

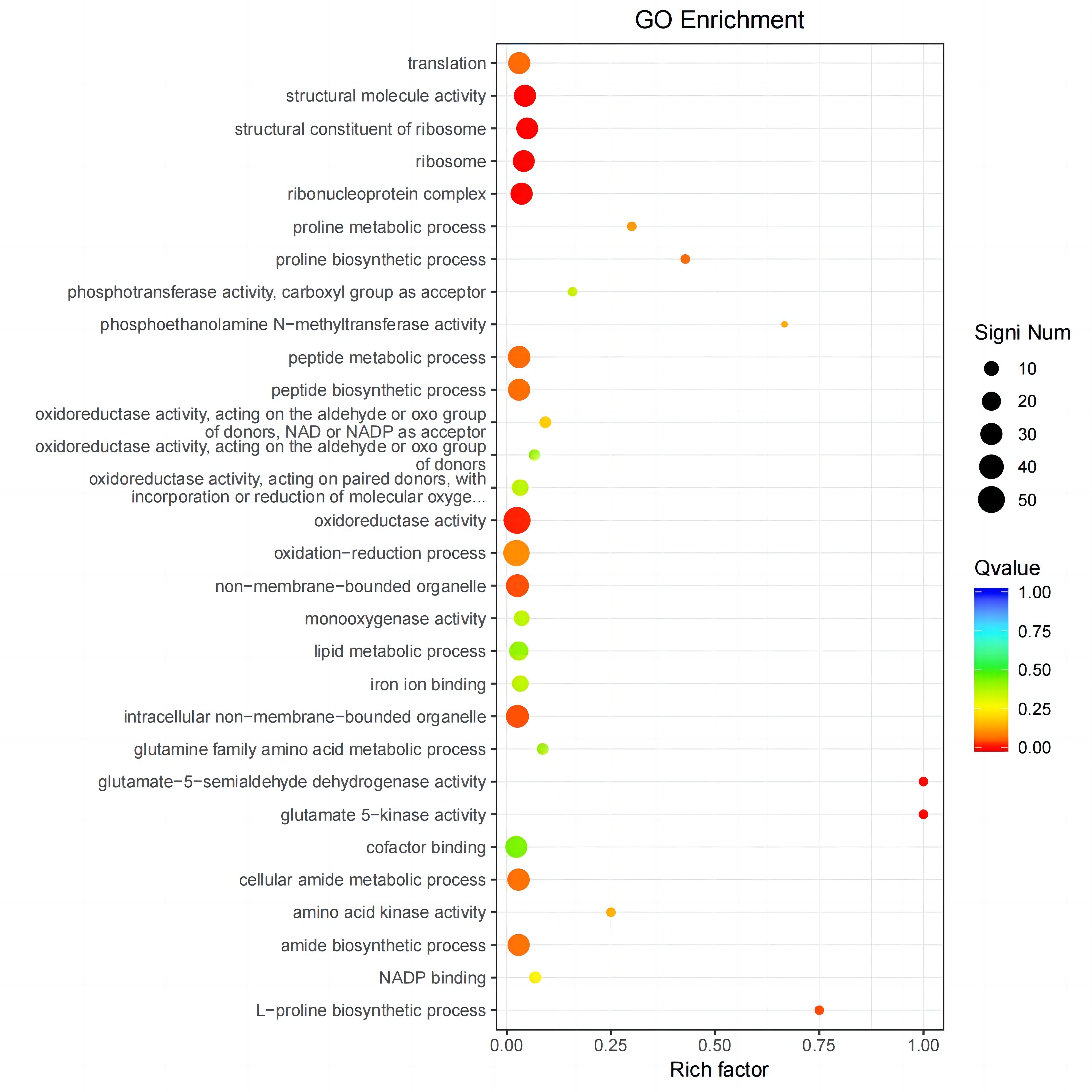

Supplement: Supplementary file 1 [file plants-14-02031-s001.zip › Supplementary Files/Supplementary Pictures/Figure S3-Bubble plots of the GO items in the GO enrichment analysis of DEGs/Figure S3b-C_vs_A.up_GO_enrichment_scatter_00.jpg]

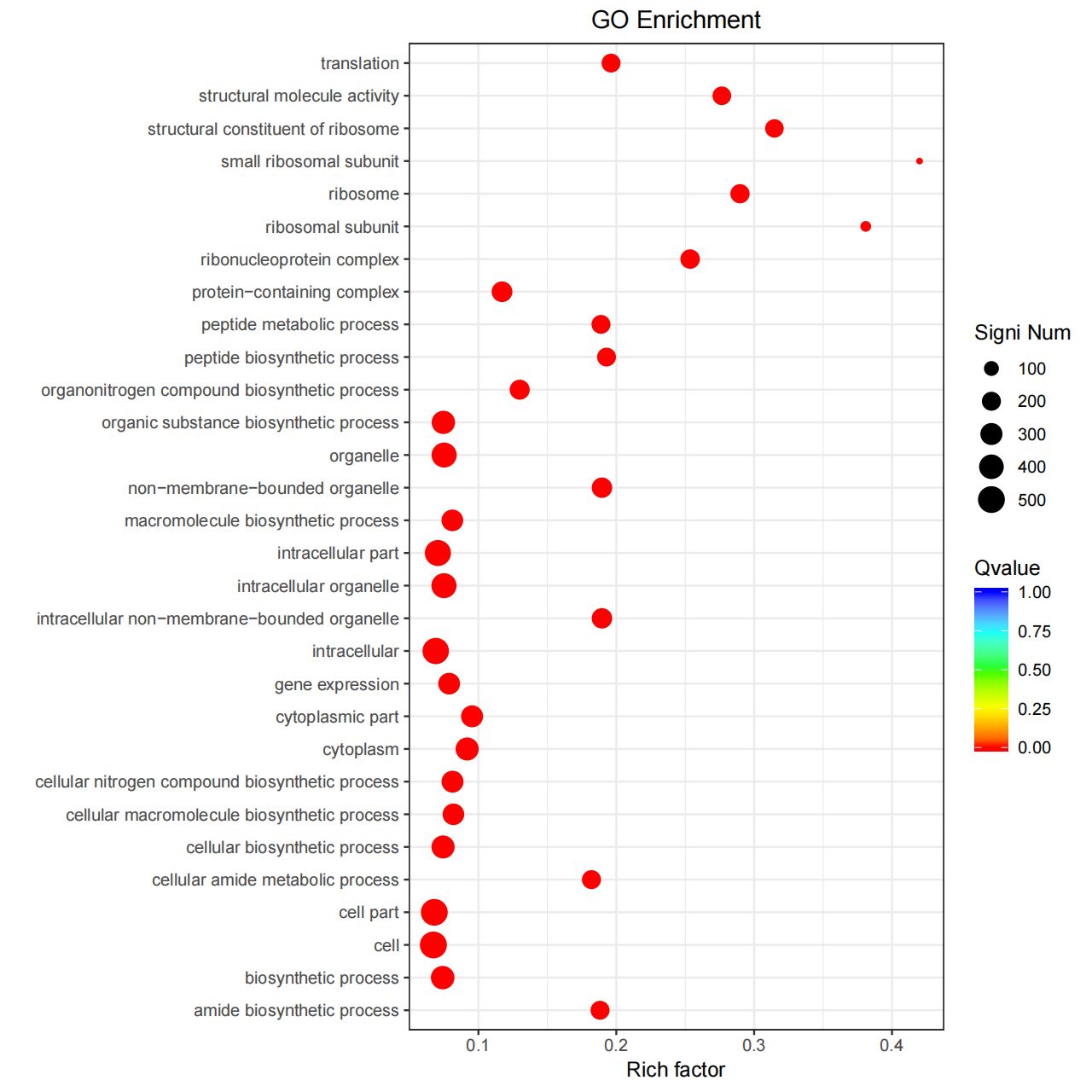

Supplement: Supplementary file 1 [file plants-14-02031-s001.zip › Supplementary Files/Supplementary Pictures/Figure S3-Bubble plots of the GO items in the GO enrichment analysis of DEGs/Figure S3c-E_vs_A.up_GO_enrichment_scatter_00.jpg]

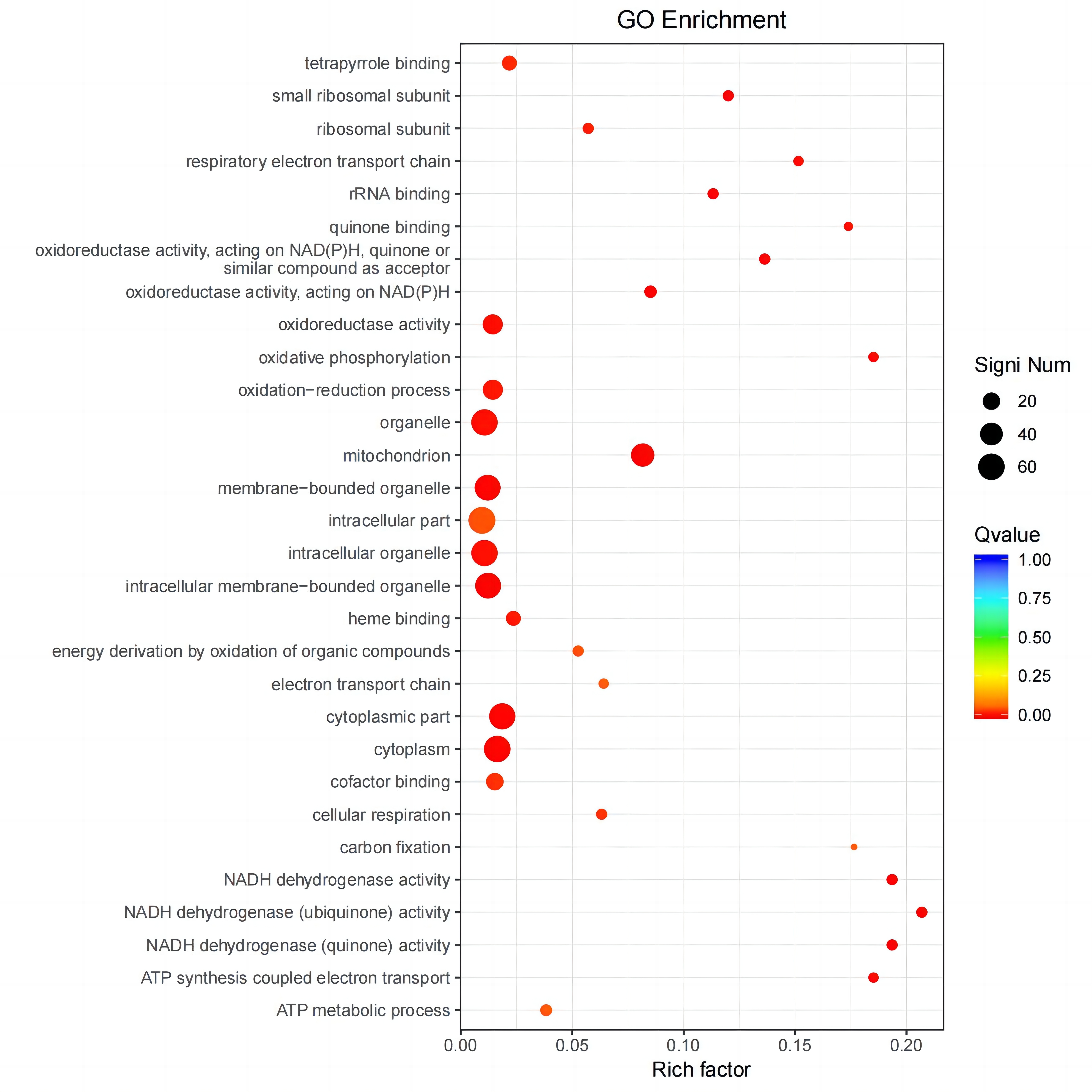

Supplement: Supplementary file 1 [file plants-14-02031-s001.zip › Supplementary Files/Supplementary Pictures/Figure S3-Bubble plots of the GO items in the GO enrichment analysis of DEGs/Figure S3d-B_vs_A.down_GO_enrichment_scatter_00.jpg]

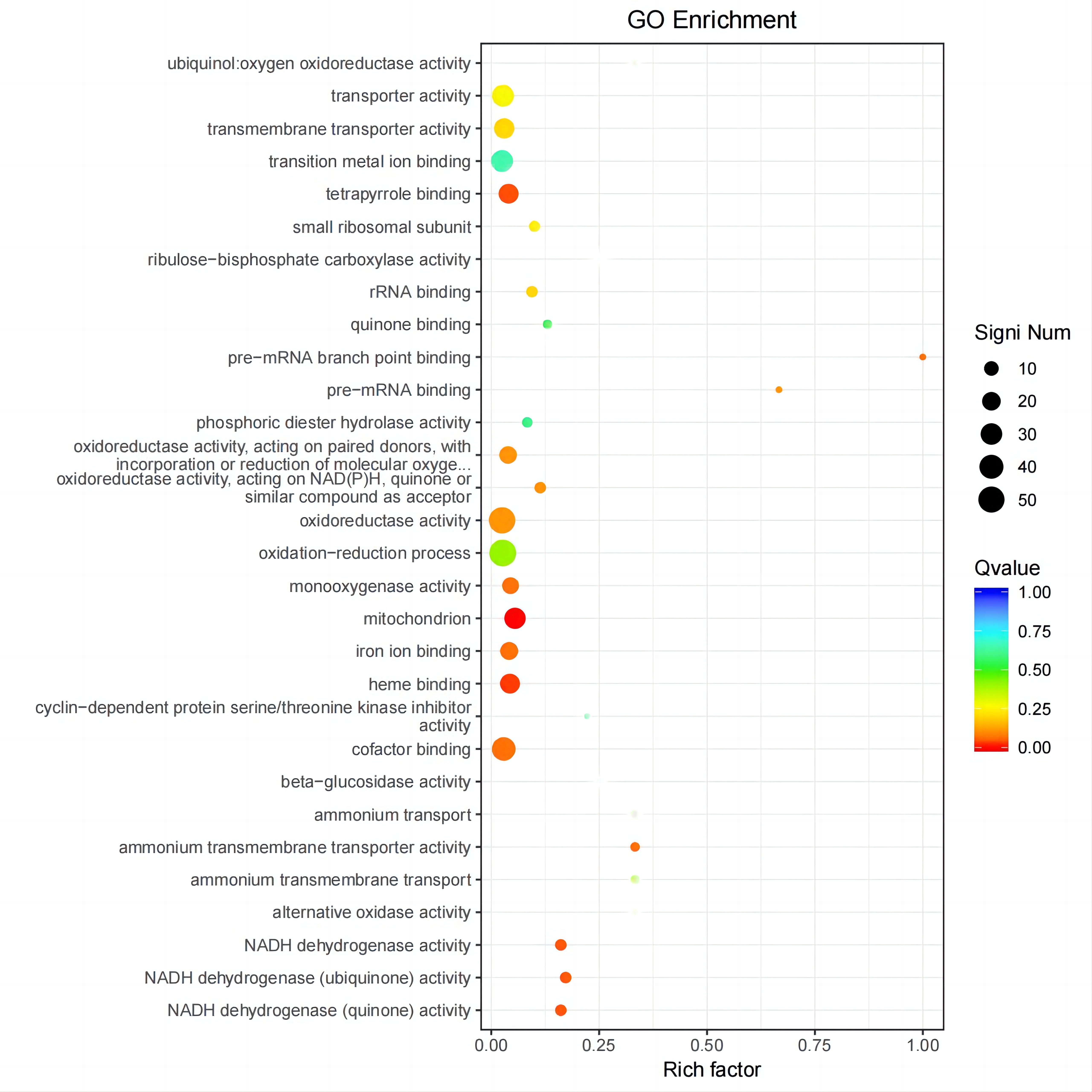

Supplement: Supplementary file 1 [file plants-14-02031-s001.zip › Supplementary Files/Supplementary Pictures/Figure S3-Bubble plots of the GO items in the GO enrichment analysis of DEGs/Figure S3e-C_vs_A.down_GO_enrichment_scatter_00.jpg]

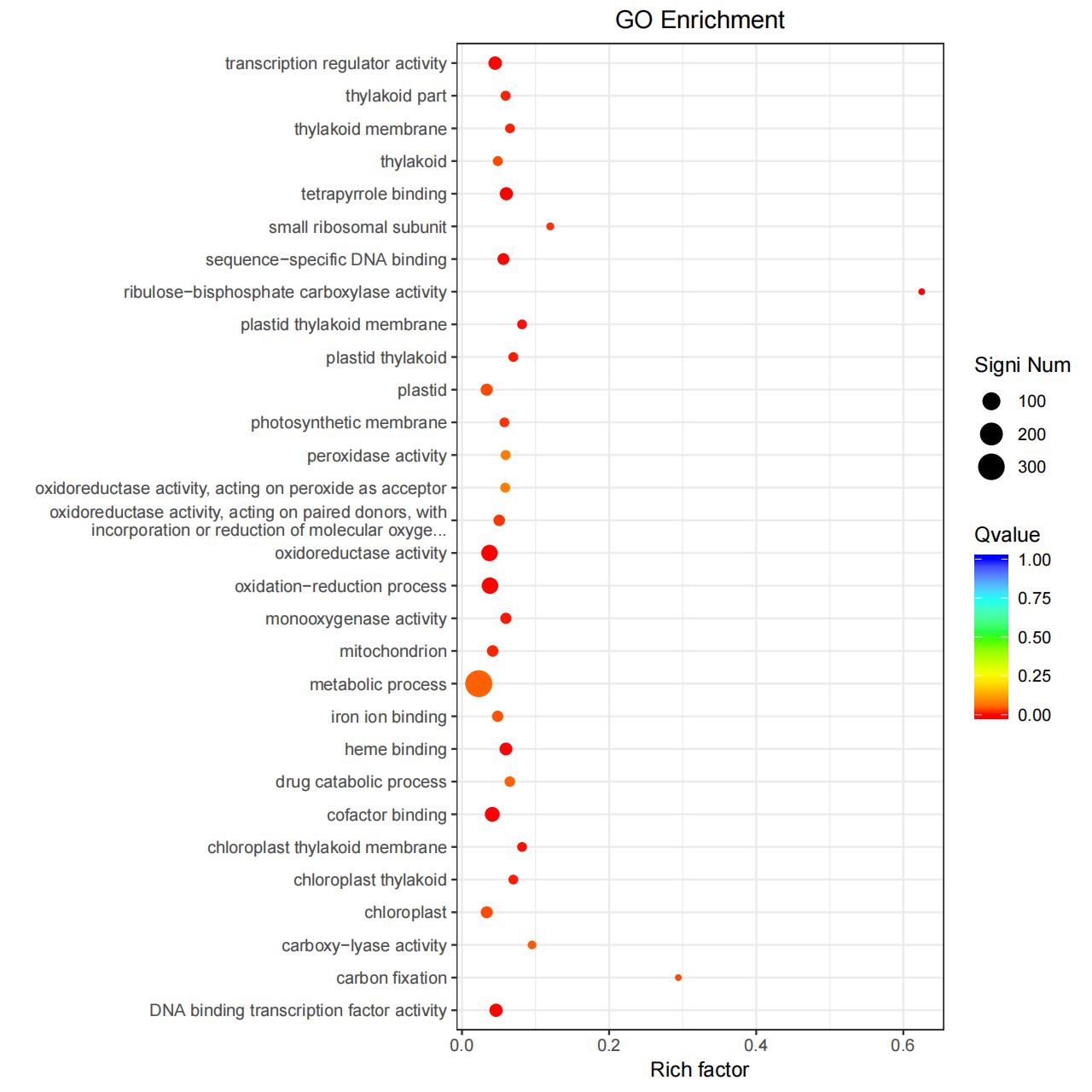

Supplement: Supplementary file 1 [file plants-14-02031-s001.zip › Supplementary Files/Supplementary Pictures/Figure S3-Bubble plots of the GO items in the GO enrichment analysis of DEGs/Figure S3f-E_vs_A.down_GO_enrichment_scatter_00.jpg]

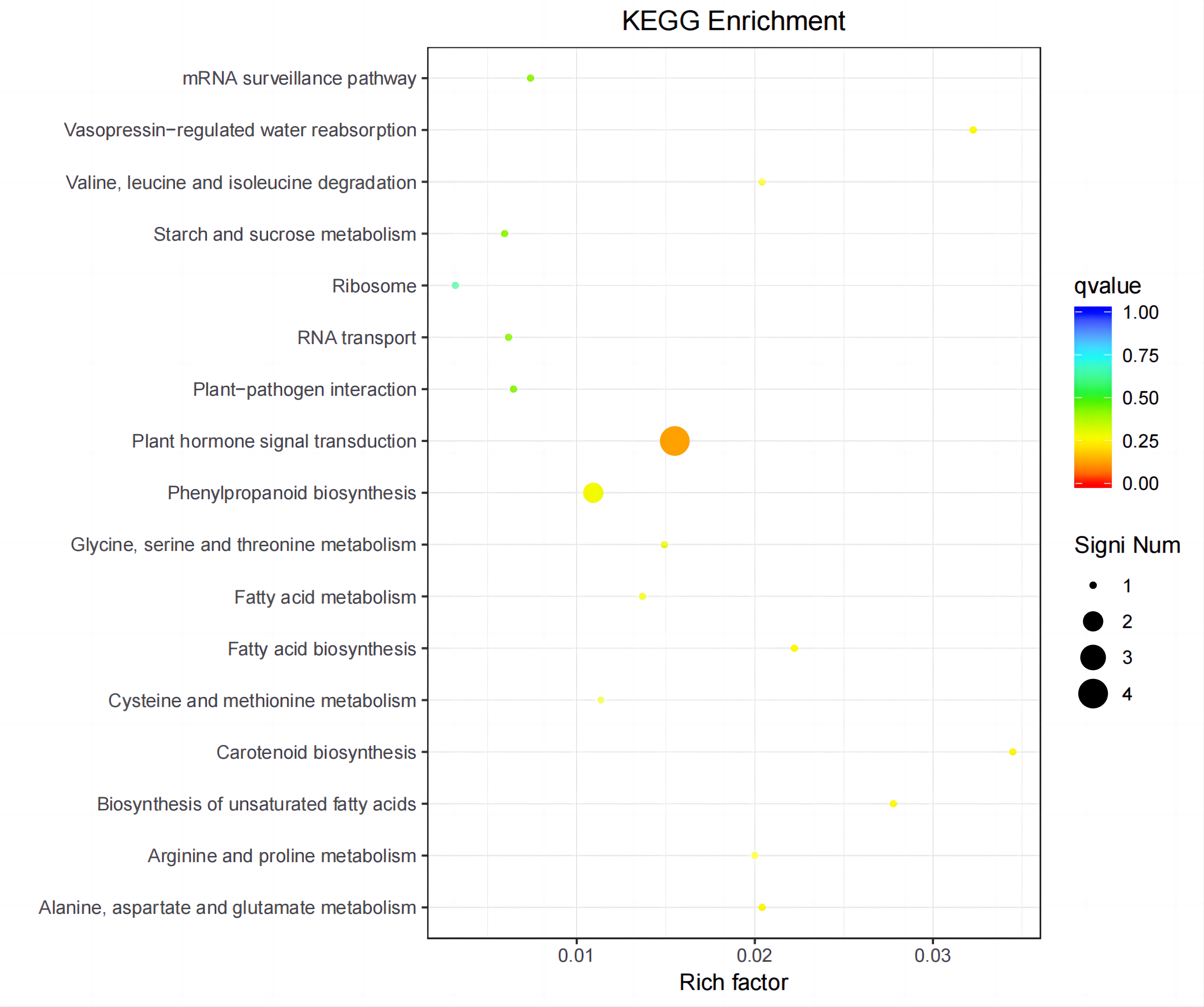

Supplement: Supplementary file 1 [file plants-14-02031-s001.zip › Supplementary Files/Supplementary Pictures/Figure S4-Bubble plots of the KEGG items in the GO enrichment analysis of DEGs/Figure S4a-B_vs_A.up_KEGG_enrichment_scatter_00.jpg]

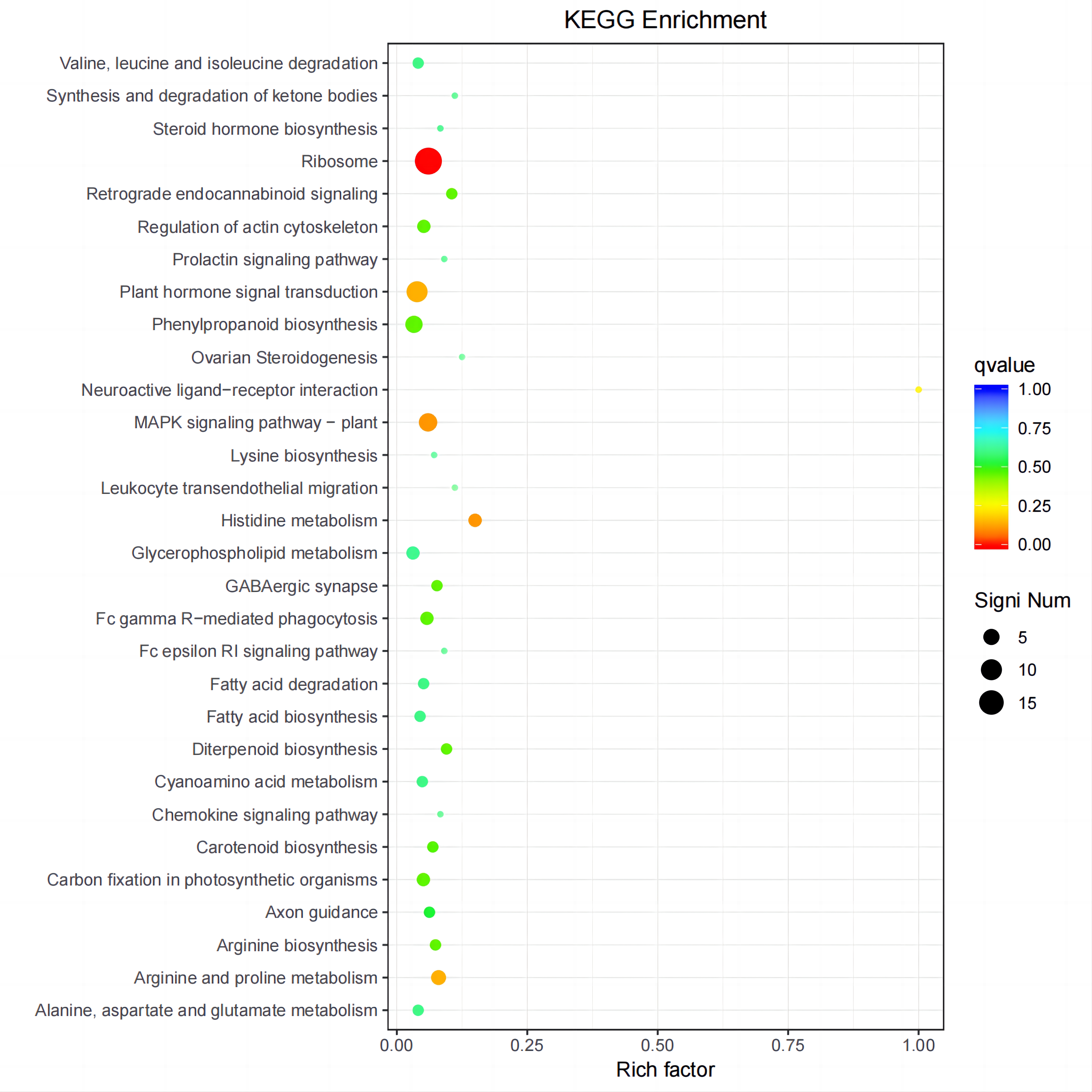

Supplement: Supplementary file 1 [file plants-14-02031-s001.zip › Supplementary Files/Supplementary Pictures/Figure S4-Bubble plots of the KEGG items in the GO enrichment analysis of DEGs/Figure S4b-C_vs_A.up_KEGG_enrichment_scatter_00.jpg]

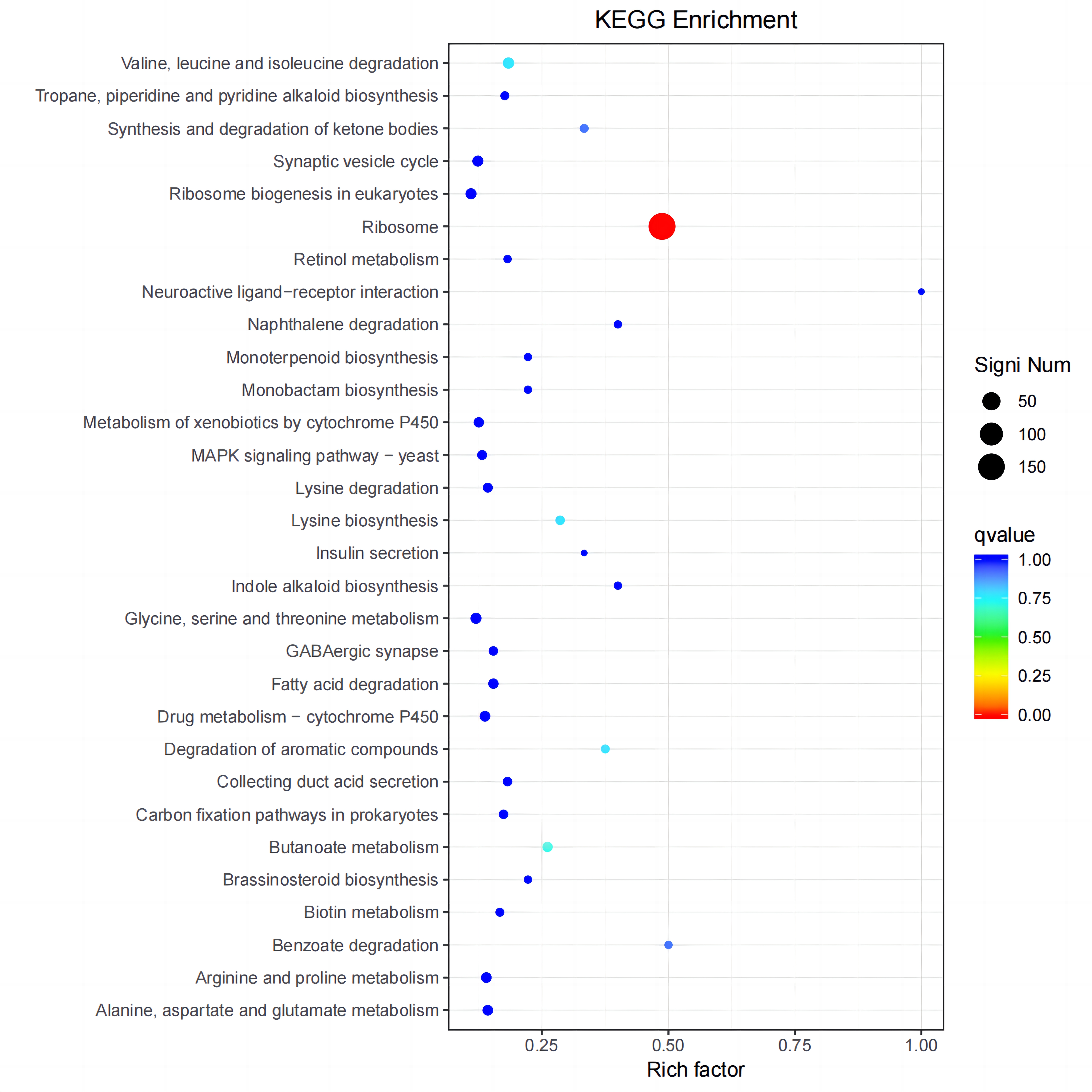

Supplement: Supplementary file 1 [file plants-14-02031-s001.zip › Supplementary Files/Supplementary Pictures/Figure S4-Bubble plots of the KEGG items in the GO enrichment analysis of DEGs/Figure S4c-E_vs_A.up_KEGG_enrichment_scatter_00.jpg]

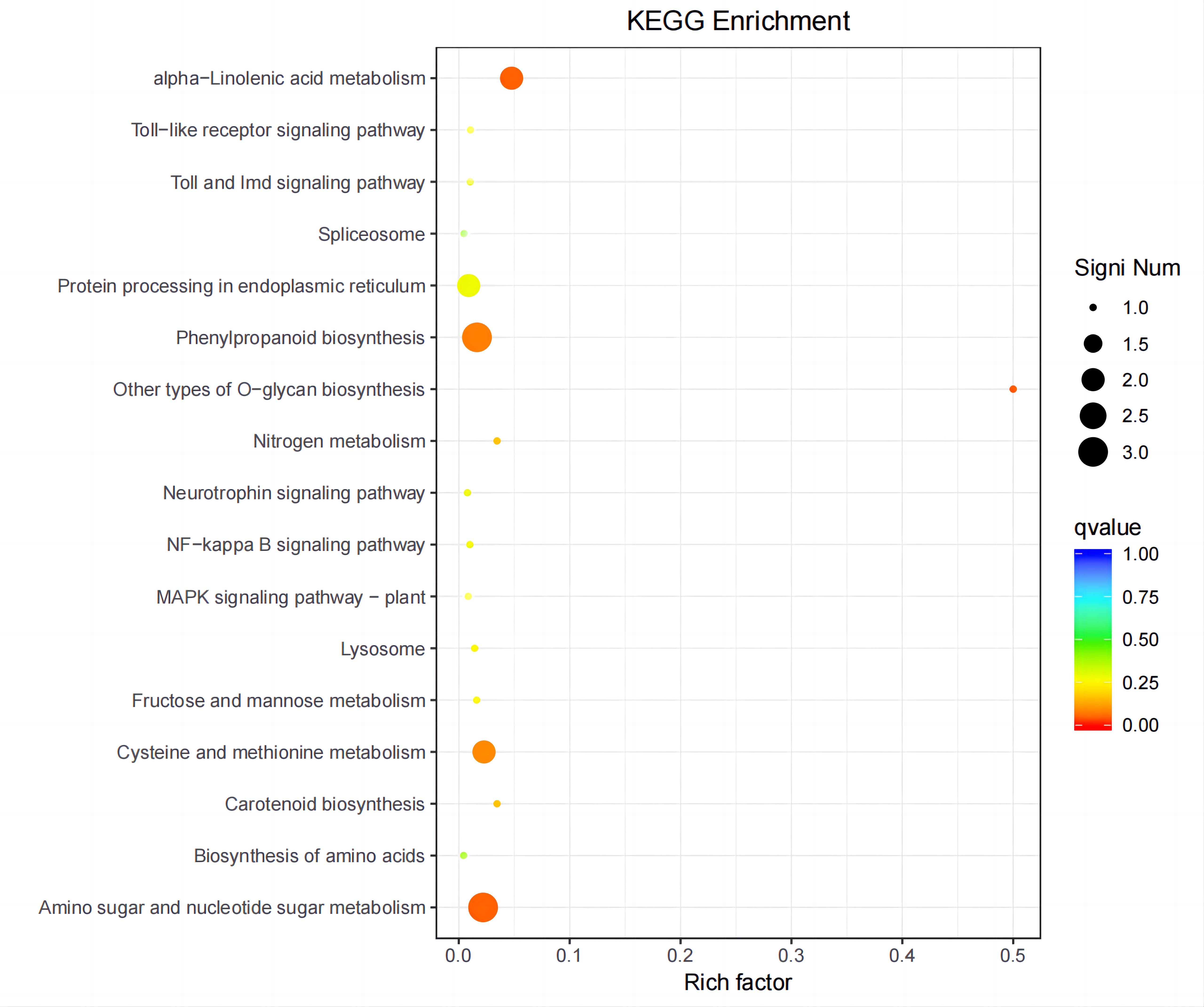

Supplement: Supplementary file 1 [file plants-14-02031-s001.zip › Supplementary Files/Supplementary Pictures/Figure S4-Bubble plots of the KEGG items in the GO enrichment analysis of DEGs/Figure S4d-B_vs_A.down_KEGG_enrichment_scatter_00.jpg]

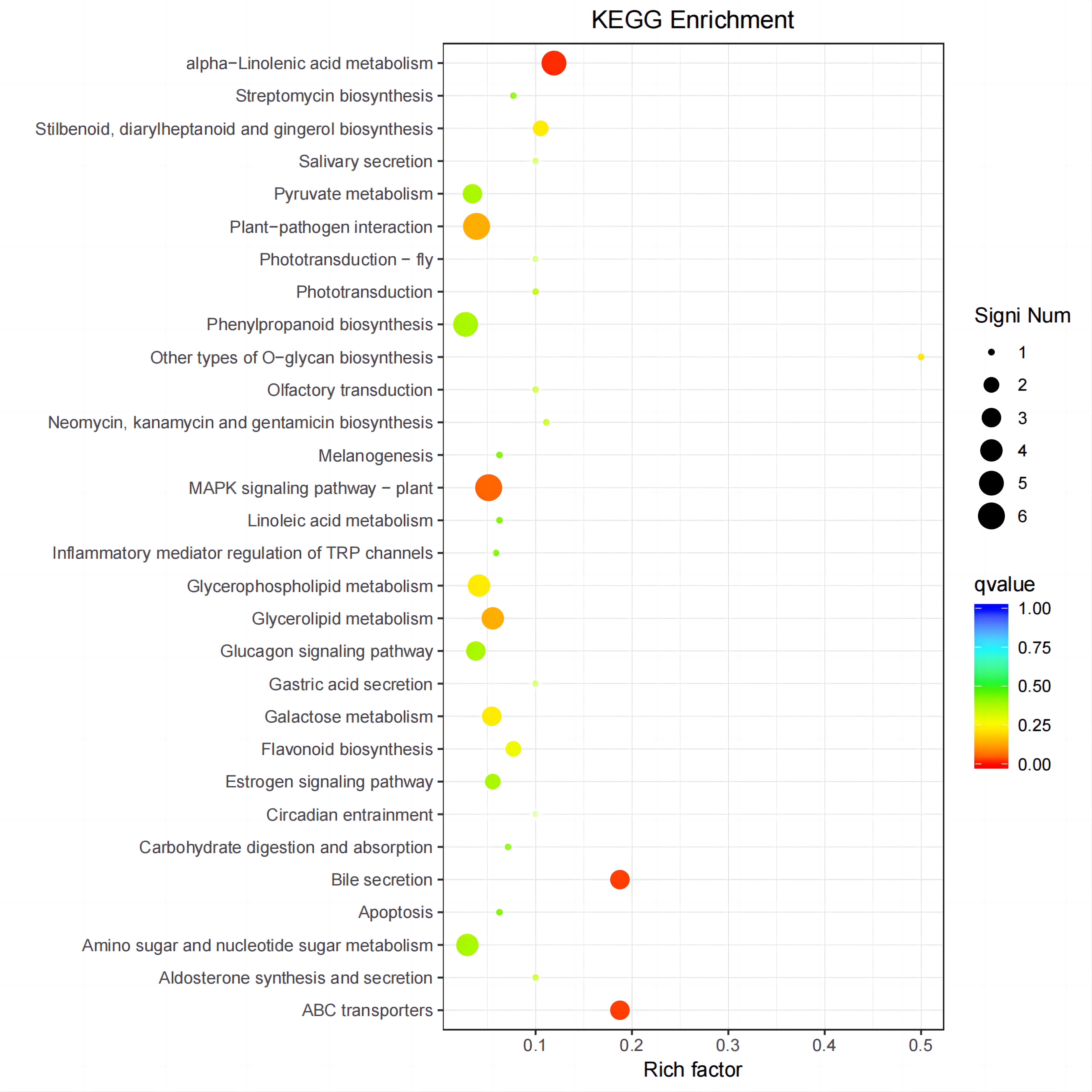

Supplement: Supplementary file 1 [file plants-14-02031-s001.zip › Supplementary Files/Supplementary Pictures/Figure S4-Bubble plots of the KEGG items in the GO enrichment analysis of DEGs/Figure S4e-C_vs_A.down_KEGG_enrichment_scatter_00.jpg]

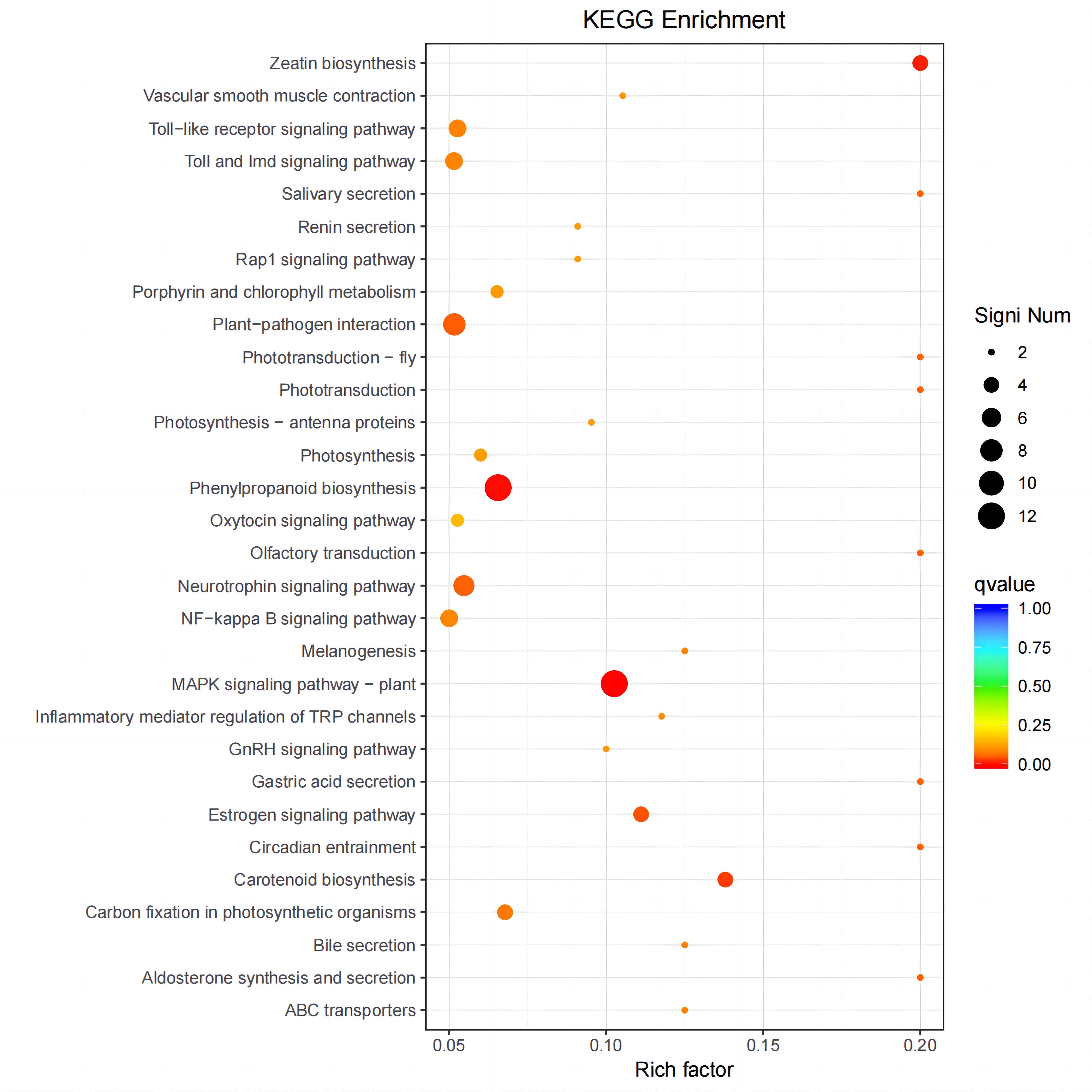

Supplement: Supplementary file 1 [file plants-14-02031-s001.zip › Supplementary Files/Supplementary Pictures/Figure S4-Bubble plots of the KEGG items in the GO enrichment analysis of DEGs/Figure S4f-E_vs_A.down_KEGG_enrichment_scatter_00.jpg]

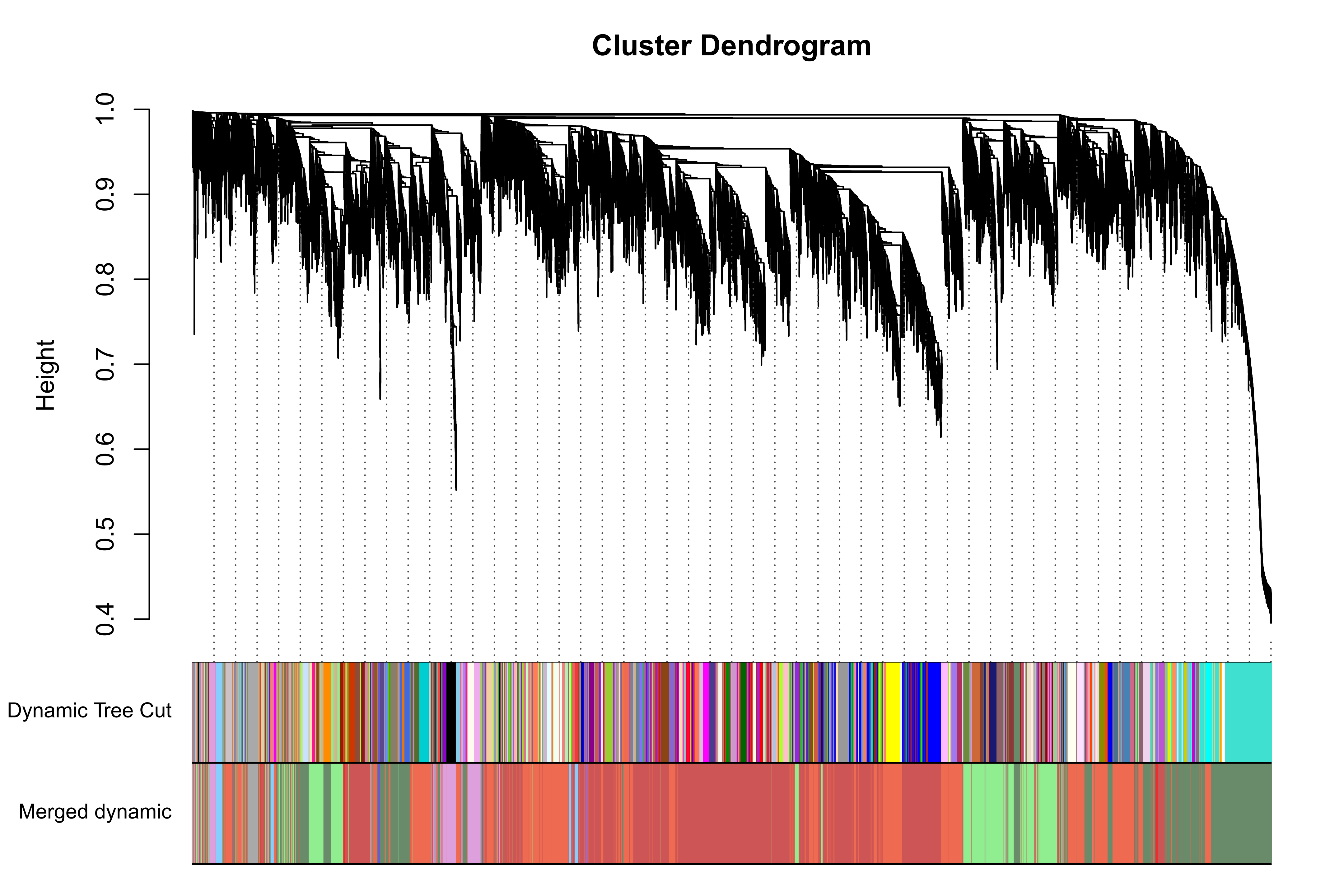

Supplement: Supplementary file 1 [file plants-14-02031-s001.zip › Supplementary Files/Supplementary Pictures/Figure S5-WGCNA Cluster dendrogram and Heatmap of the identified modules(Gene)/Figure S5a-cluster dendrogram of the identified modules.png]

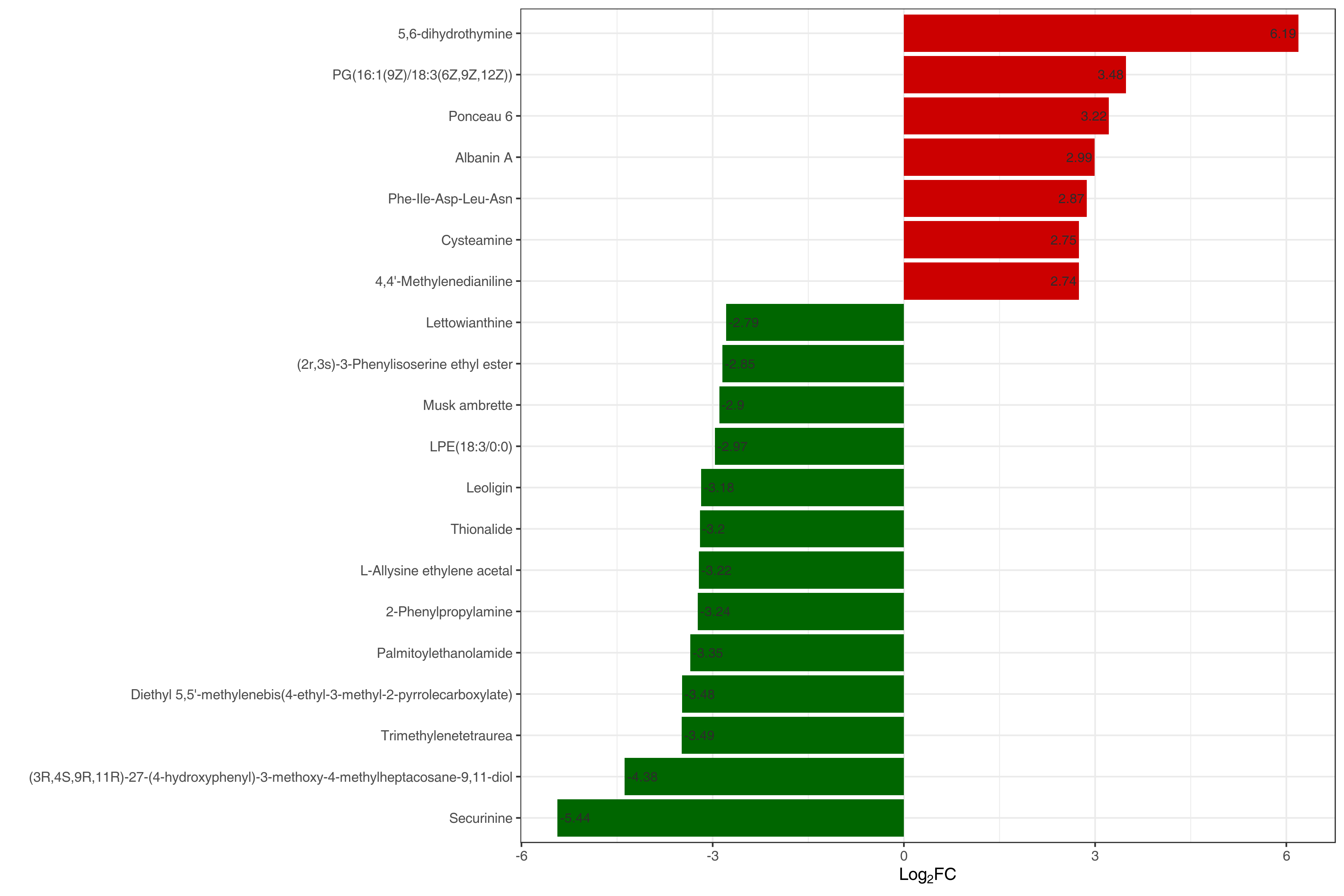

Supplement: Supplementary file 1 [file plants-14-02031-s001.zip › Supplementary Files/Supplementary Pictures/Figure S6-Metabolomic analysis/Figure S6b-B_vs_A_TopFcBarChart_Compounds.png]

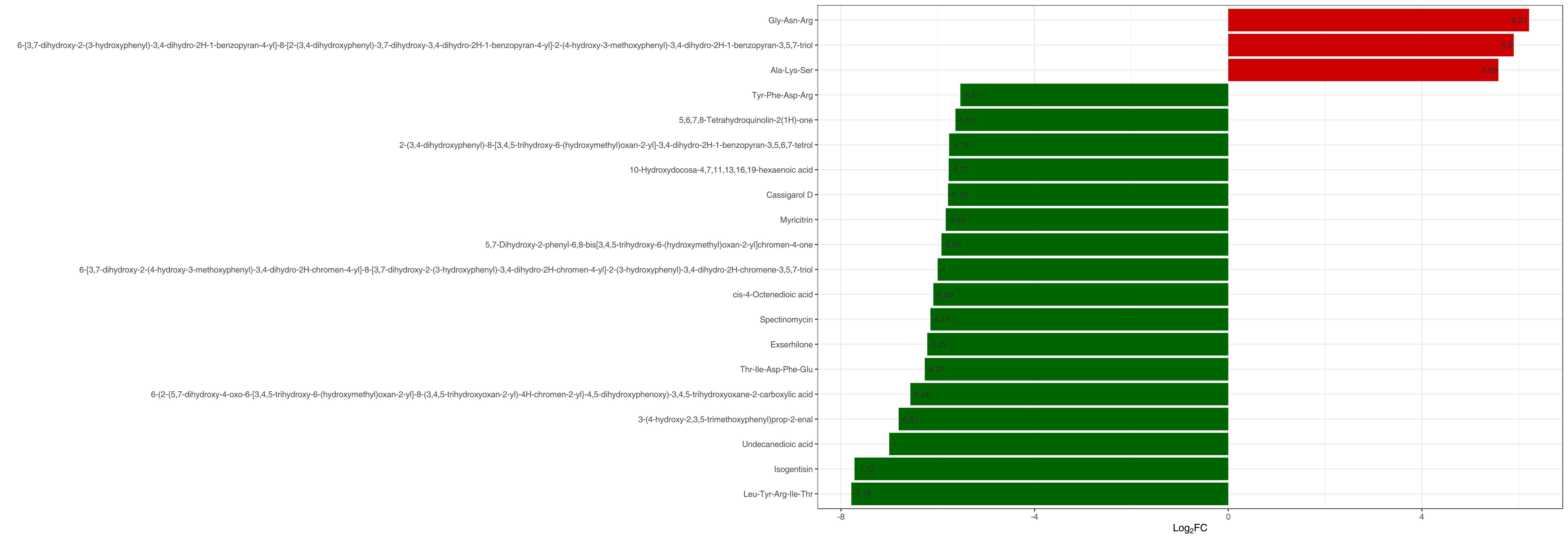

Supplement: Supplementary file 1 [file plants-14-02031-s001.zip › Supplementary Files/Supplementary Pictures/Figure S6-Metabolomic analysis/Figure S6c-C_vs_A_TopFcBarChart_Compounds.png]

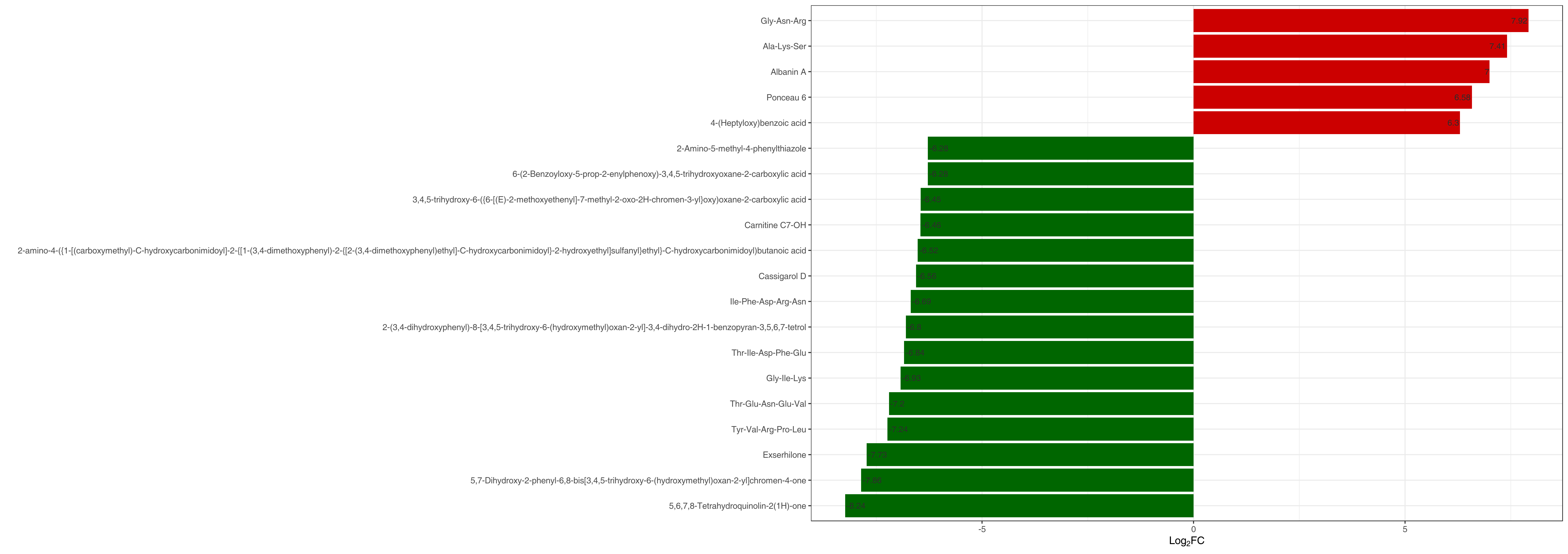

Supplement: Supplementary file 1 [file plants-14-02031-s001.zip › Supplementary Files/Supplementary Pictures/Figure S6-Metabolomic analysis/Figure S6d-E_vs_A_TopFcBarChart_Compounds.png]

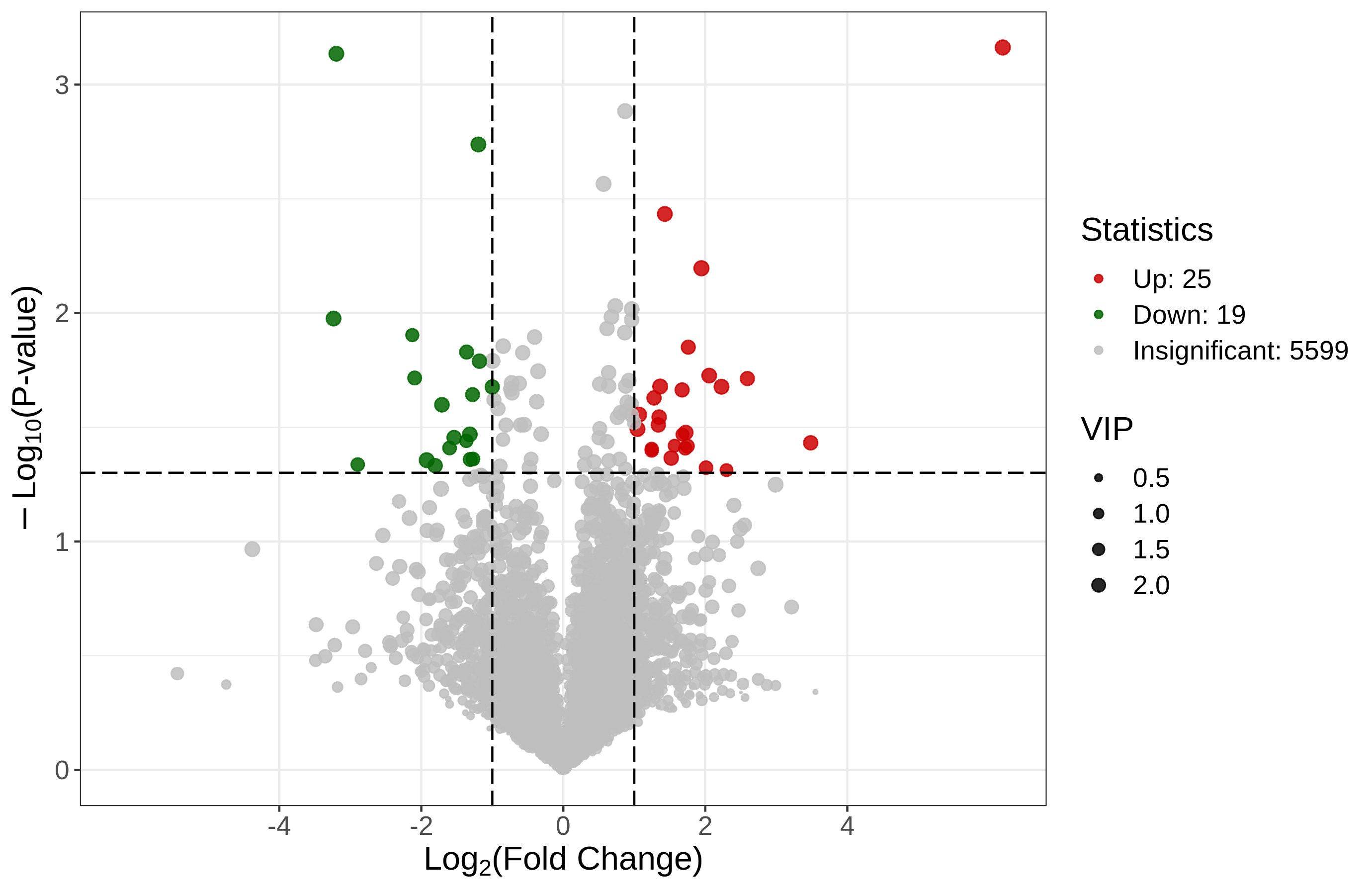

Supplement: Supplementary file 1 [file plants-14-02031-s001.zip › Supplementary Files/Supplementary Pictures/Figure S6-Metabolomic analysis/Figure S6e-B_vs_A_volcano_Log2FC_Pvalue_VIP.png]

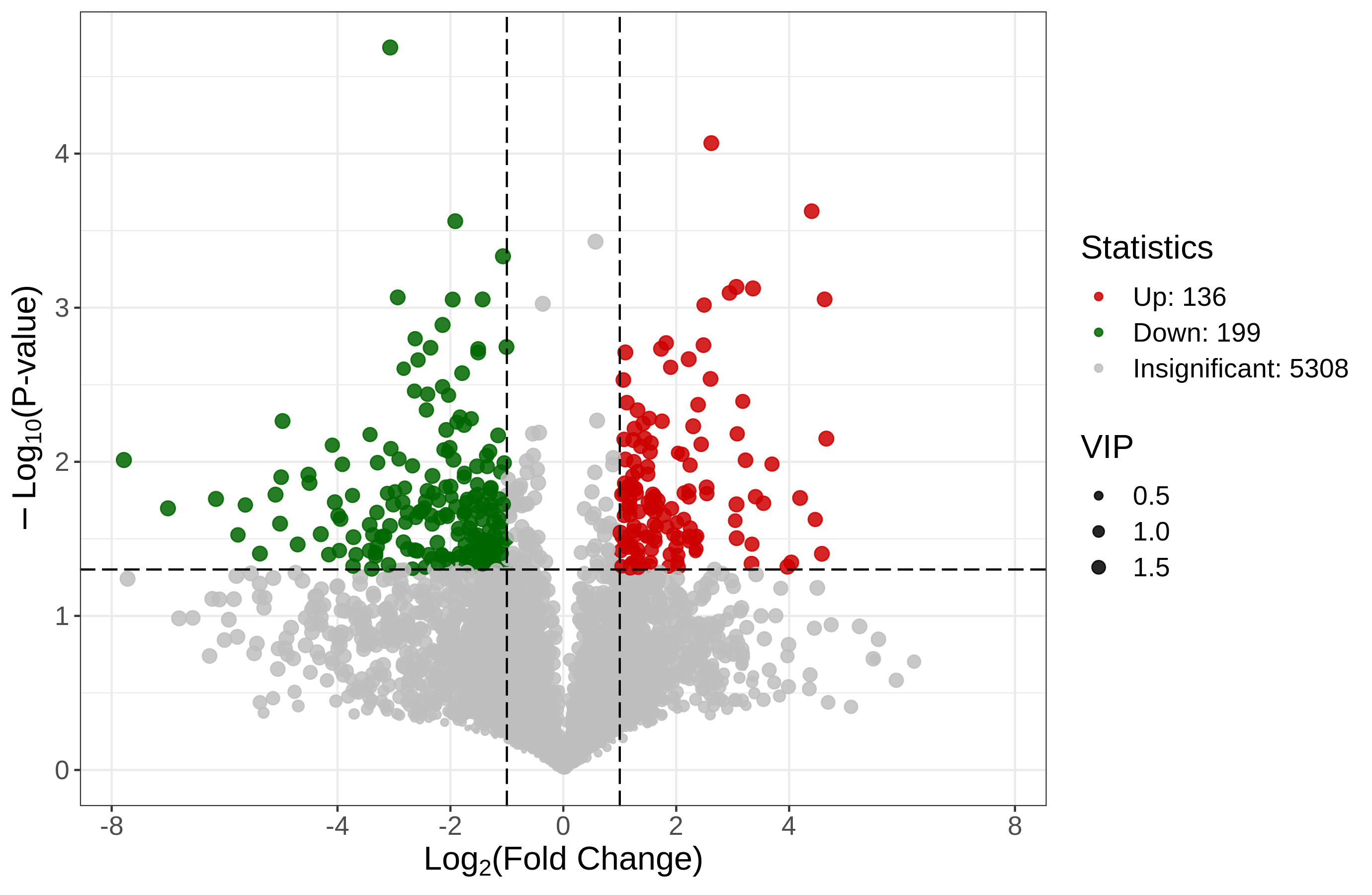

Supplement: Supplementary file 1 [file plants-14-02031-s001.zip › Supplementary Files/Supplementary Pictures/Figure S6-Metabolomic analysis/Figure S6f-C_vs_A_volcano_Log2FC_Pvalue_VIP.png]

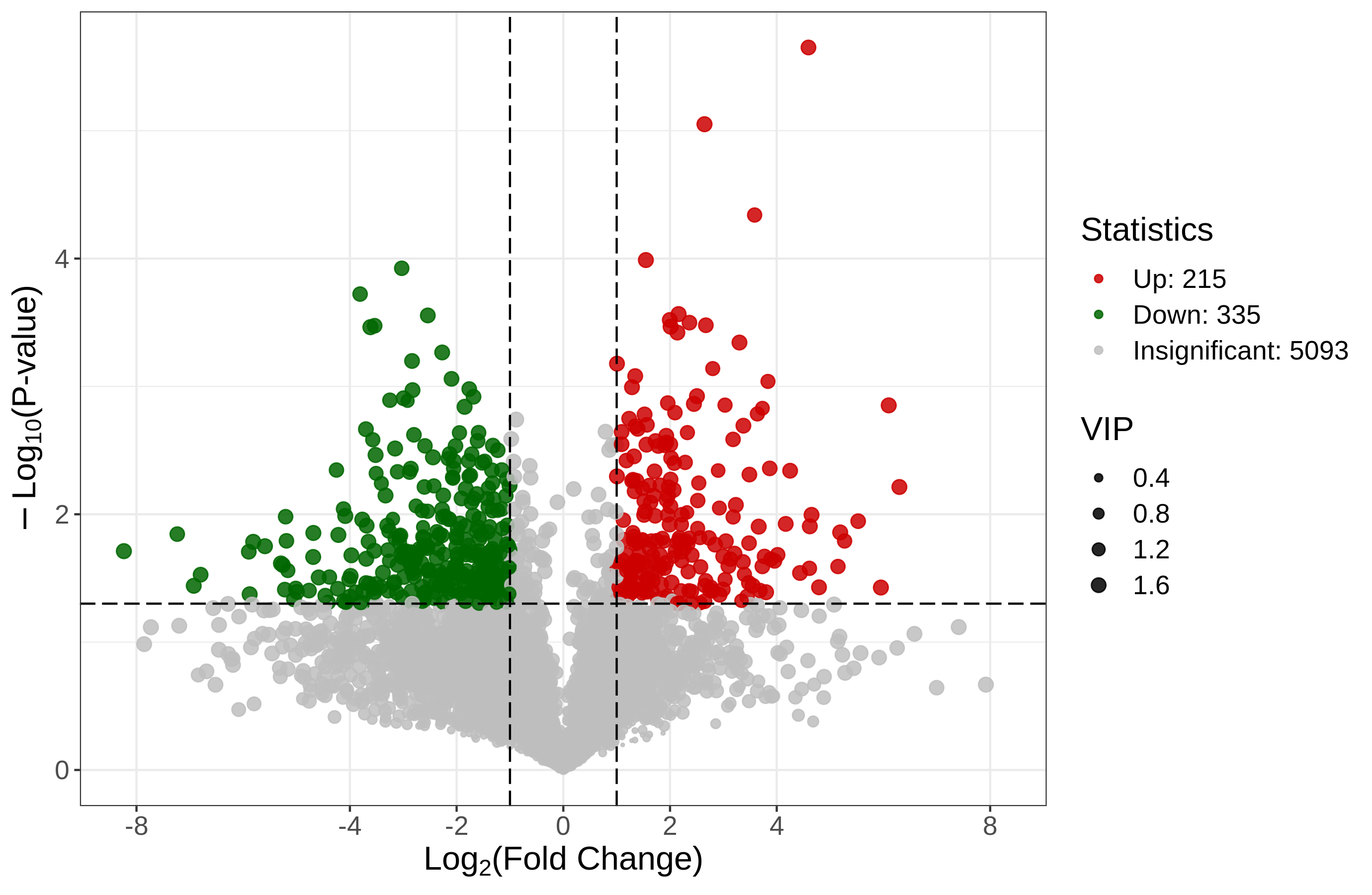

Supplement: Supplementary file 1 [file plants-14-02031-s001.zip › Supplementary Files/Supplementary Pictures/Figure S6-Metabolomic analysis/Figure S6g-E_vs_A_volcano_Log2FC_Pvalue_VIP.png]

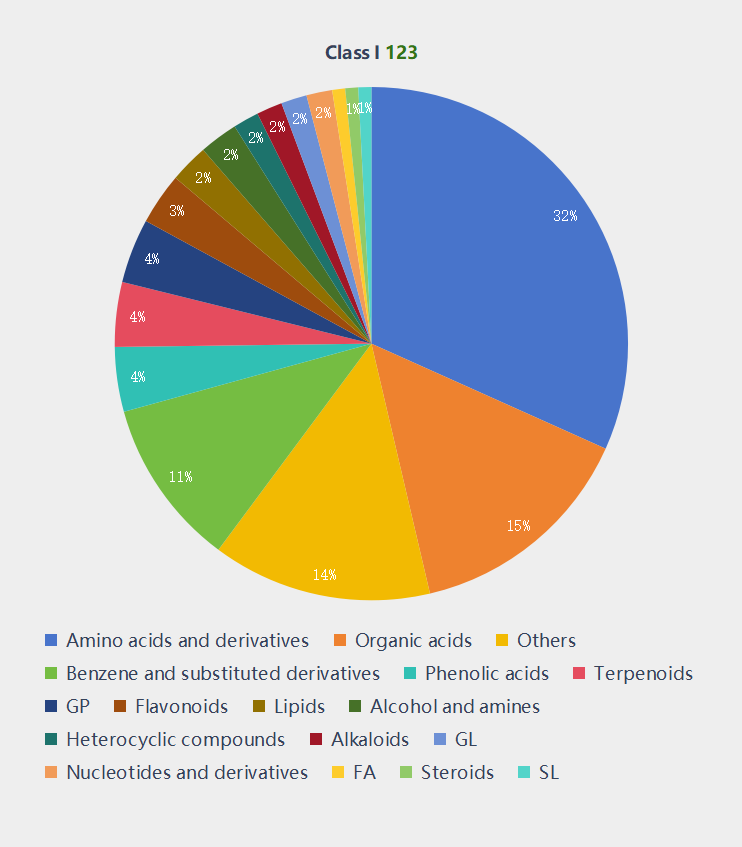

Supplement: Supplementary file 1 [file plants-14-02031-s001.zip › Supplementary Files/Supplementary Pictures/Figure S6-Metabolomic analysis/FigureS6h-.Statistical analysis of DEMs Class I.png]

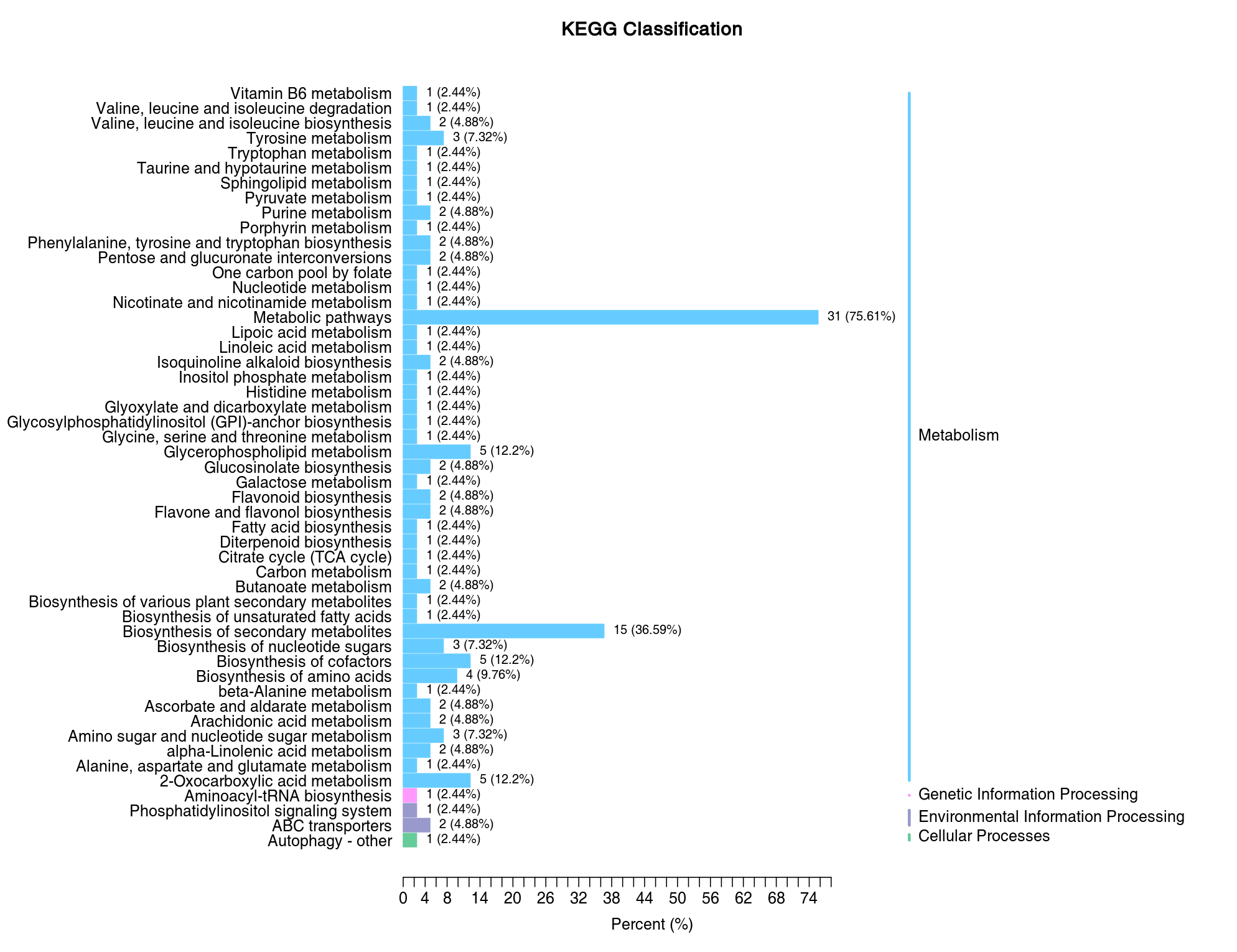

Supplement: Supplementary file 1 [file plants-14-02031-s001.zip › Supplementary Files/Supplementary Pictures/Figure S7-KEGG classification and enrichment/Figure S7a-B_vs_A_KEGG_barplot.png]

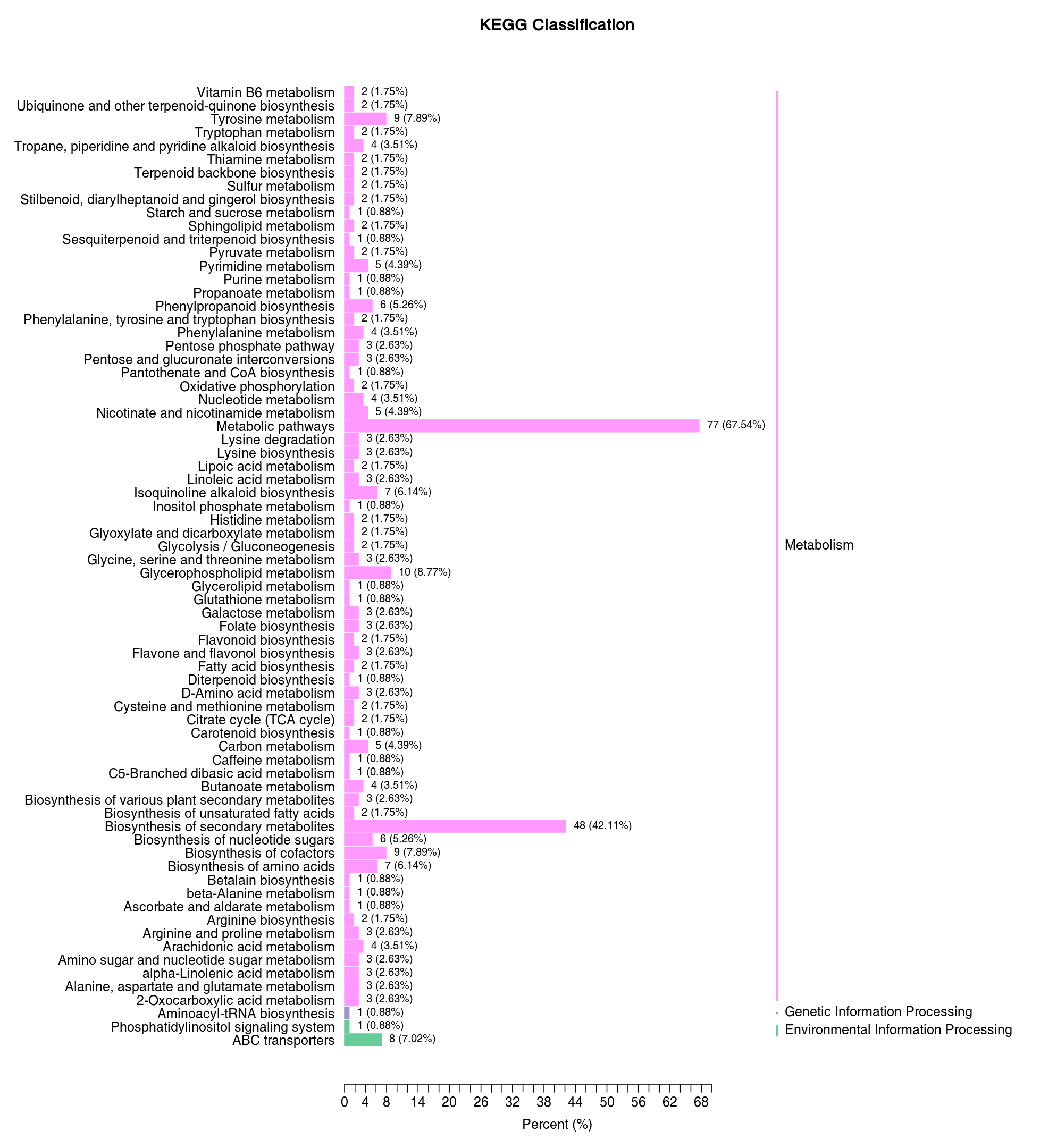

Supplement: Supplementary file 1 [file plants-14-02031-s001.zip › Supplementary Files/Supplementary Pictures/Figure S7-KEGG classification and enrichment/Figure S7b-C_vs_A_KEGG_barplot.png]

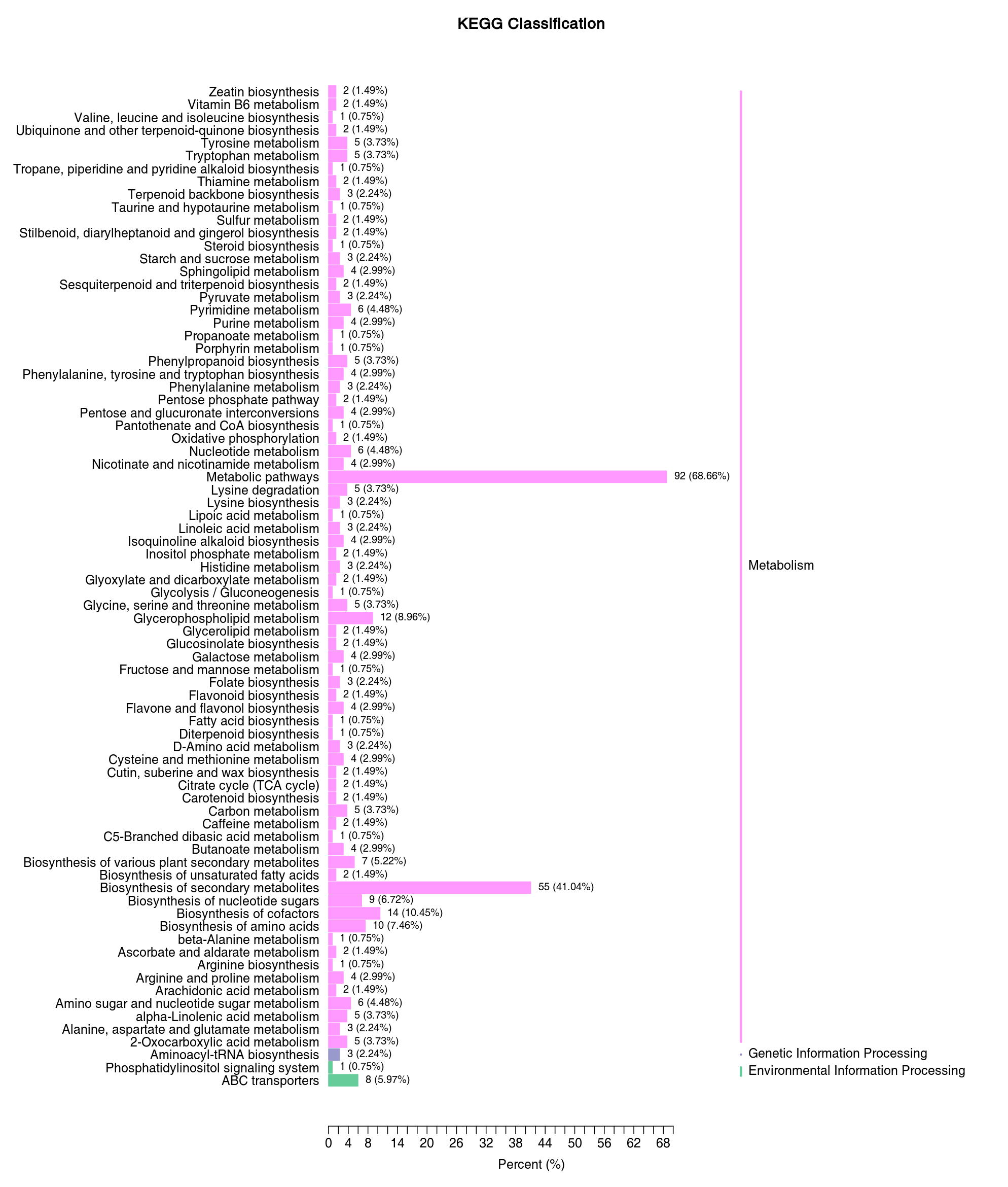

Supplement: Supplementary file 1 [file plants-14-02031-s001.zip › Supplementary Files/Supplementary Pictures/Figure S7-KEGG classification and enrichment/Figure S7c-E_vs_A_KEGG_barplot.png]

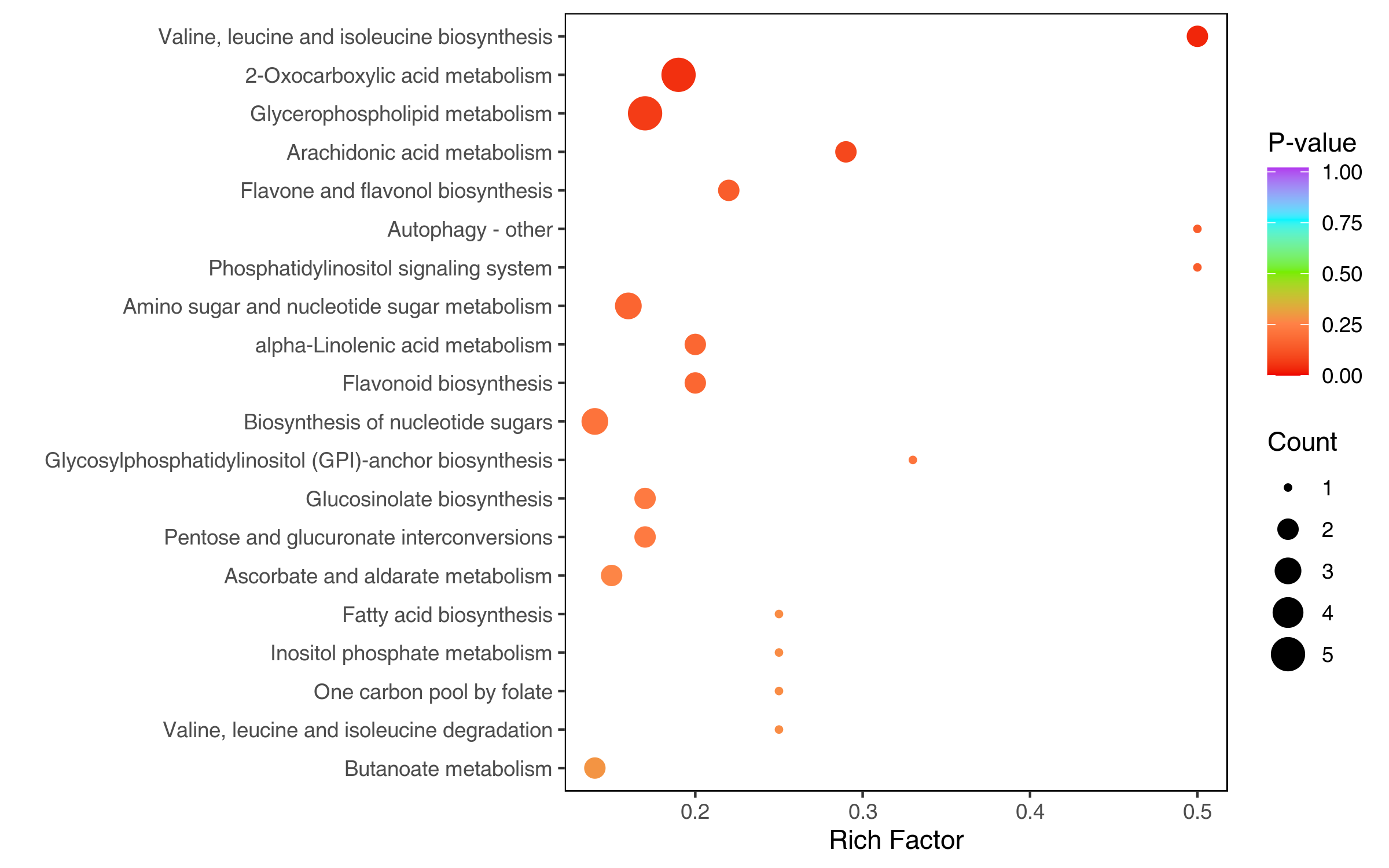

Supplement: Supplementary file 1 [file plants-14-02031-s001.zip › Supplementary Files/Supplementary Pictures/Figure S7-KEGG classification and enrichment/Figure S7d-B_vs_A_KEGG_Enrichment_P-value.png]

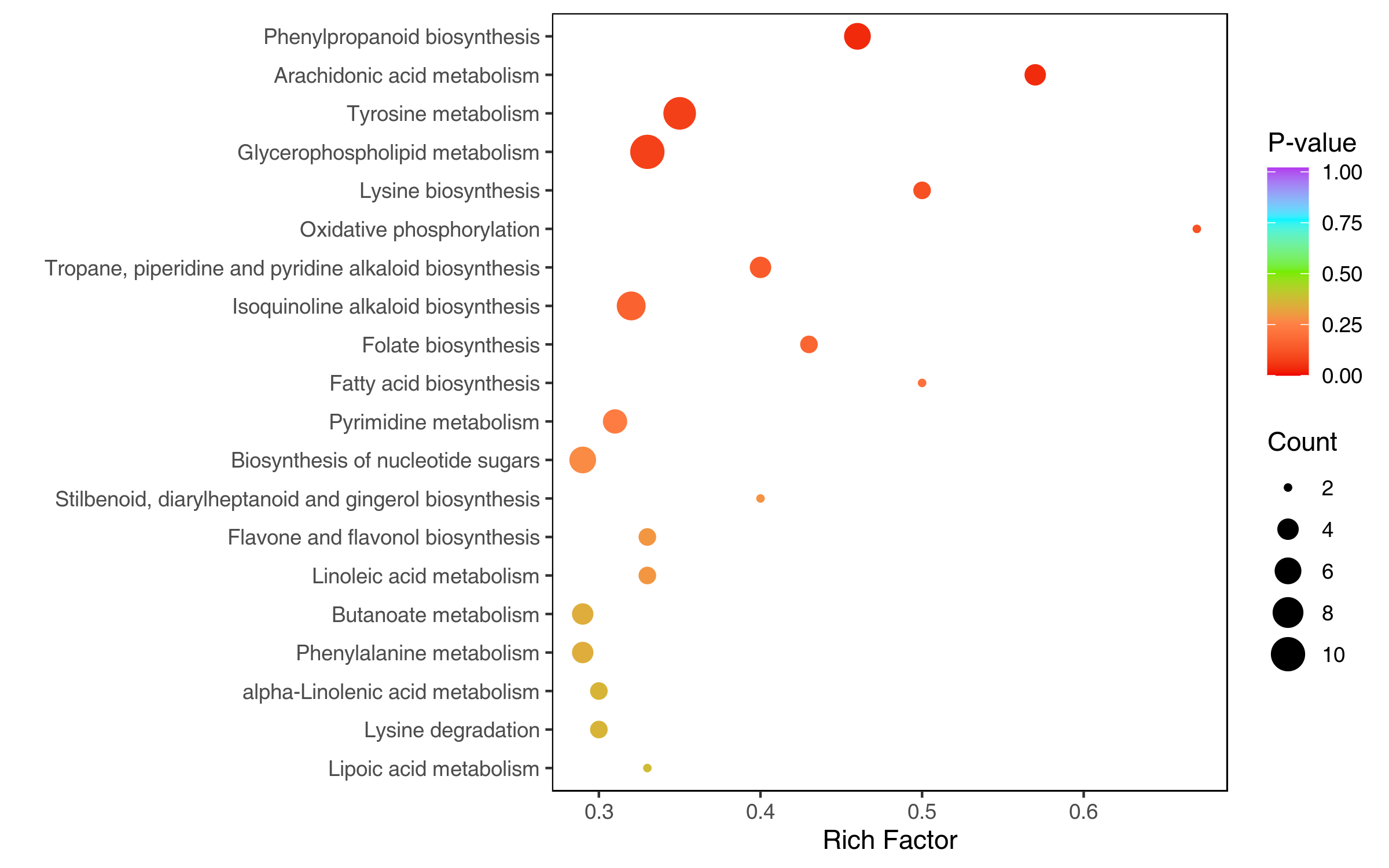

Supplement: Supplementary file 1 [file plants-14-02031-s001.zip › Supplementary Files/Supplementary Pictures/Figure S7-KEGG classification and enrichment/Figure S7e-C_vs_A_KEGG_Enrichment_P-value.png]

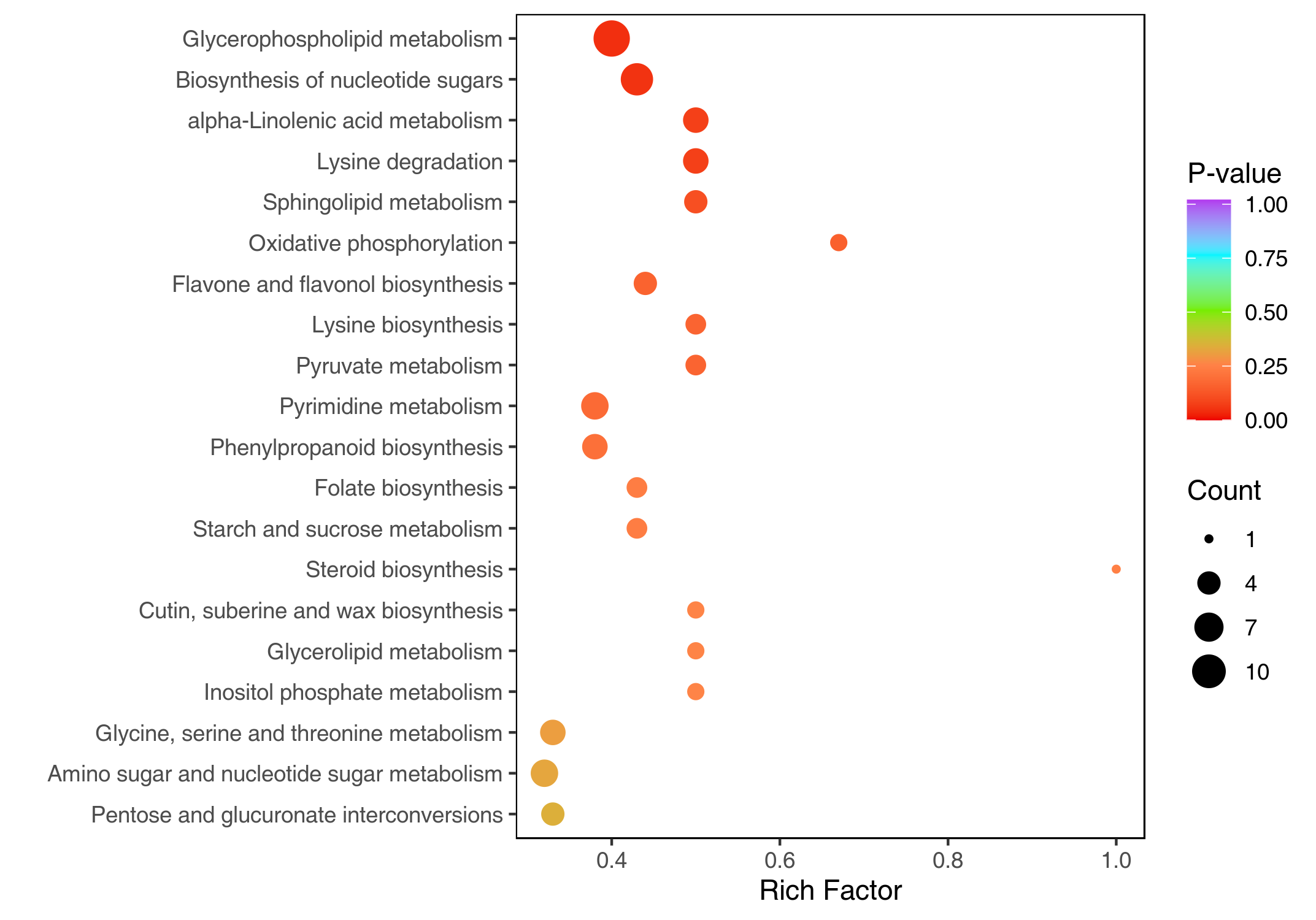

Supplement: Supplementary file 1 [file plants-14-02031-s001.zip › Supplementary Files/Supplementary Pictures/Figure S7-KEGG classification and enrichment/Figure S7f-E_vs_A_KEGG_Enrichment_P-value.png]

Cluster Dendrogram

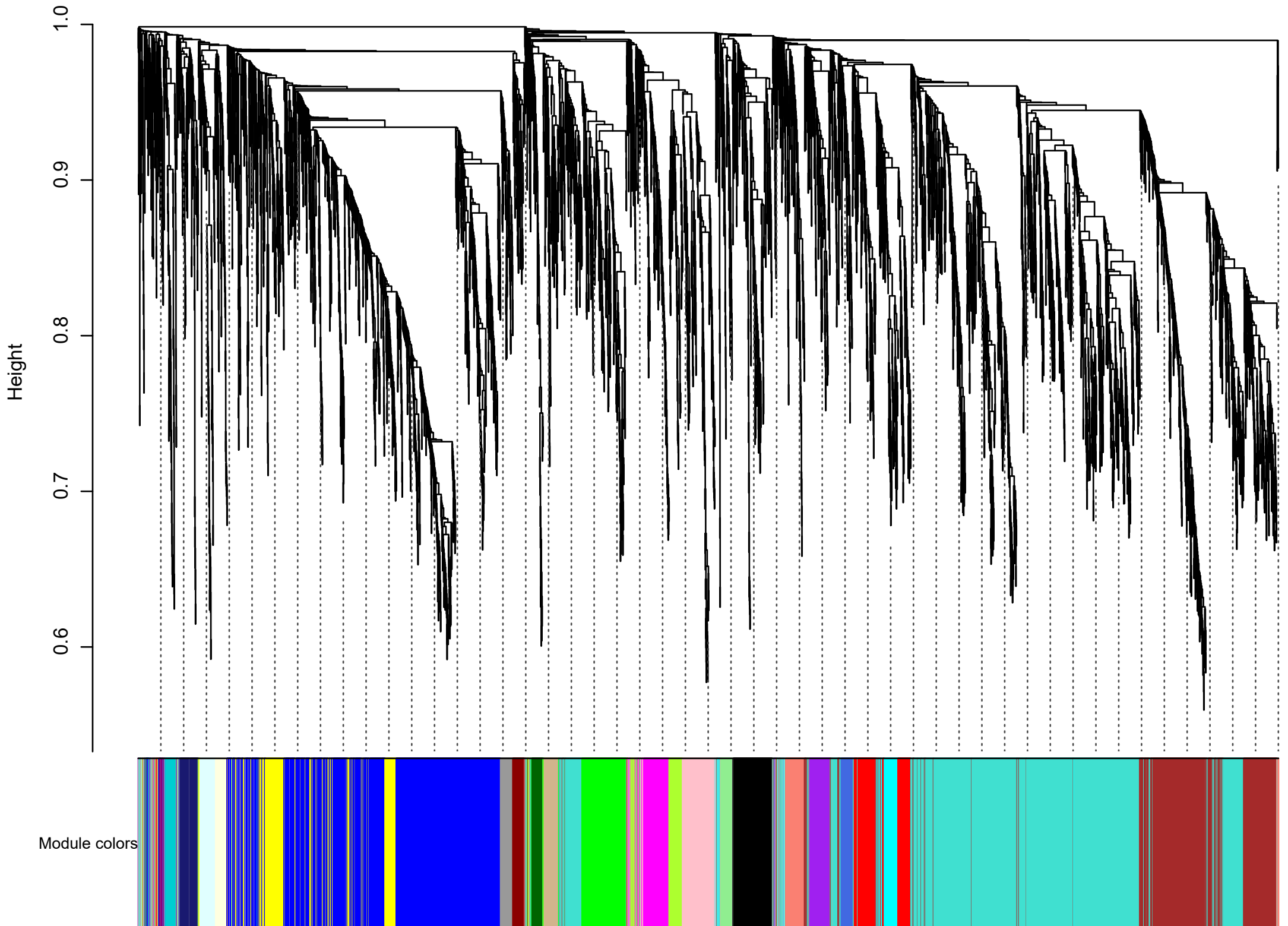

Supplement: Supplementary file 1 [file plants-14-02031-s001.zip › Supplementary Files/Supplementary Pictures/Figure S8-WGCNA Cluster dendrogram and Heatmap of the identified modules(Meta)/Figure S8 Cluster dendrogram.pdf]

Network heatmap plot for selected genes

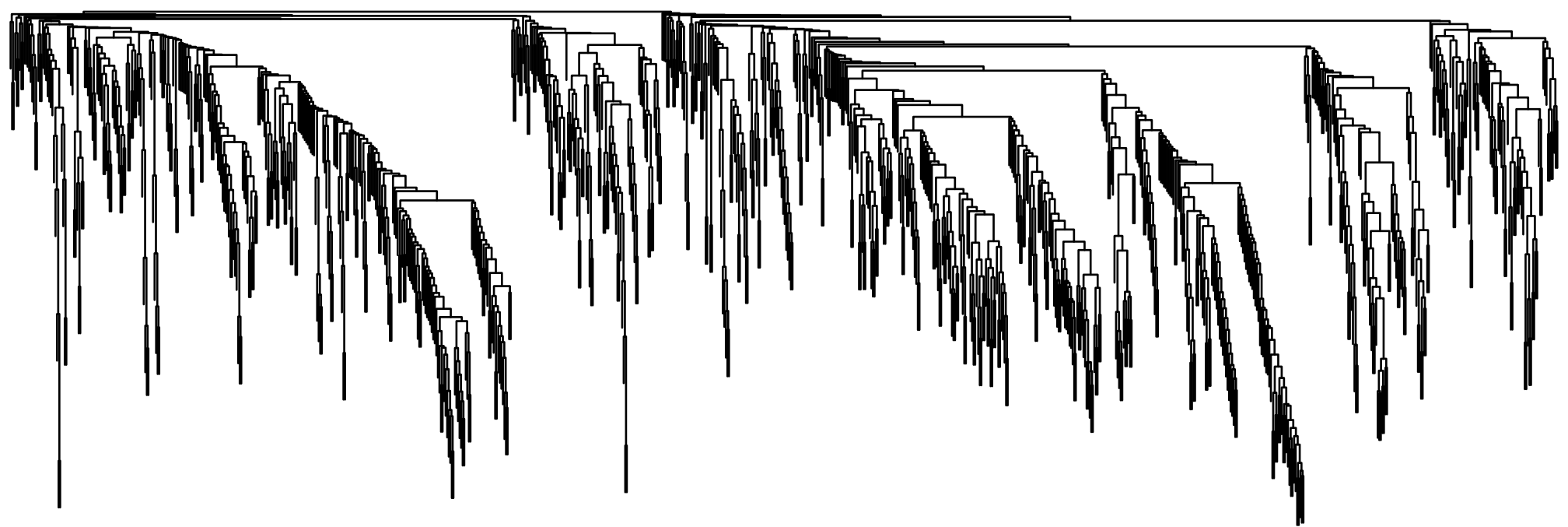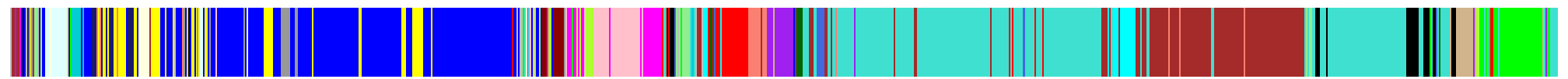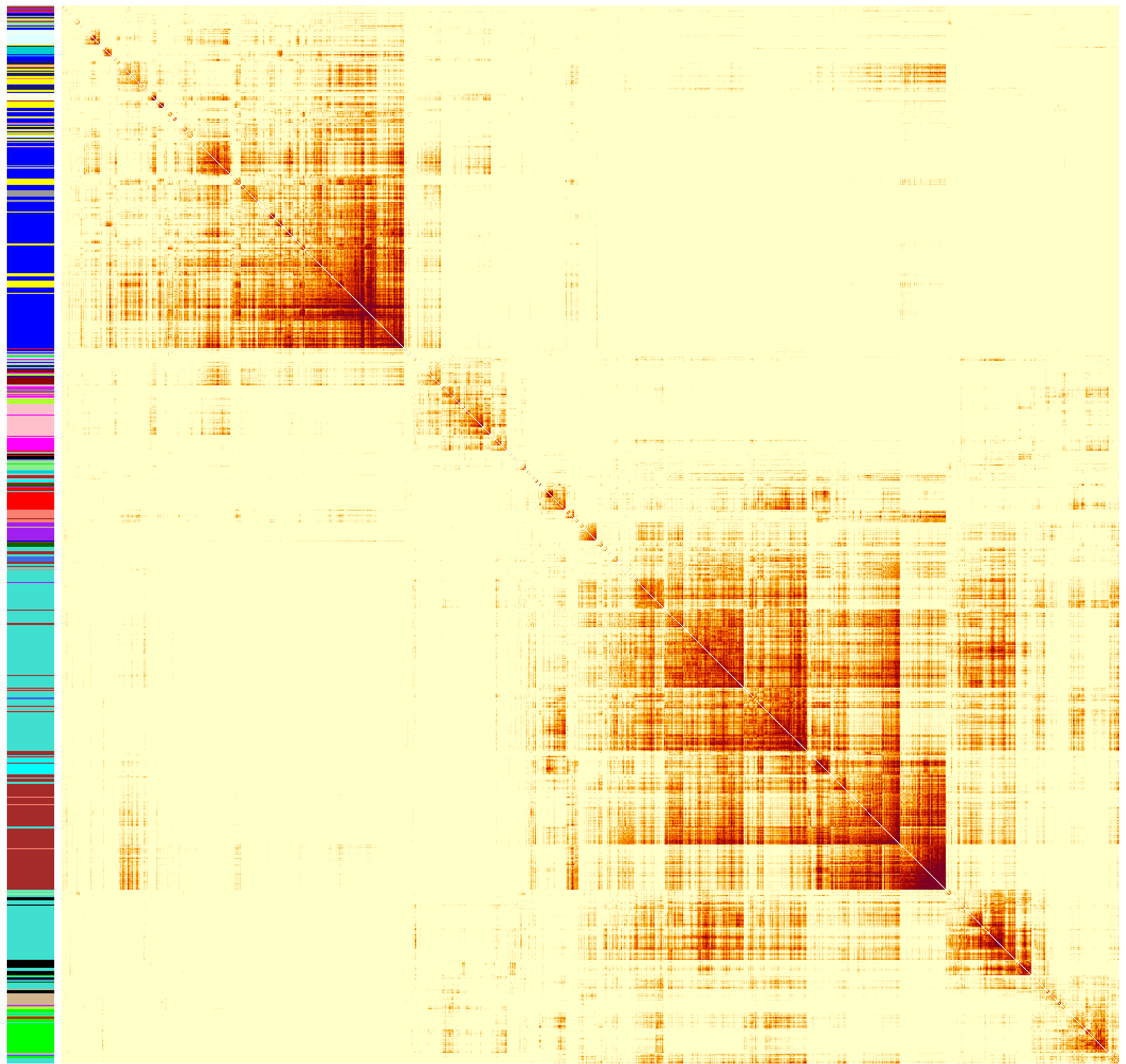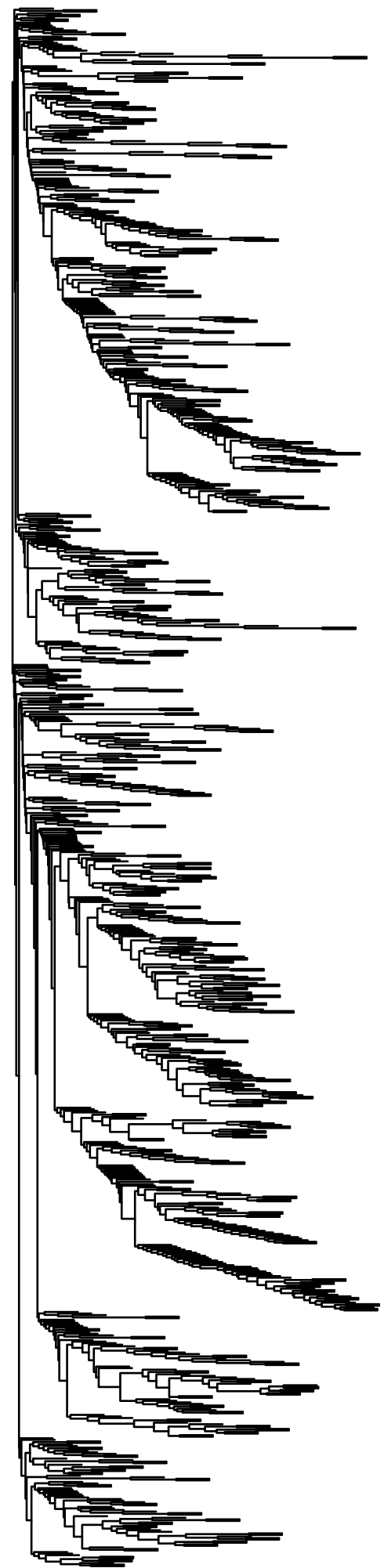

Supplement: Supplementary file 1 [file plants-14-02031-s001.zip › Supplementary Files/Supplementary Pictures/Figure S8-WGCNA Cluster dendrogram and Heatmap of the identified modules(Meta)/Figure S8 Heatmap of the identified modules.pdf]

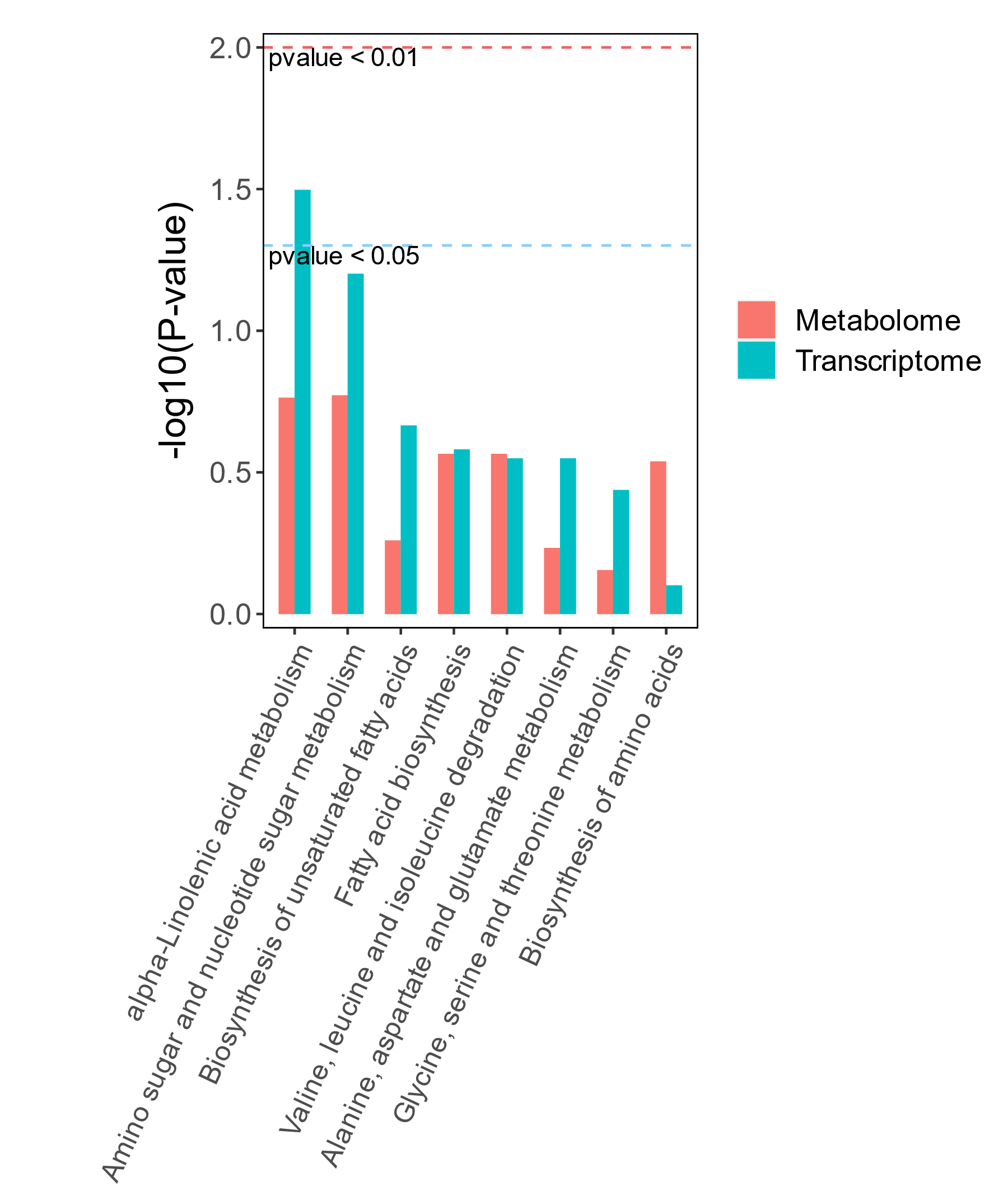

Supplement: Supplementary file 1 [file plants-14-02031-s001.zip › Supplementary Files/Supplementary Pictures/Figure S9-Common analysis/Figure S9a-B_vs_A_common_KEGG_enrichment_column.png]

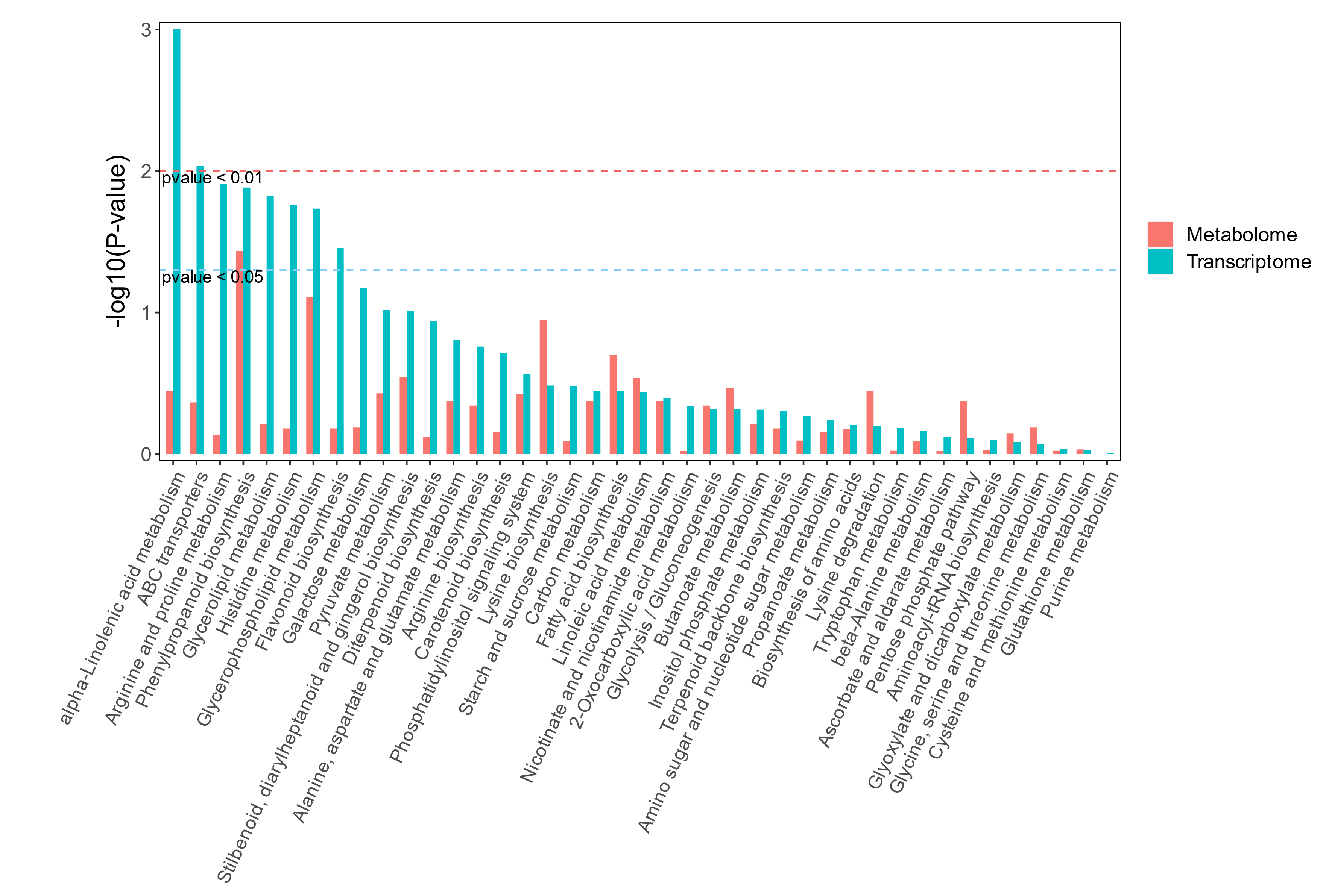

Supplement: Supplementary file 1 [file plants-14-02031-s001.zip › Supplementary Files/Supplementary Pictures/Figure S9-Common analysis/Figure S9b-C_vs_A_common_KEGG_enrichment_column.png]

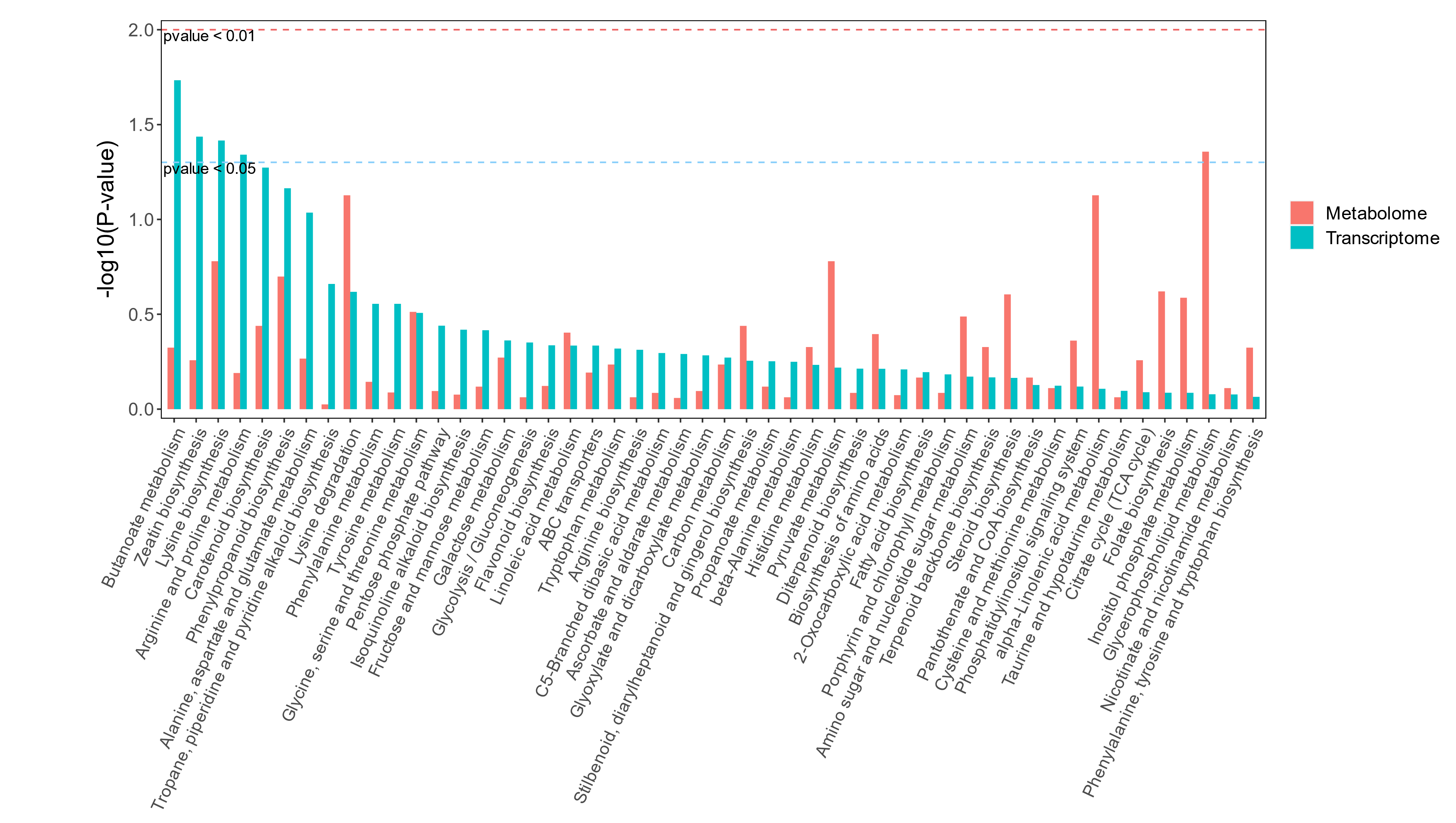

Supplement: Supplementary file 1 [file plants-14-02031-s001.zip › Supplementary Files/Supplementary Pictures/Figure S9-Common analysis/Figure S9c-E_vs_A_common_KEGG_enrichment_column.png]

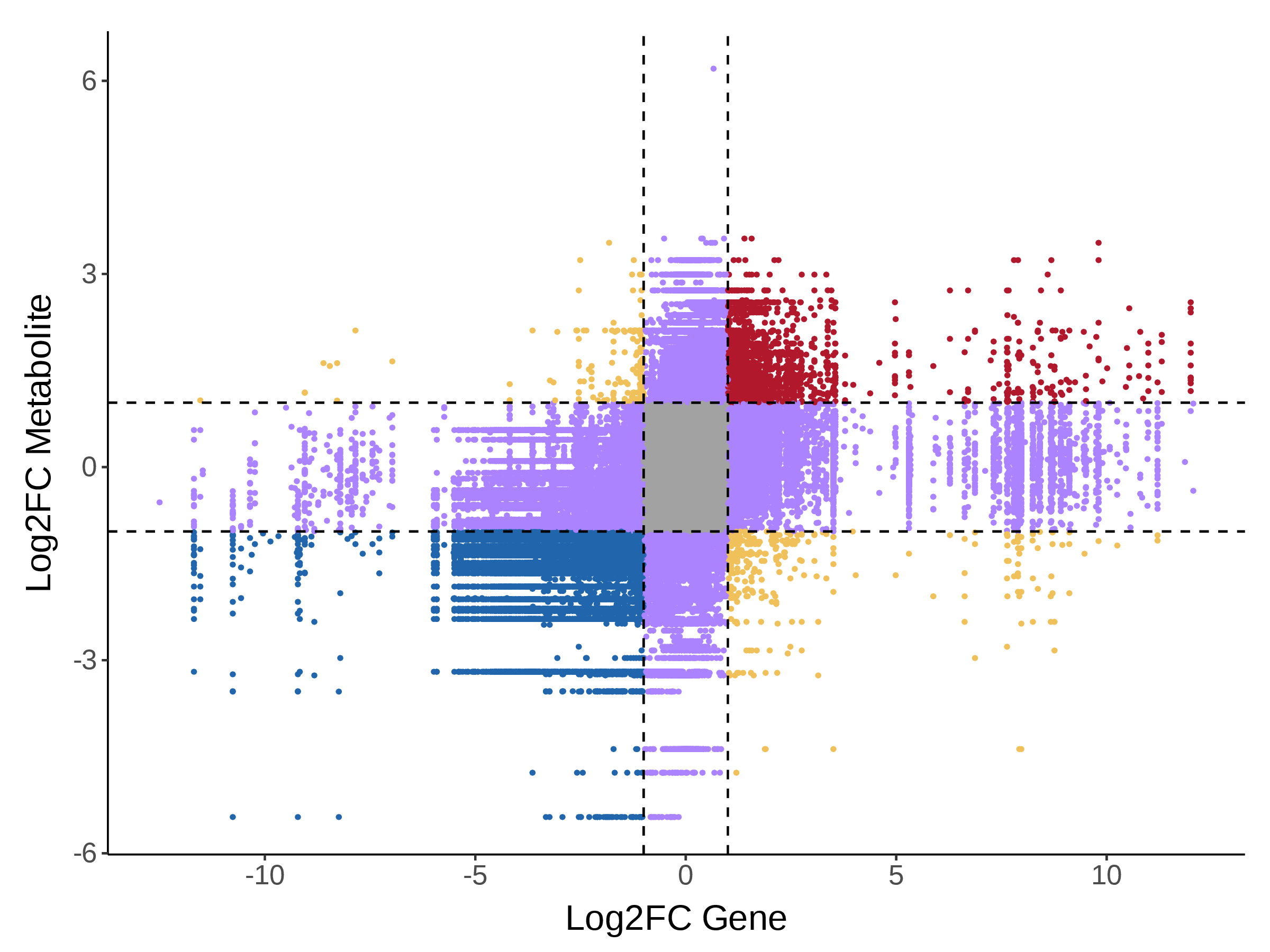

Supplement: Supplementary file 1 [file plants-14-02031-s001.zip › Supplementary Files/Supplementary Pictures/Figure S9-Common analysis/Figure S9d-B_vs_A_ninequadrants.png]

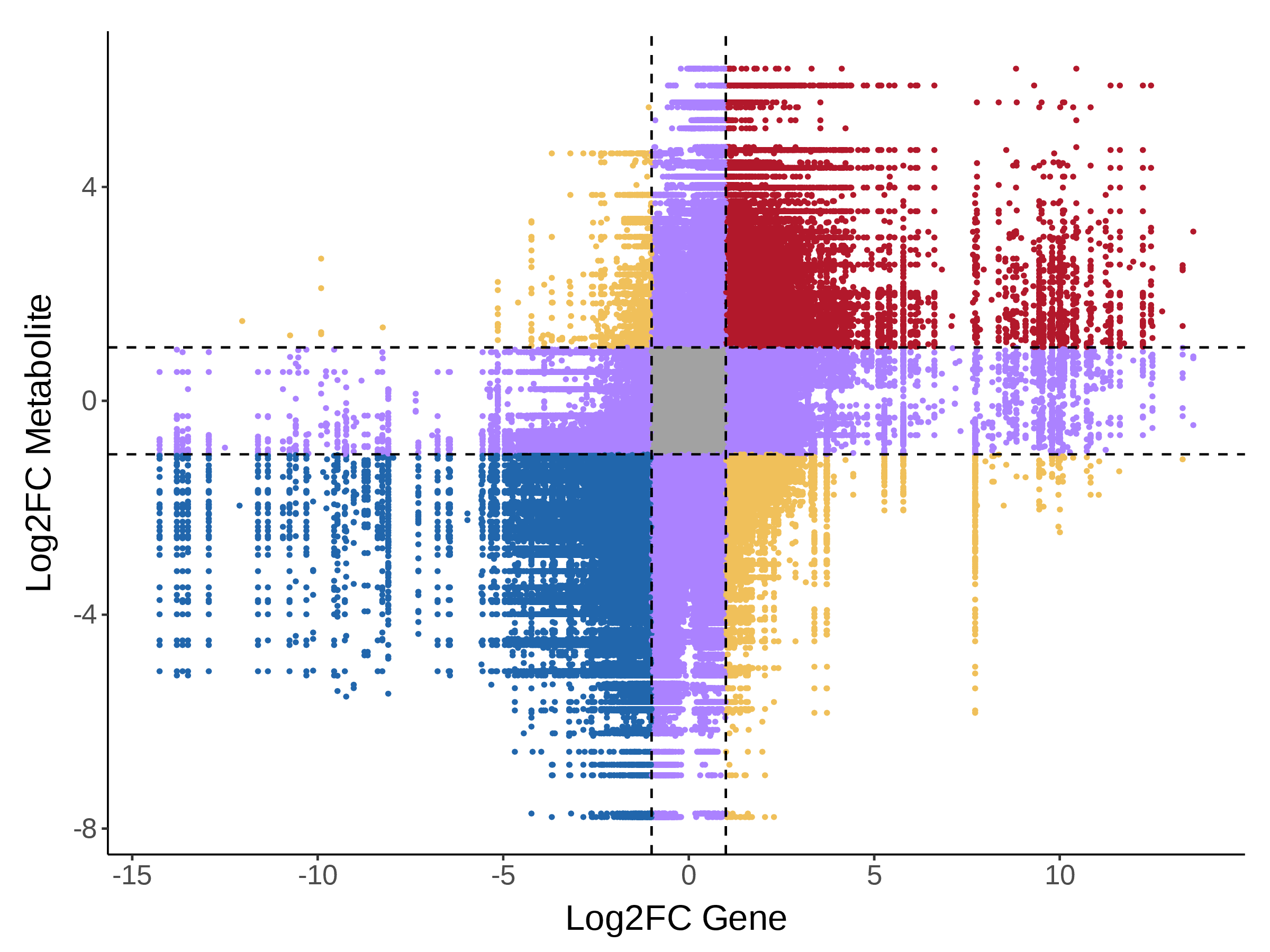

Supplement: Supplementary file 1 [file plants-14-02031-s001.zip › Supplementary Files/Supplementary Pictures/Figure S9-Common analysis/Figure S9e-C_vs_A_ninequadrants.png]

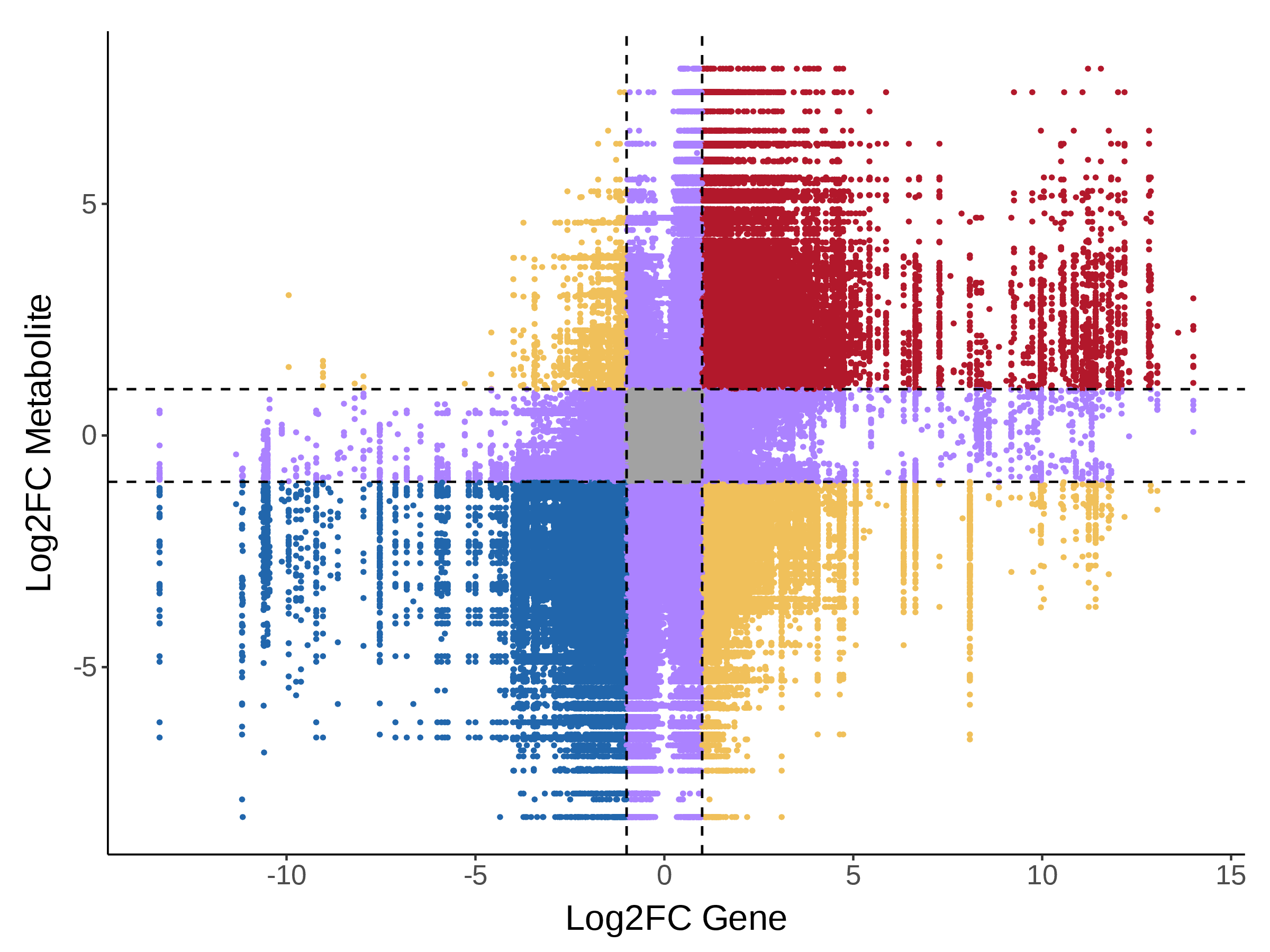

Supplement: Supplementary file 1 [file plants-14-02031-s001.zip › Supplementary Files/Supplementary Pictures/Figure S9-Common analysis/Figure S9f-E_vs_A_ninequadrants.png]

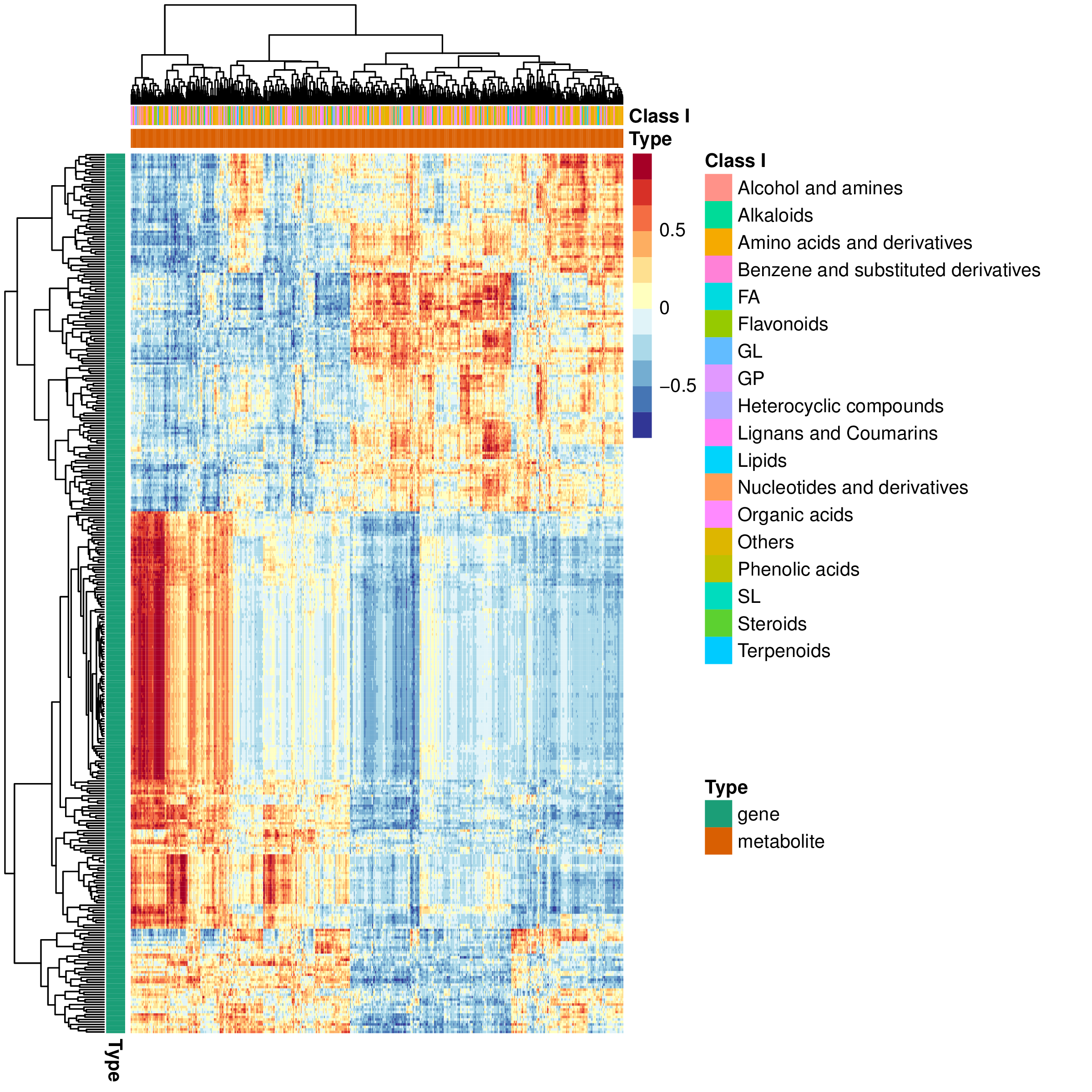

Supplement: Supplementary file 1 [file plants-14-02031-s001.zip › Supplementary Files/Supplementary Pictures/Figure S9-Common analysis/Figure S9g-B_vs_A_correlation_heatmap.png]

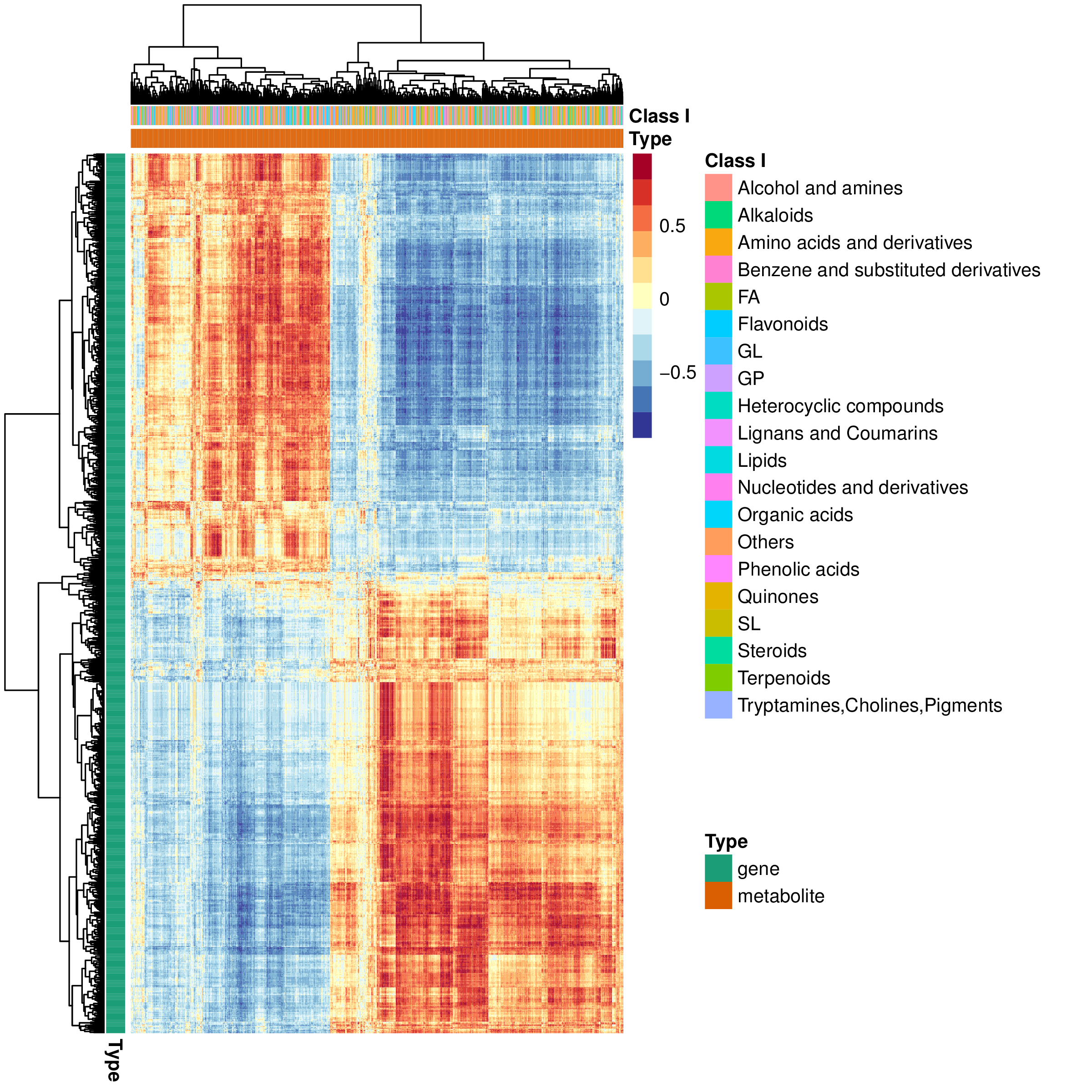

Supplement: Supplementary file 1 [file plants-14-02031-s001.zip › Supplementary Files/Supplementary Pictures/Figure S9-Common analysis/Figure S9h-C_vs_A_correlation_heatmap.png]

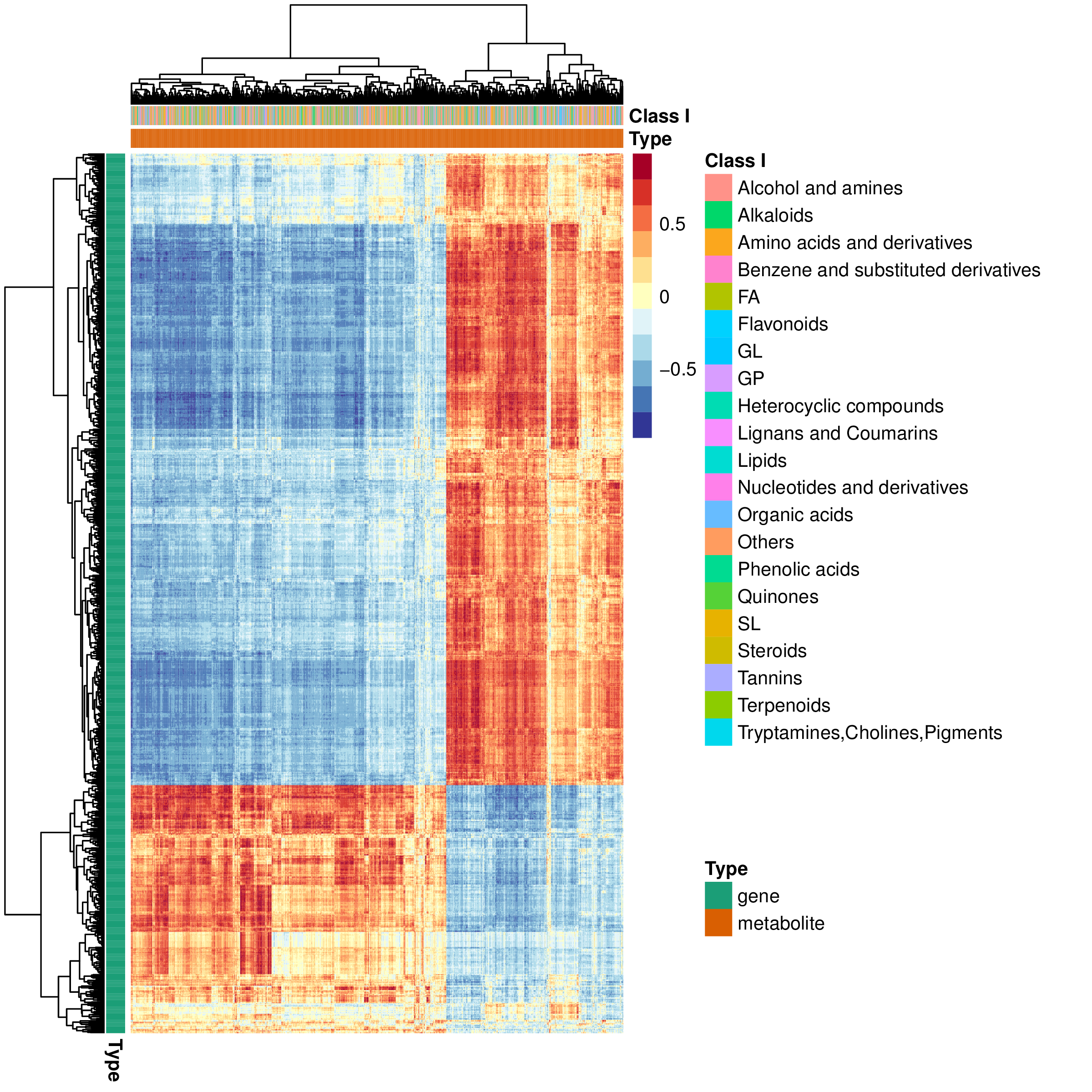

Supplement: Supplementary file 1 [file plants-14-02031-s001.zip › Supplementary Files/Supplementary Pictures/Figure S9-Common analysis/Figure S9i-E_vs_A_correlation_heatmap.png]

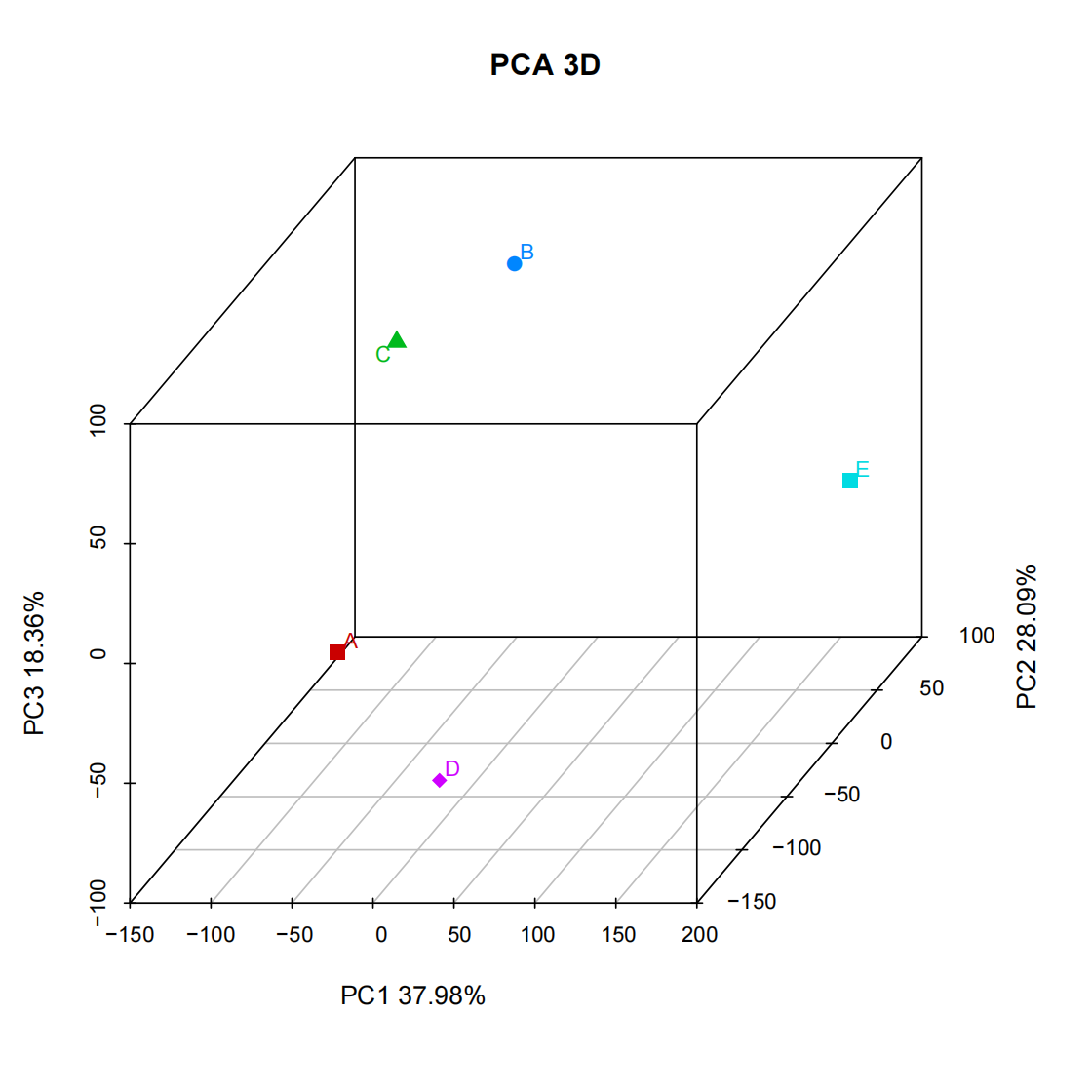

Supplement: Supplementary file 1 [file plants-14-02031-s001.zip › Supplementary Files/Supplementary Pictures/FigureS1-PCA of transcriptome data from with and without saline stress.png]
